# Palladium-catalyzed substitution of (coumarinyl)methyl acetates with C-, N-, and S-nucleophiles

**DOI:** 10.3762/bjoc.8.133

**Published:** 2012-07-27

**Authors:** Kalicharan Chattopadhyay, Erik Fenster, Alexander J Grenning, Jon A Tunge

**Affiliations:** 1 Department of Chemistry, University of Kansas, 1251 Wescoe Hall Drive, Lawrence, KS 66045-7528, USA; 2 KU Chemical Methodologies and Library Development Center of Excellence, University of Kansas, 1501 Wakarusa Drive, Lawrence, Kansas 66047, USA

**Keywords:** benzylation, catalysis, coumarin, chemical diversity, decarboxylative, palladium, substitution

## Abstract

The palladium-catalyzed nucleophilic substitution of (coumarinyl)methyl acetates is described. The reaction proceeds though a palladium π-benzyl-like complex and allows for many different types of C-, N-, and S-nucleophiles to be regioselectively added to the biologically active coumarin motif. This new method was utilized to prepare a 128-membered library of aminated coumarins for biological screening.

## Introduction

Coumarins are privileged chemical motifs found in many natural products and drug molecules [1–15]. Because of their biological significance, there have been many classic and modern methods developed for the synthesis of these useful core structures [16–24]. Due to the ambiphilic nature of the heterocyclic ring of coumarin, this core-structure undergoes a diverse array of coupling reactions, such as halogenations [25], cycloadditions [26–32], conjugate additions [33–37] and transition-metal-catalyzed C–H activation/coupling reactions [38–44].

**Figure 1:**
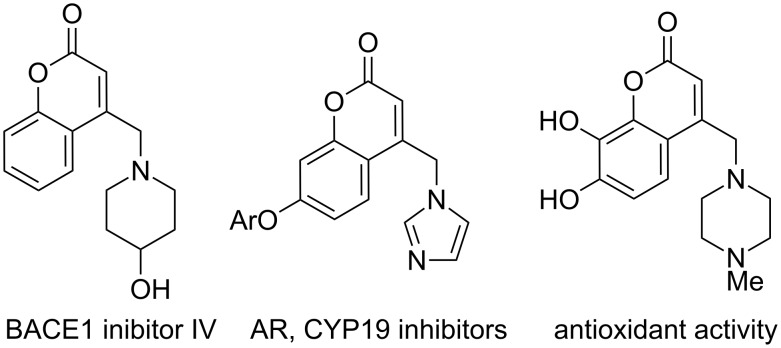
Representative biologically active aminomethylcoumarins.

**Scheme 1:**
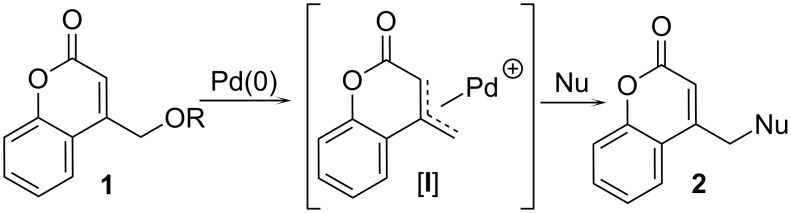
Approach to diversely substituted coumarins.

Substituted methylcoumarins, including aminomethylcoumarins are important biologically active motifs (Figure 1) [6–15]. Substitution of methylcoumarins to form compounds akin to **2** typically utilizes the corresponding halomethylcoumarin and highly stabilized nucleophiles or amines [6–15]. Due to the sensitivity and toxicity of related benzyl halides, there has been interest in catalytically activating hydroxymethylarene and heteroarenes (e.g., benzyl alcohol derivatives) toward reactions with nucleophiles [45–55]. In this realm, we [53,55] and others [54] have focused efforts on catalyzing benzylic substitutions with less-stabilized (DMSO p*K*_a_ ~ 20–30) nucleophiles through decarboxylative coupling. In the present context, we hypothesized that a broad diversity of nucleophiles could be added to the (coumarinyl)methyl core through palladium-catalyzed couplings of hydroxymethylcoumarin derivatives. Herein we report that hydroxymethylcoumarin derivatives of type-**1** undergo selective nucleophilic substitution at the exo-methyl position with C-, N-, and S-based nucleophiles by using palladium(0) as a catalyst (Scheme 1). In addition, given the known biological activity of aminomethylcoumarins, we prepared a 128-member library of aminated coumarins using rapid automated synthesis.

## Results and Discussion

**Scheme 2:**
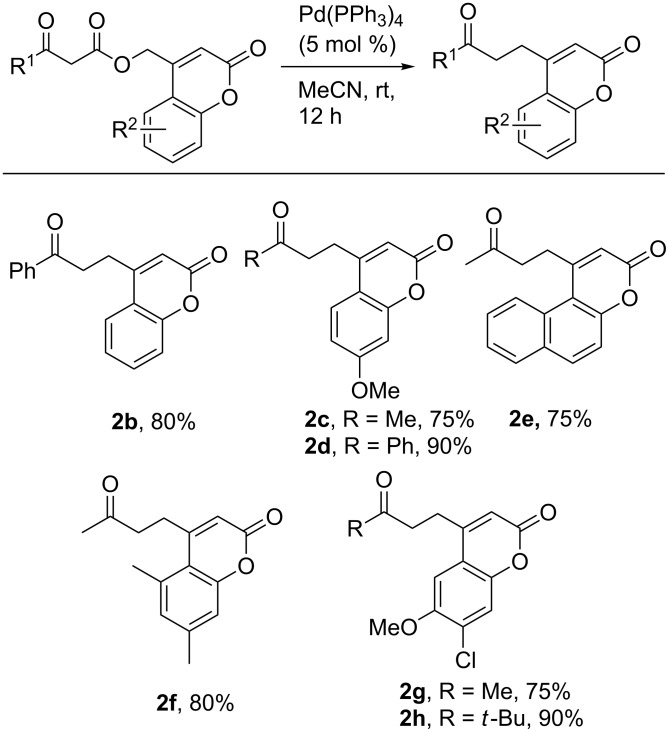
Scope of the decarboxylative coupling.

To begin investigating the diversification of hydroxymethylcoumarins, we chose to investigate their decarboxylative couplings of enolates. We have previously shown that decarboxylative benzylation (DcB) is a useful method for the addition of less-stabilized enolate anions to a benzyl functionality [46,53–55]. Thus, we envisioned being able to add various ketone enolates to the exo-methyl position of the coumarin core by this method (Table 1). Screening of reaction conditions showed that, in contrast to common decarboxylative benzylation conditions (Table 1,entries 1 and 2) under which nonpolar solvents give the best DcB, the selective mono-alkylation of the enolate proceeds in highest yield in acetonitrile (**2a**, Table 1, entry 3). Bidentate ligated palladium complexes gave mixed results: the Pd/dppf complex (Table 1,entry 4) catalyzed the reaction smoothly, while little product was seen on using the Pd/BINAP complex (Table 1,entry 5). Under the best conditions (5 mol % Pd(PPh_3_)_4_, MeCN, rt, Table 1, entry 3), a variety of enolate nucleophiles could be selectively coupled with coumarin electrophiles (Scheme 2). For example, acetone, acetophenone and 1,1,1-trimethylacetone enolates can all be generated and coupled with the coumarin electrophile. Importantly, selective monobenzylation was achieved for each example. Regarding the coumarin moiety, the decarboxylative coupling was compatible with a variety of simple substitutions, including methoxy (**2c,d,g,h**), chloro (**2g,h**) and naphthyl (**2e**) coumarins.

**Table 1:** Reaction development for the coupling of in situ generated enolates and coumarin π-benzyl complexes.

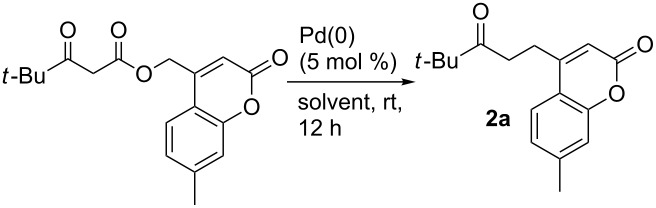			

Entry	Catalyst	Solvent	Conv. (%)

1	Pd(PPh_3_)_4_	toluene	68
2	Pd(PPh_3_)_4_	THF	70
3	Pd(PPh_3_)_4_	MeCN	95 (88% yield)
4	Pd_2_dba_3_, dppf	MeCN	88
5	Pd_2_dba_3_, BINAP	MeCN	10

While the decarboxylative coupling worked well for the couplings of relatively nonstabilized enolate nucleophiles, we wished to find a more universal starting material that would allow a broader range of nucleophiles to be coupled to the coumarin core in an intermolecular fashion. Such a method was deemed necessary for rapid diversification and chemical library synthesis. Thus, we chose to investigate the Pd-catalyzed benzylic substitution reaction of (coumarinyl)methyl acetates with various nucleophiles. In the forthcoming sections, the coupling of this coumarin template to arylboronic acids [56–61], amines [47] and arylsulfinates is described [62]. Moreover, compared to related methods for palladium-catalyzed benzylic substitution that require specialized ligands [45,47,60–62], we report that coumarin π-benzyl formation is easily achieved with simple PPh_3_ ligated palladium.

Regarding the development of the Suzuki-like coupling reaction of (coumarinyl)methyl acetate and arylboronic acids, we screened reaction conditions for the coupling of coumarin **1a** with phenylboronic acid (Table 2). We were pleased to find that the reaction progressed reasonably well to **3a** under various conditions. For example, inorganic bases such as K_2_CO_3_, K_3_PO_4_, and KF all effected the reaction equally well in methanol. A brief screen of solvents showed that the nonpolar aprotic solvent 1,4-dioxane gave the highest yields of the coupling product (Table 2, entry 6). Lastly, in the absence of added base, the acetate generated upon π-benzyl formation also promoted the desired coupling, albeit in lower yield over the time frame allowed (12 h, Table 2, entry 7).

**Table 2:** Palladium-catalyzed coupling of coumarinyl acetate and phenylboronic acid.

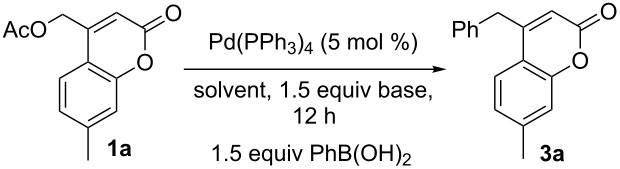				

Entry	Solvent	*T* (°C)	Base	Conv. (%)

1	MeOH	65	K_2_CO_3_	65
2	MeOH	65	KF	59
3	MeOH	65	K_3_PO_4_	65
4	toluene	90	K_2_CO_3_	63
5	THF	65	K_2_CO_3_	63
6	dioxane	90	K_2_CO_3_	85
7	dioxane	90	–	45

**Scheme 3:**
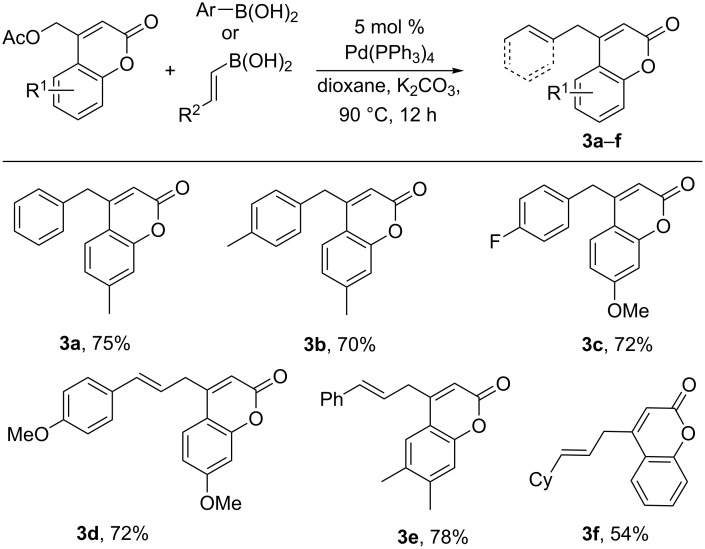
Scope of Suzuki-coupling of coumarinyl acetates.

With the optimal conditions in hand, we next tested the scope of the reaction using various boronic acid and coumarinyl acetates (Scheme 3). Regarding the boronic acid coupling partner, modifications including fluoro and alkyl substitution were tolerated. In addition to aryl boronic acids (**3a**–**c**), the reaction was also extended to couplings of vinyl boronic acids having varied electronic properties (**3d**–**e**). As before, various simple substitutions and electronic changes were tolerated on the coumarin core.

**Scheme 4:**
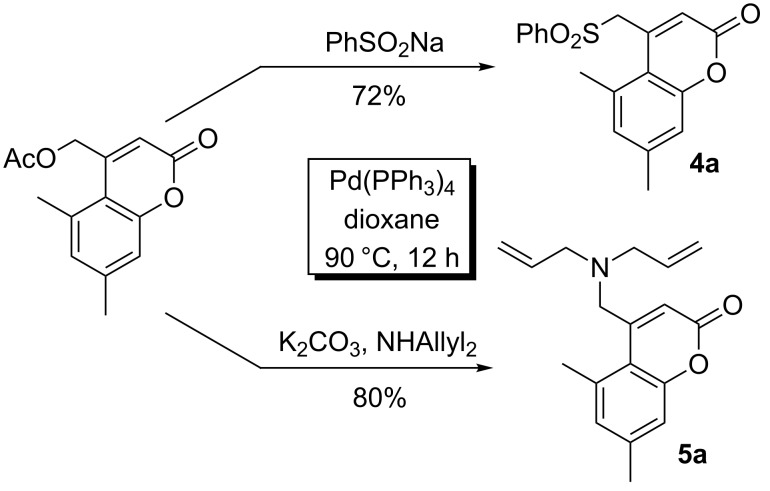
Coupling of (coumarinyl)methyl acetates with N- and S-nucleophiles.

In addition to the above Suzuki couplings, acetoxymethylcoumarins were found to undergo palladium-catalyzed substitution with sulfinates and secondary amines under the previously developed palladium-catalysis conditions (Scheme 4). Importantly, related aminomethylcoumarins have been shown to have significant biological activity [6–15].

**Scheme 5:**
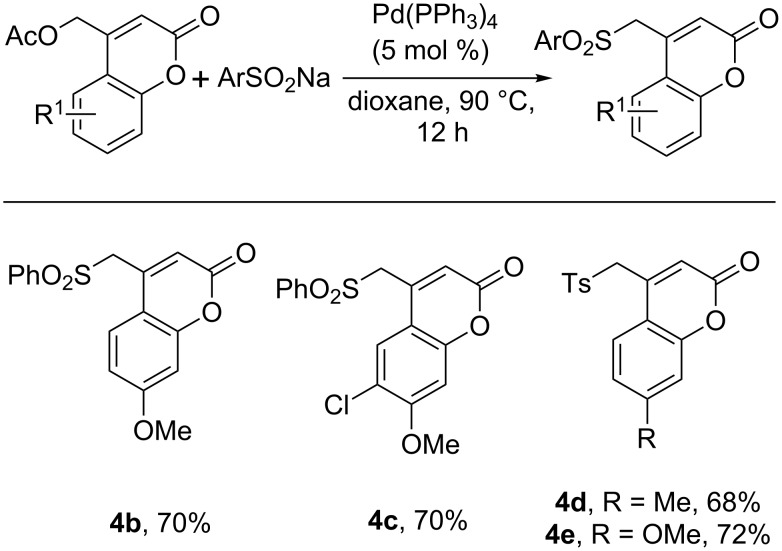
Scope of the coumarinyl acetate and aryl sulfinate coupling reaction.

Regarding the coupling reaction between aryl sulfinates and the coumarinyl acetate, the reaction could be performed without the addition of an external base, since the aryl sulfinate is administered as its anion (Scheme 5). Phenyl (**4a**–**c**) and tolyl (**4d**–**e**) sulfinates were viable coupling partners, giving the product sulfones in good yield. As previously noted, the coumarin core tolerated various simple electronic (**4b**,**c**,**e**) and alkyl (**4a**,**c**,**d**) substitution patterns.

**Scheme 6:**
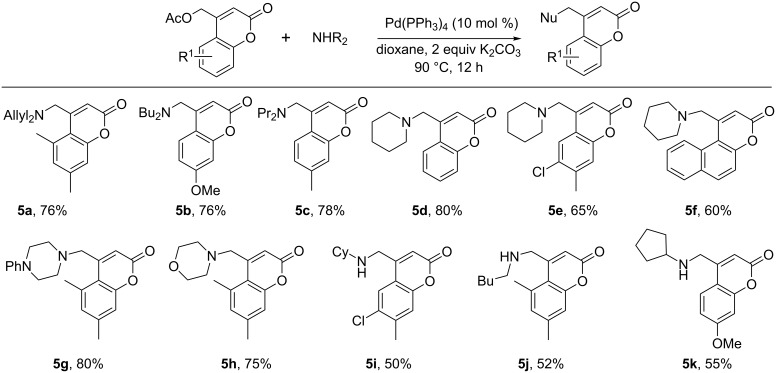
Scope of the coumarinyl acetate and amine coupling reaction.

Since (coumarinyl)methylamines are known to possess interesting biological activity, the scope of the amination was investigated in somewhat more detail. Regarding the scope of the amine and coumarinyl acetate coupling reaction, dialkyl (**5a**–**c**) and the cyclic amines pyrrolidine (**5d**–**5f**), pyrazine (**5g**), and morpholine (**5h**) were competent coupling partners (Scheme 6). Primary amines were also compatible coupling partners, although under these conditions, the reaction never went to full conversion (**5i**–**k**).

**Figure 2:**
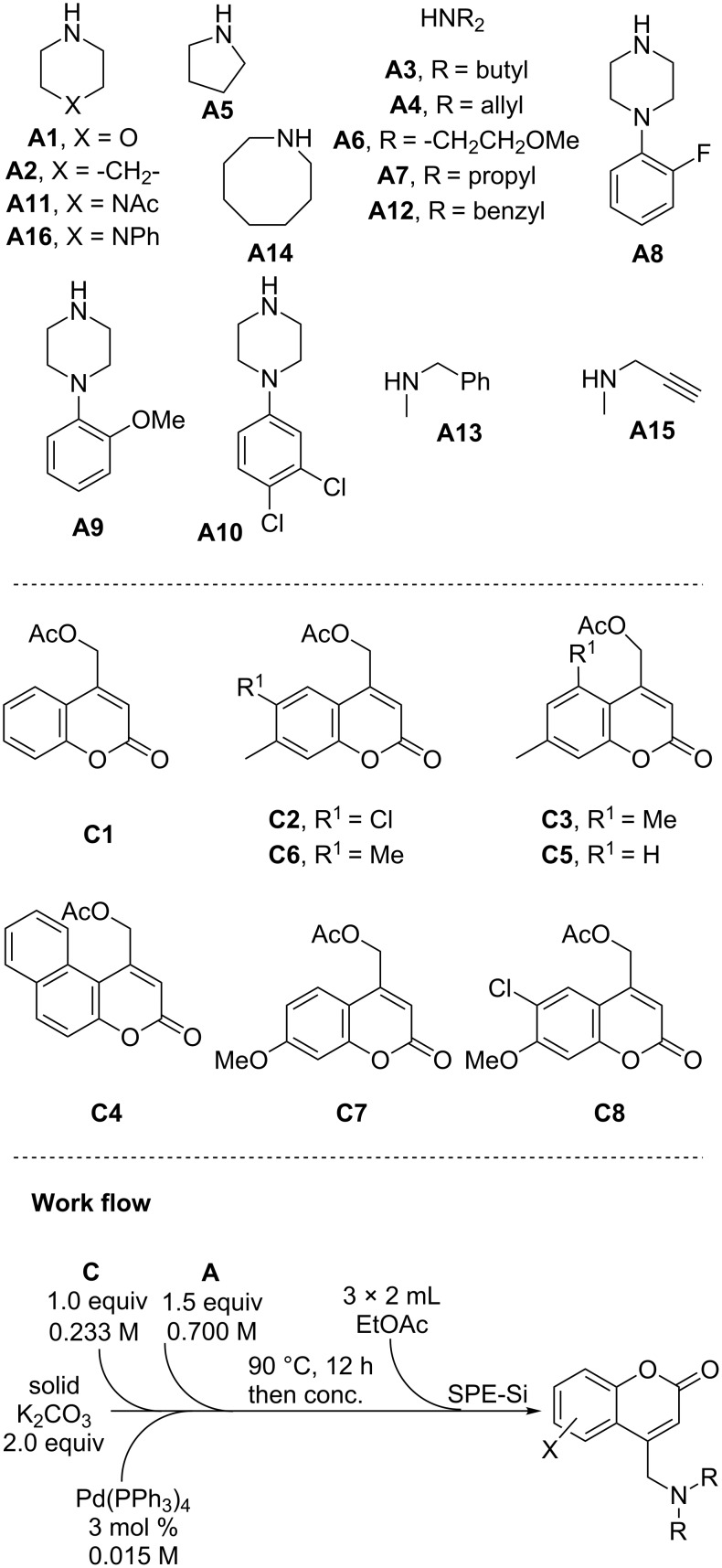
Library planning for amine (**A**) and coumarin (**C**) coupling partners.

As mentioned previously, we were devising this approach to coumarin substitution not only for the sake of new chemical methodology, but also because this approach could easily lead to rapid library synthesis. As aminomethylcoumarins are a biologically active chemical motif [6–15], we set out to produce a 128-member library of aminated coumarins using our method, with the goal of making unique, biologically active molecules (Figure 2). To begin, an 8 × 16 library (coumarin **C1–8** × amine **A1–16**) of aminomethylcoumarins was devised for preparation by using a Chemspeed SLT100 automated synthesizer. For automated synthesis, the procedure and workflow was modified somewhat for optimal yield. Specifically, the loading of Pd was lowered to 3 mol % and the quantity of amine was raised to 1.5 equivalents to ensure complete conversion. Using the workflow shown in Figure 2 for the reaction of coumarin **C3** with diallylamine **A4**, followed by filtration through a silica SPE, produced product **C3A4** in 74% yield in a test case. In addition, the product was determined to contain 2% PPh_3_ impurity. Thus, it was concluded that the entire library should be purified by mass-directed fractionation to ensure high-quality compounds for biological screening.

**Figure 3:**
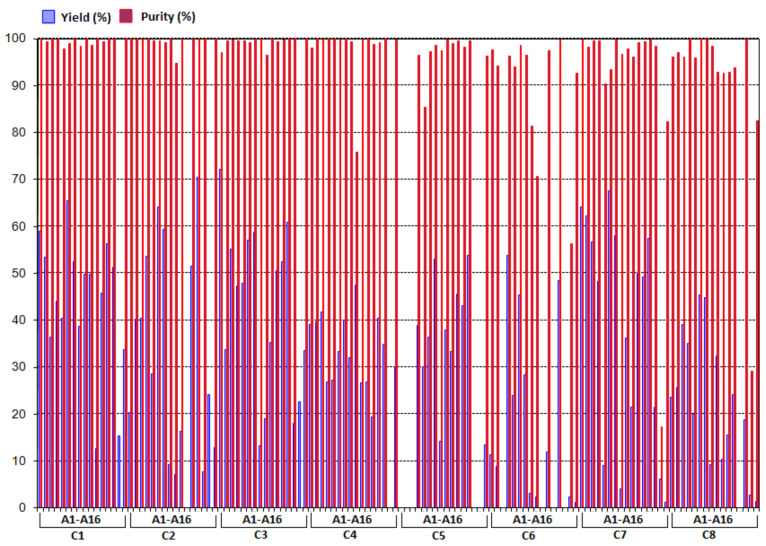
Results for the synthesis of a 128-member library of aminated coumarins by using the Chemspeed SLT100 automated synthesizer. Purified by an automated preparative reverse-phase HPLC (Waters 2767 Mass Directed Fractionation) detected by UV (270 nm). Purity was determined by reverse-phase HPLC (Waters Alliance 2795 system) with peak area (UV) at 214 nm.

Regarding the library, coumarins and amines were chosen with diverse structures and properties in order to access the most chemical space within the aminomethylcoumarin family (Figure 2). The coumarins utilized had various electronic properties and substitution patterns. A polyaromatic coumarin was also screened (**C4**). Similarly, a diverse array of amines was chosen. Aside from simple dialkylamines, cyclic and heteroatom-containing amines were utilized. Amines with various functional groups, such as amides, aromatics, olefins, and alkyne substitution patterns, were also incorporated in this library. The automated library synthesis had a success rate of >85%, with the desired products isolated in high purity [63]. Taking center cuts by using mass-directed fractionation ensured that compounds were isolated in >95% purity; however, purity was achieved at some expense to the isolated yields, which were typically lower (5–75%) than those achieved in batch reactions. While most compounds were obtained in sufficient quantity for biological screening, the yields were variable (Figure 3). In particular, coumarins **C6**, **C2** and **C8** provided lower average yields than the other coumarin cores. Thus, it appears that 6,7-substitution of the coumarin core is somewhat problematic. In addition, analysis of the library yields indicates that *N*-arylpiperazines **A8** and **A9** in addition to propargylamine **A15** were the most problematic. Perhaps unsurprisingly, the amine that provided the highest yields and highest success rate was diallyl amine, which was used for initial validation of the Chemspeed method (see above). In addition, piperidine and the simple open-chain dialkyl amines also provided good yields and high success rates.

## Conclusion

In conclusion, we have developed a simple and general strategy for the palladium-catalyzed substitution of coumarins selectively at an exomethyl position. This approach allowed the coupling of various C-,N-, and S-based nucleophiles under mild conditions. We also utilized this approach in the synthesis of a 128-member chemical library appropriate for biological screening.

## Supporting Information

File 1 Experimental data and ^1^H, ^13^C, and IR, and HRMS data for new compounds produced in batch.

File 2 Detailed results of the library analysis are likewise included.
