# Supplementary material for: Palladium-catalyzed substitution of (coumarinyl)methyl acetates with C-, N-, and S-nucleophiles
Source: Beilstein J Org Chem. 2012 Jul 27;8:1200–7. doi: 10.3762/bjoc.8.133 (PMC3458738; doi:10.3762/bjoc.8.133)
Supplement: File 1 — Experimental data and 1H, 13C, and IR, and HRMS data for new compounds produced in batch. [file Beilstein_J_Org_Chem-08-1200-s001.pdf]

Supporting Information
for

Palladium-catalyzed substitution of (coumarinyl)methyl acetates with C-, N-, and S-nucleophiles

Kalicharan Chattopadhyay[1,2], Erik Fenster[2], Alexander J. Grenning[1] and Jon A. Tunge[1,2*]

[1]Department of Chemistry, University of Kansas, 1251 Wescoe Hall Drive, Lawrence, KS 66045-7528, USA and [2]KU Chemical Methodologies and Library Development Center of Excellence, University of Kansas, 1501 Wakarusa Drive, Lawrence, Kansas 66047, USA.

Email: Jon A. Tunge - tunge@ku.edu

Corresponding author

Experimental data and ^{1}H, ^{13}C, and IR, and HRMS data for new compounds produced in batch.

Table of Contents:

General information: 1
Experimental procedures: 2
Spectral data for compounds 2a-h, 3a-f, 4a-e, 5a-k: 3
Library experimental/representative spectral data and NMR reprints: 18

General information:

All reactions were run in flame-dried glassware under an Ar atmosphere using standard Schlenk techniques. Commercially available reagents were used without additional purification. Compound purification was effected by flash chromatography using 230 × 400 mesh, 60 Å porosity silica gel. ^{1}H NMR and ^{13}C NMR spectra were obtained on a Bruker Avance 400 and 500 DRX spectrometer and referenced to residual protio solvent signals. Structural assignments are based on ^{1}H, ^{13}C, IR, and HRMS spectroscopies.

General Procedure for decarboxylative benzylation (DcB) reaction of coumarinyl acetates:

In a flame-dried Schlenk tube under argon, $Pd(PPh_3)_4$ (5 mol %) was added to a acetonitrile solution (1.5 mL) of coumarinyl acetate (0.16 mmol), and the solution was stirred at room temperature for 12–15 h. The reaction mixture was then concentrated in vacuo and purified directly by column chromatography with hexane/ethyl acetate (90:10) as eluent.

General procedure for the Suzuji-coupling of coumarinyl acetates and aryl/vinyl boronic acids:

In a flame-dried Schlenk tube under argon, $Pd(PPh_3)_4$ (5 mol %) was added a dioxane solution (2 mL) of (coumarinyl)methyl acetates (0.5 mmol), boronic acid (2 equiv), K_2CO_3 (1.5 equiv), and the solution was stirred at 90 °C for 12–15 min. The reaction mixture was then concentrated in vacuo and purified directly by column chromatography with hexane/ethyl acetate as eluent.

General procedure for the Pd-catalyzed coupling of coumarinyl acetates and sulfinates:

In a flame-dried Schlenk tube under argon, $Pd(PPh_3)_4$ (5 mol %) was added to a dioxane solution (2 mL) of (coumarinyl)methyl acetates (0.5 mmol) and sodium salt of arylsulfinate, and the solution stirred at 90 °C for 12 h. The reaction mixture was then concentrated in vacuo and purified directly by column chromatography with hexane/ethyl acetate as eluent.

General procedure for the Pd-catalyzed coupling of coumarinyl acetates and amines:

In a flame-dried Schlenk tube under argon, $Pd(PPh_3)_4$ (10 mol %) was added a dioxane solution (2 mL) of (coumarinyl)methyl acetates (0.5 mmol), amine (2 equiv), K_2CO_3 (2 equiv), and the solution stirred at 90 °C for 12–15 h (secondary amine) or 24 h (primary amine). The reaction mixture was then concentrated in vacuo and purified directly by column chromatography with hexane/ethyl acetate as eluent.

Spectral data for compounds 2a–h, 3a–f

2a: yellow viscous liquid

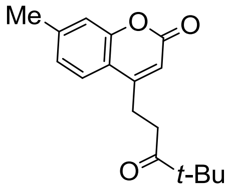

^{1}H NMR (400 MHz, $CDCl_3$):

δ ppm 7.53 (d, *J* = 8 Hz, 1H), 7.28–7.12 (m, 2H), 6.20 (s, 1H), 3.05–3.01 (m, 2H), 2.91–2.88 (m, 2H), 2.46 (s, 3H), 1.18 (s, 9H)

^{13}C NMR (125 MHz, $CDCl_3$):

δ ppm 213.56, 161.11, 155.35, 153.79, 143.05, 125.54, 123.82, 117.52, 116.62, 112.81, 44.20, 34.67, 26.41, 25.56, 21.58

IR (CH_2Cl_2): ν_{max} 1718, 1620 cm^{-1}

HRMS: Calcd. for $C_{17}H_{21}O_3$ (M + H), 273.1491; Found, 273.1365

2b: viscous liquid

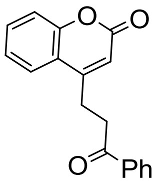

^{1}H NMR (500 MHz, $CDCl_3$):

δ ppm 7.91–7.89 (m, 2H), 7.62 (dd, *J* = 8, 1.5 Hz, 1H), 7.53–7.39 (m, 4H), 7.28–7.22 (m, 2H), 6.25 (s, 1H), 3.35–3.32 (m, 2H), 3,20– 3.17 (m, 2H)

^{13}C NMR (100 MHz, $CDCl_3$):

δ ppm 195.11, 158.47, 152.71, 151.42, 133.96, 131.33, 129.60, 126.52, 125.75, 122.12, 121.89, 116.78, 115.13, 111.81, 34.08, 23.18.

IR (CH_2Cl_2): 1724, 1685 cm^{-1}

HRMS: Calcd. for $C_{18}H_{14}O_3Na$ (M + Na), 301.0841; Found, 301.0815

2c: viscous liquid

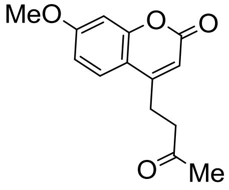

^{1}H NMR (400 MHz, $CDCl_3$):

δ ppm 7. 45–7.43 (m,1H), 6.78–6.76 (m, 1H), 6.70 (s, 1H), 5.98 (s, 1H), 3.78 (s, 3H), 2.94–2.91 (m, 2H), 2.79–2.76 (m, 2H), 2.15 (s, 3H).

^{13}C NMR (100 MHz, $CDCl_3$):

δ ppm 206.02, 162.69, 161.23, 155.49, 154.98, 125.12, 112.53, 112.45, 110.71, 101.11, 55.79, 41.20, 30.08, 25.08.

IR (CH_2Cl_2): 1720, 1605 cm^{-1}.

HRMS: Calcd. for $C_{14}H_{15}O_4$ (M + H), 247.0970; Found, 247.0963

2d: yellow viscous liquid

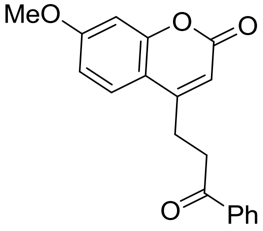

^{1}H NMR (500 MHz, $CDCl_3$):

δ ppm 7.91–7.89 (m, 2H), 7.53–7.50 (m, 2H), 7.42–7.39 (m, 2H), 6.79 (dd, J = 10.0, 5.0 Hz, 1H), 6.75 (s, 1H), 6.09 (s, 1H), 3.79 (s, 3H), 3.33–3.30 (m, 2H), 3.14–3.11(m, 2H).

^{13}C NMR (125 MHz, $CDCl_3$):

δ ppm 197.53, 162.70, 161.26, 155.52, 155.26, 136.28, 133.58, 128.79, 128.03, 125.20, 112.62, 112.47, 110.84, 101.14, 55.78, 36.51, 25.55.

IR (CH_2Cl_2): ν_{max} 1720, 1683, 1612 cm^{-1}.

HRMS: Calcd. for $C_{19}H_{16}O_4Na$ (M + Na), 331.0946; Found, 331.0927.

2e: yellow viscous liquid

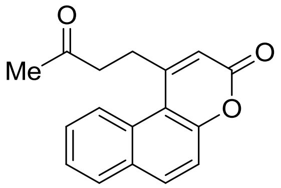

^{1}H NMR (500 MHz, $CDCl_3$):

δ ppm 8.31 (d, *J* = 5 Hz, 1H), 7.88 (d, *J* = 10 Hz, 1H), 7.83 (dd, *J* = 8, 1.5 Hz, 1H), 7.57–7.54 (m, 1H), 7.48–7.45 (m, 1H), 7.36 (d, *J* = 10 Hz, 1H), 6.27 (s, 1H), 3.45–3.42 (m, 2H), 2.85–2.82 (m, 2H). 2.14 (s, 3H).

^{13}C NMR (125 MHz, $CDCl_3$):

δ ppm 204.33, 158.64, 155.08, 153.30, 132.27, 129.76, 128.29, 127.90, 126.65, 123.89, 122.96, 116.33, 113.70, 111.93, 40.20, 29.56, 28.51.

IR (CH_2Cl_2): ν_{max} 1718, 1421 cm^{-1}.

HRMS: Calcd. for $C_{17}H_{15}O_3$ (M + H), 267.1021; Found, 267.1083.

2f: yellow viscous liquid

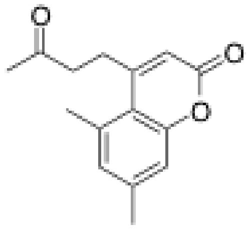

^{1}H NMR (500 MHz, $CDCl_3$):

δ ppm; 6.95 (s, 1H), 6.83 (s, 1H), 6.04 (s, 1H), 3.15–3.12 (m, 2H), 2.75–2.72 (m, 2H), 2.60 (s, 3H), 2.30 (s, 3H), 2.16 (s, 3H).

^{13}C NMR (125 MHz, $CDCl_3$):

δ ppm 205.89, 160.66, 156.75, 155.33, 141.97, 135.74, 130.12, 116.45, 116.03, 113.86, 42.36. 30.13, 29.37, 24.34, 21.13.

IR (CH_2Cl_2): ν_{max} 1735, 1714, 1604 cm^{-1}.

HRMS: Calcd. for $C_{15}H_{17}O_3$ (M + H), 245.1178; Found, 245.1159.

2g: viscous liquid

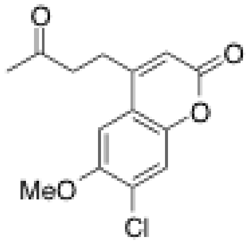

^{1}H NMR (500 MHz, $CDCl_3$):

δ ppm 7.52 (s, 1H), 7.14 (s, 1H), 6.13 (s, 1H), 2.95–2.92 (m, 2H), 2.81–2.78 (m, 2H), 2.38 (s, 3H), 2.17 (s, 3H)
^{13}C NMR (125 MHz, $CDCl_3$):

δ ppm 205.67, 160.37, 153.70, 151.94, 140.74, 130.28, 123.94, 191.21, 118.14, 113.79, 40.77, 30.07, 24.82, 20.50.

IR (CH_2Cl_2): 1731, 1701, 1610 cm^{-1}.

HRMS: Calcd. for $C_{14}H_{13}ClO_3Na$ (M + Na), 287.0451; Found, 287.0444

2h: yellow viscous liquid

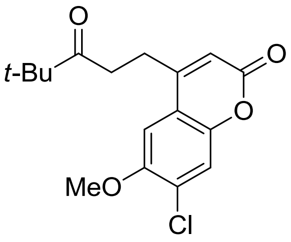

^{1}H NMR (500 MHz, $CDCl_3$):

δ ppm 7.54 (s, 1H), 6.78 (s, 1H), 6.05 (s, 1H), 3.89 (s, 3H), 2.91–2.88 (m, 2H) 2.84–2.81 (m, 2H), 1.11 (s, 9H).

^{13}C NMR (125 MHz, $CDCl_3$):

δ ppm 211.92. 159.17, 156.19, 153.17, 152.37, 123.53, 117.70, 111.46, 110.05, 99.17, 55.23, 42.76, 32.86, 24.98, 23.98.

IR (CH_2Cl_2): ν_{max} 1720, 1690, 1610 cm^{-1}.

HRMS: Calcd. for $C_{17}H_{20}ClO_4$ (M + H), 323.1050; Found, 323.1036.

3a: Colorless viscous liquid

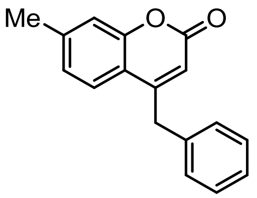

^{1}H NMR (400, $CDCl_3$):
δ 7. 53 (d, J = 8.0 Hz, 1H), 7.36–7.22 (m, 5H), 7.13 (s, 1H), 7.07 (d, J = 8.0 Hz, 1H), 6.07 (s, 1H), 4.09 (s, 2H), 2.43 (s, 3H) ppm.

^{13}C NMR (100 MHz, $CDCl_3$):

δ 161.25, 154.73, 153.78, 142.96, 136.13, 129.08, 128.97, 127.22, 125.47, 124.40, 117.34, 116.83, 114.53, 38.02, 21.60 ppm.

IR (CH_2Cl_2): ν_{max} 3028, 1712, 1620 cm^{-1}

HRMS: Calcd. for $C_{17}H_{14}O_2Na$ (M + Na) 273.0892; Found 273.0883.

3b: Colorless viscous liquid;

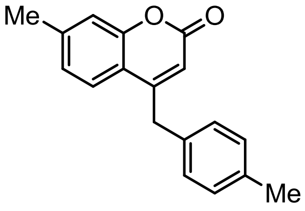

^{1}H NMR (400, $CDCl_3$):

δ 7.55 (d, J = 8.0 Hz, 1H), 7.17–7.07 (m, 6H), 6.09 (s, 1H), 4.06 (s, 2H), 2.45 (s, 3H), 2.36 (s, 3H) ppm.

^{13}C NMR (100 MHz, $CDCl_3$):

δ 161.32, 154.94, 153.81, 142.88, 136.86, 133.00, 129.64, 128.92, 125.42, 124.39, 117.35, 116.88, 114.46, 37.66, 21.60, 21.07 ppm.

IR (CH_2Cl_2): ν_{max} 3010, 1712, 1620 cm^{-1}

HRMS: Calcd. for $C_{18}H_{16}O_2Na$ (M + Na) 287.1048; Found 287.1025.

3c: Colorless solid

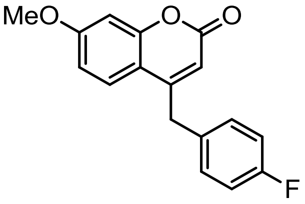

^{1}H NMR (400, $CDCl_3$):

δ 7.51 (d, J = 8.0 Hz, 1H), 7.21–7.17 (m, 2H), 7.04–7.00 (m, 2H), 6.83–6.81 (m, 2H), 5.95 (s. 1H), 4.05 (s, 2H), 3.87 (s, 3H) ppm.

^{13}C NMR (100 MHz, $CDCl_3$):

δ 162.65, 161.94 (d, $J_{C–F}$ = 244 Hz), 161.29, 155.53, 154.57, 131.84 (d, $J_{C–F}$ = 4 Hz), 130.57 (d, $J_{C–F}$ = 8 Hz), 125.55, 115.85 (d, $J_{C–F}$ = 22 Hz), 112.59, 112.42, 112.40, 101.03, 55.76, 37.29 ppm.

IR (CH_2Cl_2): ν_{max} 1711, 1611 cm^{-1}.

HRMS: Calcd. for $C_{17}H_{14}FO_3$ (M + H) 285.0927; Found 285.0902.

3d: Colorless viscous liquid

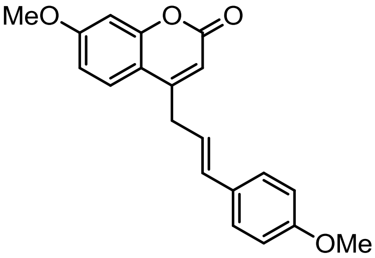

^{1}H NMR (400, $CDCl_3$):

δ 7.60 (d, J = 8 Hz, 1H), 7.32–7.28 (m, 2H), 6.88–6.85 (m, 4H), 6.51 (d, J = 16.0 Hz, 1H), 6.23–6.13 (m, 2H), 3.89 (s. 3H), 3.82 (s, 3H), 3.63 (d, J = 8 Hz, 2H) ppm.

^{13}C NMR (100 MHz, $CDCl_3$):

δ 162.62, 161.48, 159.34, 155.55, 154.66, 133.45, 129.35, 127.44, 125.51, 121.70, 114.04, 112.80, 112.40, 111.53, 101.02, 55.77, 55.32, 35.15 ppm.

IR (CH_2Cl_2): ν_{max} 2956, 1718, 1610 cm^{-1}

HRMS: Calcd. for $C_{20}H_{19}O_4$ (M + H) 323.1283; Found 323.1280.

3e: Colorless viscous liquid

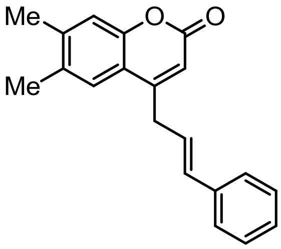

^{1}H NMR (400, $CDCl_3$):

δ 7.38–7.26 (m, 6H), 7.13 (s, 1H), 6.57 (d, *J* = 16.0 Hz, 1H), 6.37–6.29 (m, 2H), 3.66 (d, *J* = 8 Hz, 2H), 2.35 (s, 3H), 2.33 (s, 3H) ppm.

^{13}C NMR (100 MHz, $CDCl_3$):

δ 161.44, 154.28, 152.09, 141.84, 136.61, 132.98, 128.65, 127.80, 126.28, 124.54, 124.01, 117.74, 116.85, 113.56, 34.88, 20.21, 19.55 ppm.

IR (CH_2Cl_2): ν_{max} 3024, 1714, 1622 cm^{-1}.

HRMS: Calcd. for $C_{20}H_{19}O_2$ (M + H) 291.1385; Found 291.1357.

3f: Colorless viscous liquid

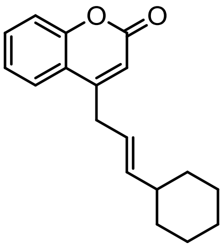

^{1}H NMR (400, $CDCl_3$):

δ 7.66 (d, *J* = 4.0 Hz, 1H), 7.56–7.52 (m, 1H), 7.37–7.28 (m, 2H), 6.32 (s, 1H), 5.66–5.61 (m, 1H), 5.55–5.49 (m, 1H), 3.48 (d, *J* = 8 Hz, 2H), 2.00 (bs, 1H), 1.75–1.65 (m, 4H), 1.33–1.05 (m, 6H) ppm.

^{13}C NMR (100 MHz, $CDCl_3$):

δ 161.14, 155.11, 153.65, 141.77, 131.62, 124.46, 124.12, 121.13, 119.34, 117.22, 114.28, 40.74, 34.79, 32.82, 26.09, 25.96 ppm.

IR (CH_2Cl_2): ν_{max} 1728, 1604, 1413 cm^{-1}.

HRMS: Calcd. for $C_{18}H_{21}O_2$ (M + H) 269.1542; Found 269.1519.

4a: yellow viscous liquid

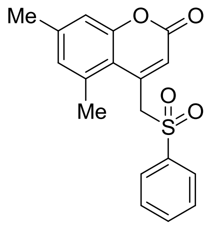

^{1}H NMR (500, $CDCl_3$):

δ ppm 7.74–7.72 (m, 2H), 7.64–7.61 (m, 1H), 7.51–7.48 (m, 2H), 6.95 (s, 1H), 6.84 (s, 1H), 5.89 (s, 1H), 4.65 (s, 2H), 2.73 (s, 3H), 2.30 (s, 3H).

^{13}C NMR (125 MHz, $CDCl_3$):

δ 159.22, 155.42, 143.31, 142.92, 137.39, 135.79, 134.70, 130.61, 129.54, 128.57, 120.26, 116.55, 114.95, 61.11, 24.66, 21.24 ppm.

IR (CH_2Cl_2): ν_{max} 1718, 1618, 1446 cm^{-1}.

HRMS: Calcd. for $C_{18}H_{16}O_4SNa$ (M + Na) 351.0667; Found 351.0642

4b: yellow viscous liquid

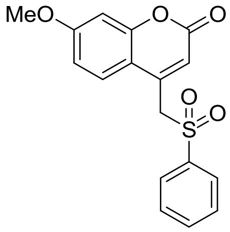

^{1}H NMR (500, $CDCl_3$):

δ ppm 7.76–7.74 (m, 2H), 7.61–7.58 (m, 1H), 7.48–7.43 (m, 3H), 6.77–6.72 (m, 2H), 5.78 (s, 1H), 4.39 (s, 1H), 3.80 (s, 3H).

^{13}C NMR (125 MHz, $CDCl_3$):

δ 163. 20, 159.88, 155.68, 142.73, 137.29, 134.67, 129.47, 128.55, 126.35, 116.13, 112.64, 111.54, 101.14, 58.60, 55.87ppm.

IR (CH_2Cl_2): ν_{max} 1710, 1610 cm^{-1}.

HRMS: Calcd. for $C_{17}H_{14}O_5SNa$ (M + Na) 353.0460; Found 353.0466

4c: yellow viscous liquid

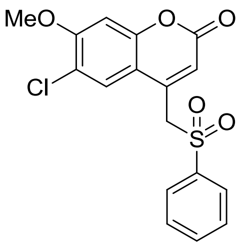

^{1}H NMR (500, $CDCl_3$ DMSO-D6):

δ 7.78 (d, *J* = 8 Hz, 2H), 7.62 (t, *J* = 7.5 Hz, 1H), 7.50–7.46 (m, 3H), 6.80 (s, 1H), 5.99 (s, 1H), 4.50 (s, 2H), 3.90 (s, 3H) ppm.

^{13}C NMR (125 MHz, $CDCl_3$):

δ 157.44, 156.16, 152.11, 140.38, 135.64, 132.88, 127.62, 126.63, 124.23, 117.33, 115.37, 109.95, 98.63, 56.29, 54.93 ppm.

IR (CH_2Cl_2): ν_{max} 1718, 1635 cm^{-1}.

HRMS: Calcd. for $C_{17}H_{14}ClO_5S$ (M + H) 365.0251; Found 365.0243

4d: yellow viscous liquid

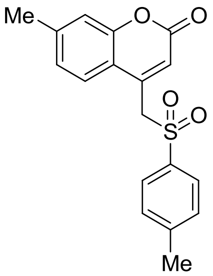

^{1}H NMR (500, $CDCl_3$):

δ 7.70 (d, *J* = 8.5 Hz, 2H), 7.55 (d, *J* = 8 Hz, 1H), 7.34 (d, *J* = 8.5 Hz, 2H), 7.15–7.11 (m, 2H), 5.93 (s, 1H), 4.48 (s, 2H), 2.46 (s, 3H), 2.45 (s, 3H); ppm.

^{13}C NMR (125 MHz, $CDCl_3$):

δ 159.78, 153.89, 145.94, 143.99, 142.91, 134.29, 130.09, 128.59, 125.70, 125.11, 118.36, 117.45, 115.63, 58.55, 21.74, 21.67.

IR (CH_2Cl_2): ν_{max} 1716, 1620 cm^{-1}.

HRMS: Calcd. for $C_{18}H_{17}O_4S$ (M + H) 329.0848; Found 329.0862

4e: yellow viscous liquid

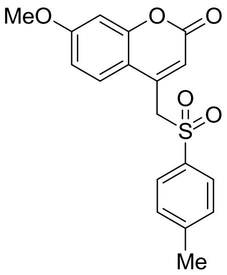

^{1}H NMR (500, $CDCl_3$):

δ 7.61 (d, *J* = 8.5 Hz, 2H), 7.48 (d, *J* = 8.5 Hz, 1H), 7.25 (d, *J* = 8.5Hz, 2H), 6.78–6.74 (m, 2H), 5.74 (s, 1H), 4.36 (s, 2H), 3.80 (s, 3H), 2.36 (s, 3H) ppm.

^{13}C NMR (125 MHz, $CDCl_3$):

δ 161.07, 157.82, 153.56, 143.78, 140.82, 132.19, 127.94, 126.44, 124.38, 113.90, 110.46, 109.49, 99.02, 56.60, 53.74, 19.60 ppm.

IR (CH_2Cl_2): ν_{max} 1716, 1608 cm^{-1}.

HRMS: Calcd. for $C_{18}H_{16}O_5SNa$ (M + Na) 367.0616; Found 367.0604

5a: yellow solid

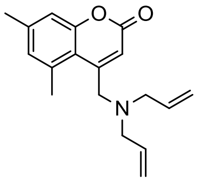

^{1}H NMR ($CDCl_3$):

δ 6.93 (s, 1H), 6.79 (s, 1H), 6.74 (s, 1H), 5.81–5.74 (m, 2H), 5.15–5.07 (m, 4H), 3.75 (s, 2H), 3.10–3.08 (m, 4H), 2.58 (s, 3H), 2.28 (s, 3H) ppm.

^{13}C NMR (125 MHz, $CDCl_3$):

δ 161.98, 156.37, 155.98, 142.23, 136.56, 135.60, 130.28, 118.66, 116.82, 116.76, 114.61, 57.76, 57.60, 25.13, 21.77 ppm.

IR (CH_2Cl_2): ν_{max} 3018, 1724 cm^{-1}

HRMS: Calcd. for $C_{18}H2_2NO_2$ (M + H) 284.1651; Found 284.1627.

5b: yellow liquid

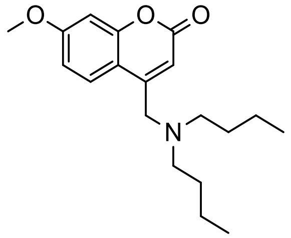

^{1}H NMR ($CDCl_3$):

δ ppm 7.67 (d, *J* = 8.5 Hz, 1H), 6.76–6.74 (m, 2H), 6.39 (s, 1H), 3.79 (s, 3H), 3.57 (s, 2H), 2.39 (t, *J* = 7 Hz, 4H), 1.38–1.35 (m, 4H), 1.24–1.29 (m, 4H), 0.81 (t, *J* = 7.5 Hz, 6H).

^{13}C NMR (125 MHz, $CDCl_3$):

δ 160.38, 159.78, 153.50, 152.46, 123.65, 110.59, 110.01, 109.41, 98.74, 53.85, 53.69, 52.36, 27.25, 18.57,12.04 ppm

IR (CH_2Cl_2): ν_{max} 3018, 1724, 1612 cm^{-1}

HRMS: Calcd. for $C_{19}H_{28}NO_3$ (M + H) 318.2069, Found 318.2058.

5c: yellow liquid

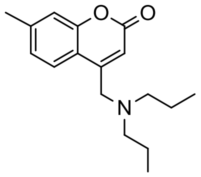

^{1}H NMR ($CDCl_3$):

δ 7.61 (d, J = 8 Hz, 1H), 7.06 (s, 1H), 7.00–6.98 (m, 1H), 6.50 (s, 1H), 3.60 (s, 3H), 2.38–2.35 (m, 7H), 1.44–1.39 (m, 4H), 0.79 (t, J = 7.5 Hz, 6H) ppm.

^{13}C NMR (125 MHz, $CDCl_3$):

δ 161.66, 154.30, 153.77, 142.57, 125.10, 124.18, 117.14, 116.57, 113.37, 56.63, 55.67, 21.60, 20.31, 11.85 ppm.

IR (CH_2Cl_2): ν_{max} 2958, 1724 cm^{-1}.

HRMS: Calcd. for $C_{17}H_{24}NO_2$ (M + H) 274.1807; Found 274.1875.

5d: Yellow viscous liquid

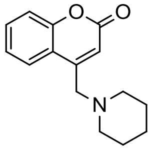

^{1}H NMR (500, $CDCl_3$):

δ 7.76 (dd, *J* = 8, 1.5 Hz, 1H), 7.45–7.41 (m, 1H), 7.26–7.17 (m, 2H), 6.47 (s, 1H), 3.51 (s, 1H), 2.40 (bs, 4H), 1.55–1.50 (m, 4H), 1.39–1.38 (m, 2H) ppm.

^{13}C NMR (125 MHz, $CDCl_3$):

δ 161.19, 153.74, 152.67, 131.50, 124.77, 124.01, 119.04, 116.98, 114.59, 59.63, 55.02, 26.01, 24.09 ppm.

IR (CH_2Cl_2): ν_{max} 3010, 1730 cm^{-1}

HRMS: Calcd. for $C_{15}H_{18}NO_2$ (M + H) 244.1338; Found 244.1308.

5e: yellow liquid

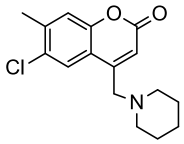

^{1}H NMR ($CDCl_3$):

δ 7.76 (s, 1H), 7.13 (s, 1H), 6.44 (s, 1H), 3.47 (s, 2H), 2.39–2.38 (m, 7H), 1.56–1.52 (m, 4H), 1.40–139 (m, 2H) ppm.

^{13}C NMR (125 MHz, $CDCl_3$):

δ 158.67, 149.97, 149.54, 138.17, 127.74, 122.57, 116.67, 115.95, 112.49, 57.44, 52.81, 23.81, 21.89, 18.39 ppm.

IR (CH_2Cl_2): ν_{max} 1733, 1521 cm^{-1}.

HRMS: Calcd. for $C_{16}H_{19}ClNO_2$ (M + H) 292.1104; Found 292.1070.

5f: yellow viscous liquid

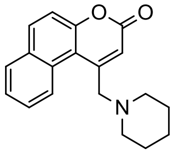

^{1}H NMR (400, $CDCl_3$):

δ ppm 8.68 (d, *J* = 8.4 Hz, 1H), 7.98 (d, *J* = 8.8, 1H), 7.92 (dd, *J* = 8, 1.6 Hz, 1H), 7.68–7.64 (m, 1H), 7.59–7.55 (m, 1H), 7.49 (d, *J* = 8.8 Hz, 1H), 6.86 (s, 1H), 3.93 (s, 2H), 2.60 (m, 4H), 1.66 (m, 4H), 1.53 (m, 2H).

^{13}C NMR (125 MHz, $CDCl_3$):

δ ppm 160.92, 154.89, 154.19, 133.49, 131.28, 129.79, 129.33, 127.62, 126.65, 125.46, 117.79, 116.20, 114.62, 63.71, 54.68, 26.01, 24.19.

IR (CH_2Cl_2): ν_{max} 3018, 1730 cm^{-1}.

HRMS: Calcd. for $C_{19}H_{20}NO_2$ (M + H) 294.1494; Found 294.1482.

5g: yellow solid;

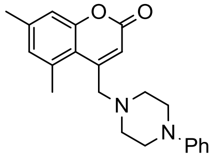

^{1}H NMR (400, $CDCl_3$):

δ 7.31–7.27 (m, 2H), 7.05 (s, 1H), 6.95–6.88 (m, 4H), 6.56 (s, 1H), 3.74 (s, 2H), 3.23 (t, *J* = 4.8 Hz, 4H), 2.79 (s, 3H), 2.72 (t, *J* = 4.8 Hz, 4H), 2.39 (s, 3H) ppm.

^{13}C NMR (100 MHz, $CDCl_3$) δ 160.97, 155.61, 153.12, 151.13, 141.89, 136.61, 129.78, 129.16, 119.90, 116.15, 115.99, 115.84 ppm.

IR (CH_2Cl_2): ν_{max} 2821, 1722 cm^{-1}.

HRMS: Calcd. for $C_{22}H_{25}N_2O_2$ (M + H) 349.1916; Found 349.1908.

5h: yellow solid liquid

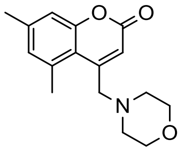

^{1}H NMR (500, $CDCl_3$):

δ 6.93 (s, 1H), 6.83 (s, 1H), 6.43 (s, 1H), 3.64 (t, *J* = 4.5 Hz, 4H), 3.59 (s, 2H), 2.67 (s, 3H), 2.46 (s, 4H), 2.29 (s, 3H) ppm.

^{13}C NMR (125 MHz, $CDCl_3$):

δ 160.82, 155.58, 152.72, 141.89, 136.51, 129.74, 116.08, 115.90, 115.85, 66.87, 62.56, 53.63, 23.42, 21.14 ppm.

IR (CH_2Cl_2): ν_{max} 3018, 1724, 1618 cm^{-1}.

HRMS: Calcd. for $C_{16}H_{20}N0_3$ (M + H) 274.1443; Found 274.1422.

5i: yellow viscous liquid

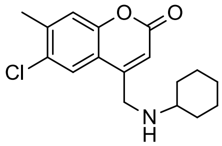

^{1}H NMR (400, $CDCl_3$):

δ 7.67 (s, 1H), 7.22 (s, 1H), 6.58 (s, 1H), 3.96 (s, 2H), 2.57–2.51 (m, 1H), 2.47 (s, 3H), 1.98–1.95 (m, 2H), 1.79–1.76 (m, 2H), 1.35–1.12 (m, 6H) ppm.

^{13}C NMR (100 MHz, $CDCl_3$):

δ 160.86, 153.66, 152.02, 140.32, 130.01, 123.92, 119.02, 117.79, 113.36, 56.72, 46.55, 33.65, 26.04, 24.92, 20.53 ppm.

IR (CH_2Cl_2): ν_{max} 3681, 3018, 1521 cm^{-1}.

HRMS: Calcd. for $C_{17}H_{21}ClNO_2$ (M + H) 306.1261; Found 306.1241

5j: yellow viscous liquid

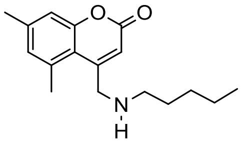

^{1}H NMR ($CDCl_3$):

δ 7.03 (s, 1H), 6.90 (s, 1H), 6.55 (s, 1H), 4.03 (s, 2H), 2.73–2.69 (m, 4H), 2.38 (s, 3H), 1.55 (t, J = 9 Hz, 2H), 1.36–1.33 (m, 4H), 0.92 (m, 3H) ppm.

^{13}C NMR (125 MHz, $CDCl_3$):

δ 161.18, 155.93, 155.43, 141.68, 136.08, 129.71, 116.14, 115.88, 113.51, 53.42, 49.87, 29.87, 29.48, 23.74, 22.61, 21.14, 14.06 ppm.

IR (CH_2Cl_2): ν_{max} 1724, 1691 cm^{-1}.

HRMS: Calcd. for $C_{17}H_{24}NO_2$ (M + H) 274.1807 Found 274.1766.

5k: yellow viscous liquid;

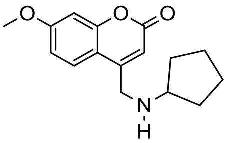

^{1}H NMR (400, $CDCl_3$):

7.60 (d, J = 8 Hz, 1H), 6.87–6.82 (m, 2H), 6.41 (s, 1H), 3.91 (s, 2H), 3.88 (s, 3H), 3.20–3.17 (m, 1H), 1.91–1.85 (m, 2H), 1.76–1.70 (m, 2H), 1.61–1.55 (m, 2H), 1.45–1.38 (m, 2H) ppm.

^{13}C NMR (125 MHz, $CDCl_3$):

δ 160.50, 159.61, 153.55, 152.34, 123.08, 110.35, 110.23,108.40, 98.96, 57.79, 53.76, 46.40, 31.19, 21.97 ppm.

IR (CH_2Cl_2): ν_{max} 17,20, 1612 cm^{-1}.

HRMS: Calcd. for $C_{16}H_{20}NO_3$ (M + H) 274.1443; Found 274.1396.

Library experimental/representative spectral data and NMR reprints

Experimental procedure

In the appropriate reactor position in the Chemspeed SLT1000 (4C × 16A = 64 member library) was added 96.7 mg of potassium carbonate (0.700 mmol), 2.50 mL of a 0.140 M (0.350 mmol) solution in dioxane of the coumarin (C), 3.30 mL of a 0.0105 M (0.0105 mmol) solution in dioxane of the tetrakis(triphenylphosphine)palladium and 0.66 mL of a 0.800 M (0.525 mmol) solution in dioxane of the amine (A), and the reaction mixture was vortex shaken at 90 °C for 16 hours. The reaction mixtures were concentrated, ethyl acetate (2 mL) was added to the residues and the resulting solutions were gravity filtered through SPE-Si (400 mg) into the appropriate CCT tubes. The process was repeated twice (2 × 2 mL), the filtrates were concentrated and the resulting residues were purified by LC–MS.

^{1}H, ^{13}C NMR spectra were recorded on an Avance AV-III 500 with a dual carbon/proton (CPDUL) cryoprobe operating at 500 MHz and 125 MHz respectively. Infrared (IR) spectra were obtained using a Perkin-Elmer Spectrum 100 FT–IR spectrometer with a UATR application.

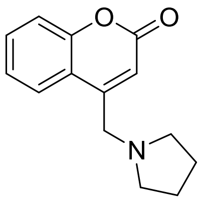

C1A5

4-(Pyrrolidin-1-ylmethyl)-2*H*-chromen-2-one: ^{1}H NMR (500 MHz, $CDCl_3$) δ 7.82–7.80 (m, 1H), 7.52–7.49 (m, 1H), 7.34–7.26 (m, 2H), 6.54 (d, *J* = 1 Hz, 1H), 3.79 (d, *J* = 1 Hz, 2H), 2.65–2.62 (m, 4H), 1.84–1.82 (m, 4H) ppm; ^{13}C NMR (125 MHz, $CDCl_3$) δ 158.83, 151.32, 150.55, 129.22, 122.31, 121.76, 116.47, 114.66, 112.10, 54.10, 52.09, 21.32 ppm; IR (CH_2Cl_2): ν 1717, 1617 cm^{-1}; HRMS: Calcd for $C_{14}H_{16}NO_2$ (M + H) 230.1136; found: 230.1179.

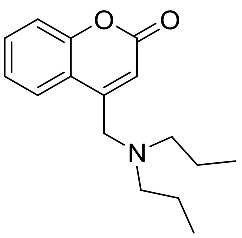

C1A7

4-((Dipropylamino)methyl)-2*H*-chromen-2-one: ^{1}H NMR (500 MHz, $CDCl_3$) δ 7.83–7.81 (m, 1H), 7.52–7.49 (m, 1H), 7.33–7.25 (m, 2H), 6.66 (t, *J* = 1Hz, 1H), 3.72 (d, *J* = 1 Hz, 2H), 2.46 (t, *J* = 7.5 Hz, 4H), 1.54–1.46 (m, 4H), 0.88 (t, *J* = 7.5 Hz, 6H) ppm; ^{13}C NMR (125 MHz, $CDCl_3$) δ 160.97, 153.90, 153.25, 131.11, 124.08, 123.58, 118.58, 116.60, 114.02, 56.24, 55.21, 19.91, 11.46 ppm; IR (CH_2Cl_2): ν 1724, 1623, 1550 cm^{-1}; HRMS: Calcd for $C_{16}H_{22}NO_2$ (M + H) 260.1606; found 260.1649.

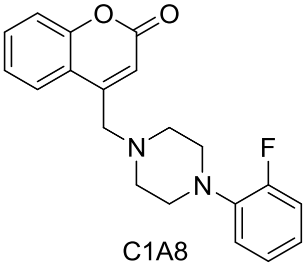

C1A8

4-((4-(2-Fluorophenyl)piperazin-1-yl)methyl)-2*H*-chromen-2-one: ^{1}H NMR (500 MHz, $CDCl_3$) δ 7.85–7.83 (m, 1H), 7.55–7.52 (m,1H), 7.36–7.34 (m, 1H), 7.31–7.17 (m, 1H), 7.05–6.93 (m, 4H), 6.60 (s, 1H), 3.72 (d, *J* = 5 Hz, 2H), 3.15–3.13 (m, 4H), 2.76–2.74 (m, 4H) ppm; ^{13}C NMR (125 MHz, $CDCl_3$) δ 161.03, 155.72 (d, J_{CF} = 244 Hz), 153.82, 151.86, 139.9 (d, J_{CF} = 8.7 Hz), 131.75, 124.75, 124.52 (d, J_{CF} = 2.5 Hz), 124.16, 122.64 (d, J_{CF} = 7.5 Hz), 118.93 (d, J_{CF} = 17.5 Hz), 118.86, 117.12, 116.15 (d, J_{CF} = 20 Hz), 114.94, 58.84, 53.60, 50.55, 50.52; IR (CH_2Cl_2): ν 1719, 1624, 1605 cm^{-1}; HRMS: Calcd for $C_{20}H_{20}FN_2O_2$ (M + H) 339.1464; found 339.1526.

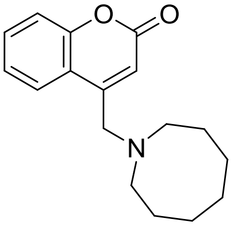

C1A14

4-(Azocan-1-ylmethyl)-2*H*-chromen-2-one: ^{1}H NMR (500 MHz, $CDCl_3$) δ 7.89–7.87 (m, 1H), 7.52–7.49 (m, 1H), 7.34–7.27 (m, 2H), 6.50 (s, 1H), 3.75 (d, *J* = 5 Hz, 2H), 2.67–2.64 (m, 4H), 1.60–1.55 (m, 10H) ppm; ^{13}C NMR (125 MHz, $CDCl_3$) δ 160.19, 152.76, 152.74, 130.55, 124.31, 122.91, 118.04, 115.94, 113.98, 59.33, 53.95, 26.77, 26.36, 24.84 ppm; IR (CH_2Cl_2): ν 1720, 1623, 1605, cm^{-1}; HRMS: Calcd for $C_{17}H_{22}NO_2$ (M + H) 272.1606; found: 272.1658.

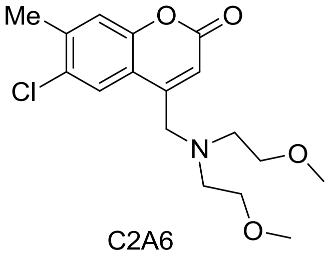

C2A6

4-((bis(2-Methoxyethyl)amino)methyl)-6-chloro-7-methyl-2*H*-chromen-2-one: ^{1}H NMR (500 MHz, $CDCl_3$) δ 7.94 (s, 1H), 7.19 (d, *J* = 1 Hz, 1H), 6.59 (s, 1H), 3.89 (d, *J* = 5 Hz, 2H), 3.49 (t, *J* = 5 Hz, 4H), 3.33 (s, 6H), 2.82 (t, *J* = 5 Hz, 4H), 2.45 (s, 3H) ppm; ^{13}C NMR (125 MHz, $CDCl_3$) δ 160.90, 153.03, 152.06, 140.21, 129.86, 125.01, 118.69, 117.98, 114.70, 71.39, 58.82, 56.36, 54.42, 20.52 ppm; IR (CH_2Cl_2): ν 1731, 1616 cm^{-1}; HRMS: Calcd for $C_{17}H_{23}ClNO_4$ (M + H) 340.1271; found: 340.1326.

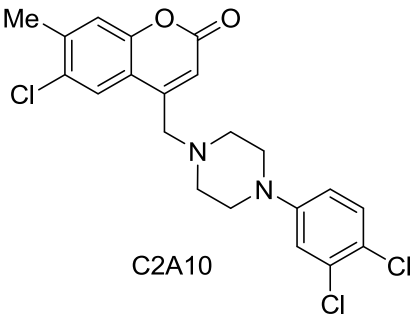

C2A10

6-Chloro-4-((4-(3,4-dichlorophenyl)piperazin-1-yl)methyl)-7-methyl-2*H*-chromen-2 one: ^{1}H NMR (500 MHz, $CDCl_3$) δ 7.81 (s, 1H), 7.28–7.26 (m, 1H), 7.22 (s, 1H), 6.96 (d, *J* = 3 Hz, 1H), 6.75–6.73 (m, 1H), 6.53 (s, 1H), 3.65 (s, 2H), 3.21 (t, *J* = 5 Hz, 4H), 2.69 (t, *J* = 5 Hz, 4H), 2.46 (s, 3H) ppm; ^{13}C NMR (125 MHz, $CDCl_3$) δ 160.58, 152.17, 150.63, 150.47, 140.70, 132.82, 130.48, 130.07, 124.62, 122.44, 118.97, 117.78, 117.41, 115.45, 115.00, 58.77, 53.11, 48.74, 20.60 ppm ; IR (CH_2Cl_2): ν 1728, 1616, 1592 cm^{-1}; HRMS: Calcd for $C_{21}H_{20}C_{l3}N_2O_2$ (M + H) 437.0546; found: 437.0567.

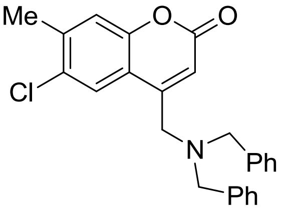

C2A12

6-Chloro-4-((dibenzylamino)methyl)-7-methyl-2*H*-chromen-2-one: ^{1}H NMR (500 MHz, $CDCl_3$) δ 7.71 (s, 1H), 7.34–7.24 (m, 10H), 7.11 (s, 1H), 6.61 (s, 1H), 3.61–3.60 (s, 6H), 2.40 (s, 3H) ppm; ^{13}C NMR (125 MHz, $CDCl_3$) δ 159.63, 151.34, 151.07, 139.51, 137.19, 128.92, 128.05, 127.65, 126.57, 124.10, 117.77, 116.87, 114.24, 58.01, 53.88, 19.56 ppm ; IR (CH_2Cl_2): ν 1731, 1617, 1548 cm^{-1}; HRMS: Calcd for $C_{25}H_{23}ClNO_2$ (M + H) 404.1373; found: 404.1428 .

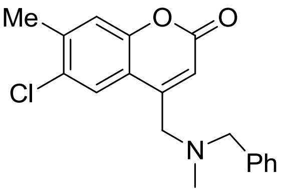

C2A13

4-((Benzyl(methyl)amino)methyl)-6-chloro-7-methyl-2*H*-chromen-2-one: ^{1}H NMR (500 MHz, $CDCl_3$) δ 7.75 (s, 1H), 7.34–7.26 (m, 5H), 7.15 (s, 1H), 6.52 (s, 1H), 3.62 (s, 2H), 3.54 (s, 2H), 2.41 (s, 3H), 2.26 (s, 3H) ppm; ^{13}C NMR (125 MHz, $CDCl_3$) δ 158.77, 150.21, 150.08, 138.53, 136.26, 128.03, 127.15, 126.69,125.65, 123.15, 116.85, 115.98, 113.22, 60.92, 55.97, 40.91, 18.64 ppm ; IR (CH_2Cl_2): ν 1728, 1616 cm^{-1}; HRMS: Calcd for $C_{19}H_{19}ClNO_2$ (M + H) 328.1060; found: 328.1118.

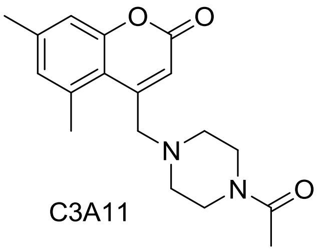

C3A11

4-((4-Acetylpiperazin-1-yl)methyl)-5,7-dimethyl-2*H*-chromen-2-one: ^{1}H NMR (500 MHz, $CDCl_3$) δ 7.01 (s, 1H), 6.91 (s, 1H), 6.51 (s, 1H), 3.71 (s, 2H), 3.65 (t, *J* = 5 Hz, 2H), 3.49 (t, *J* = 5 Hz, 2H), 2.74 (s, 3H), 2.53 (q, *J* = 5 Hz, 4H), 2.38 (s, 3H), 2.10 (s, 3H) ppm; ^{13}C NMR (125 MHz, $CDCl_3$) δ 168.99, 160.77, 155.53, 152.76, 142.01, 136.38, 129.80, 116.08, 115.79, 115.62, 61.94, 53.07, 52.99, 46.15, 41.32, 23.52, 21.33, 21.15 ppm; IR (CH_2Cl_2): ν 1728, 1638, 1617 cm^{-1}; HRMS: Calcd for $C_{18}H_{23}N_2O_3$ (M + H) 315.1664; found: 315.1728

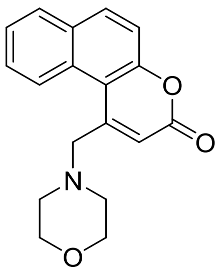

C4A1

1-(Morpholinomethyl)-3*H*-benzo[*f*]chromen-3-one: ^{1}H NMR (500 MHz, $CDCl_3$) δ 8.65 (d, *J* = 10 Hz, 1H), 7.95 (d, *J* = 5 Hz, 1H), 7.90 (d, *J* = 10 Hz, 1H), 7.66–7.62 (m, 1H), 7.57–7.54 (m, 1H), 7.44 (d, *J* = 10 Hz, 2H), 6.82 (s, 1H), 3.96 (s, 2H), 3.78–3.76 (m, 4H), 2.66–2.65 (m, 4H) ppm; ^{13}C NMR (125 MHz, $CDCl_3$) 160.64, 154.93, 152.98, 133.73, 131.28, 129.59, 129.48, 127.77, 126.25, 125.59, 117.73, 116.24, 114.28, 66.89, 63.13, 53.59 ppm; IR (CH_2Cl_2): ν 1724, 1618, 1617 cm^{-1}; HRMS: Calcd for $C_{18}H_{18}NO_3$ (M + H) 296.1242; found 296.1292.

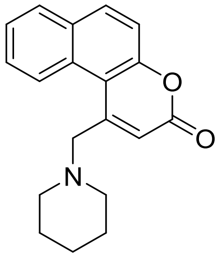

C4A2

1-(Piperidin-1-ylmethyl)-3*H*-benzo[*f*]chromen-3-one: ^{1}H NMR (500 MHz, $CDCl_3$) δ 8.62 (d, *J* = 10 Hz, 1H), 7.93 (d, *J* = 10 Hz, 1H), 7.89–7.87 (m, 1H), 7.64–7.61 (m, 1H), 7.55–7.52 (m, 1H), 7.44 (d, *J* = 10 Hz, 1H), 6.82 (s, 1H), 3.88 (s, 2H), 2.56 (bs, 4H), 1.65–1.61 (m, 4H), 1.50–1.47 (m, 2H) ppm; ^{13}C NMR (125 M Hz, $CDCl_3$) δ 160.08, 154.01, 153.38, 132.65, 130.42, 128.92, 128.49, 126.81, 125.79, 124.64, 116.89, 115.28, 113.74, 62.84, 53.84, 25.19, 23.36 ppm; IR (CH_2Cl_2): ν 1726, 1624, 1550 cm^{-1}; HRMS: Calcd for $C_{19}H_{20}NO_2$ (M + H) 294.1449; found: 294.1492.

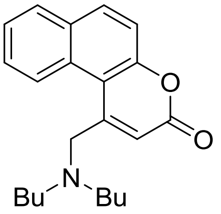

C4A3

1-((Dibutylamino)methyl)-3*H*-benzo[*f*]chromen-3-one: ^{1}H NMR (500 MHz, $CDCl_3$) δ 8.54 (d, *J* = 10 Hz, 1H), 7.94 (d, *J* = 5 Hz, 1H), 7.91–7.89 (m, 1H), 7.65–7.63 (m, 1H), 7.56–7.53 (m, 1H), 7.46 (d, *J* = 5 Hz, 1H), 7.09 (s, 1H), 4.09 (s, 2H), 2.56 (t, *J* = 5 Hz, 4H), 1.53–1.47 (m, 4H), 1.37–1.29 (m, 4H), 0.90 (t, *J* = 5 Hz, 6H) ppm; ^{13}C NMR (125 MHz, $CDCl_3$) δ 160.18, 155.60, 153.64, 132.34, 130.28, 128.73, 128.46, 126.59, 125.38, 124.40, 116.81, 114.28, 113.51, 59.10, 53.20, 28.36, 19.63, 13.08 ppm; IR (CH_2Cl_2): ν 1722, 1623, 1549 cm^{-1}; HRMS: Calcd for $C_{22}H_{28}NO_2$ (M + H) 338.2075; found: 338.2119.

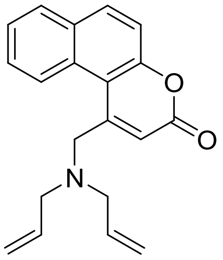

C4A4

1-((Diallylamino)methyl)-3*H*-benzo[*f*]chromen-3-one:^{1}H NMR (500 MHz, $CDCl_3$) δ 8.44 (d, *J* = 10 Hz, 1H), 7.95 (d, *J* = 10 Hz, 1H), 7.91–7.89 (m, 1H), 7.63–7.61 (m, 1H), 7.56–7.53 (m, 1H), 7.47 (d, *J* = 10 Hz, 1H), 7.08 (t, *J* = 1Hz, 1H), 5.96–5.88 (m, 2H), 5.28–5.18 (m, 4H), 4.14 (d, *J* = 1 Hz, 2H), 3.23 (d, *J* = 5 Hz, 4H) ppm; ^{13}C NMR (125 MHz, $CDCl_3$) δ 161.06, 155.78, 154.73, 134.88, 133.49, 131.31, 129.65, 129.53, 127.65, 126.19, 125.45, 118.24, 117.83, 115.18, 114.37, 58.22, 56.96 ppm; IR (CH_2Cl_2): ν 1727, 1551 cm^{-1}; HRMS: Calcd for $C_{20}H_{20}NO_2$ (M + H) 306.1449; found: 306.1501.

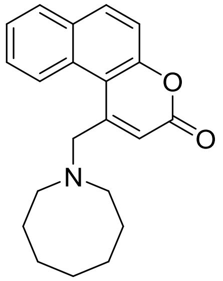

C4A14

1-(Azocan-1-ylmethyl)-3*H*-benzo[*f*]chromen-3-one: ^{1}H NMR (500 MHz, $CDCl_3$) δ 8.73 (d, *J* = 10 Hz, 1H), 7.96 (d, *J* = 10 Hz, 1H), 7.90–7.88 (m, 1H), 7.66–7.63 (m, 1H), 7.56–7.53 (m, 1H), 7.46 (d, *J* = 10 Hz, 1H), 6.73 (s, 1H), 4.03 (d, *J* = 1 Hz, 2H), 2.74 (t, *J* = 5 Hz, 4H), 1.55–1.52 (m, 10H) ppm; ^{13}C NMR (125 MHz, $CDCl_3$) δ 160.86, 155.26, 154.83, 133.56, 131.21, 129.76, 129.21, 127.59, 127.15, 125.53, 117.76, 116.98, 114.69, 64.11, 54.32, 27.94, 27.20, 25.83 ppm; IR (CH_2Cl_2): ν 1720, 1623, 1549 cm^{-1}; HRMS: Calcd for $C_{21}H_{24}NO_2$ (M + H) 322.1762; found: 322.1790.

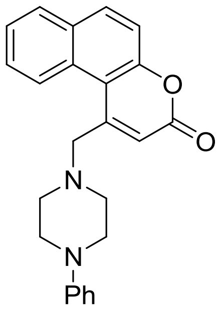

C4A16

1-((4-Phenylpiperazin-1-yl)methyl)-3*H*-benzo[*f*]chromen-3-one: ^{1}H NMR (500 MHz, $CDCl_3$) δ 8.61 (d, *J* = 10 Hz, 1H), 7.97 (d, *J* = 10 Hz, 1H), 7.91–7.89 (m, 1H), 7.66–7.63 (m, 1H), 7.57–7.54 (m, 1H), 7.47 (d, *J* = 10 Hz, 1H), 7.28–7.25 (m, 2H), 6.94–6.93 (m, 2H), 6.88–6.85 (m, 2H), 4.02 (s, 2H), 3.25 (t, *J* = 5 Hz, 4H), 2.82 (t, *J* = 5 Hz, 4H) ppm; ^{13}C NMR (125 MHz, $CDCl_3$) δ 160.74, 154.99, 153.30, 151.13, 133.75, 131.32, 129.67, 129.48, 129.18, 127.78, 126.39, 125.61, 119.98, 117.79, 116.36, 116.21, 114.40, 62.83, 53.23, 49.22 ppm; IR (CH_2Cl_2): ν 1720, 1622, 1598 cm^{-1}; HRMS: Calcd for $C_{24}H_{23}N_2O_2$ (M + H) 371.1715; found: 371.1732.

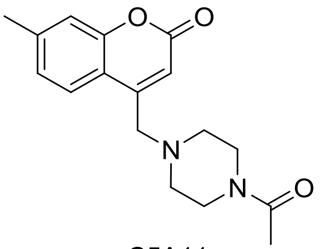

C5A11

4-((4-Acetylpiperazin-1-yl)methyl)-7-methyl-2*H*-chromen-2-one: ^{1}H NMR (500 MHz, $CDCl_3$) δ 7.68 (d, *J* = 10 Hz, 1H), 7.14–7.10 (m, 2H), 6.48 (s, 1H), 3.67–3.65 (m, 4H), 3.51 (t, *J* = 5 Hz, 2H), 2.54 (q, *J* = 5 Hz, 4H), 2.45 (s, 3H), 2.11 (s, 3H) ppm; ^{13}C NMR (125 MHz, $CDCl_3$) δ 168.97, 161.15, 153.87, 151.47, 143.06, 125.37, 124.31, 117.22, 116.21, 113.78, 58.65, 53.35, 53.17, 46.20, 41.36, 21.63, 21.35 ppm; IR (CH_2Cl_2): ν 1717, 1618, 1557 cm^{-1}; HRMS: Calcd for $C_{17}H_{21}N_2O_3$ (M + H) 301.1507; found: 301.1545.

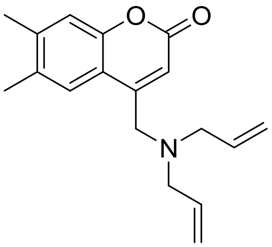

C6A4

4-((Diallylamino)methyl)-6,7-dimethyl-2*H*-chromen-2-one: ^{1}H NMR (500 MHz, $CDCl_3$) δ 7.47 (s, 1H), 7.09 (s, 1H), 6.56 (s, *J* = 1 Hz, 1H), 5.89–5.84 (m, 2H), 5.25–5.17 (m, 4H), 3.69 (d, *J* = 1 Hz, 2H), 3.16 (d, *J* = 5 Hz, 4H), 2.33 (s, 3H), 2.31 (s, 3H) ppm; ^{13}C NMR (125 MHz, $CDCl_3$) δ 160.48, 152.26, 150.85, 140.27, 133.76, 131.41, 123.23, 116.94, 116.26, 115.27, 112.07, 56.0, 52.30, 18.95, 18.25 ppm; IR (CH_2Cl_2): ν 1713, 1623, 1557 cm^{-1}; HRMS: Calcd for $C_{18}H_{22}NO_2$ (M + H) 284.1606; found: 284.1645.

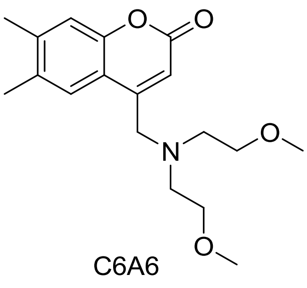

C6A6

4-((bis(2-Methoxyethyl)amino)methyl)-6,7-dimethyl-2*H*-chromen-2-one: ^{1}H NMR (500 MHz, $CDCl_3$) δ 7.54 (s, 1H), 7.10 (s, 1H), 6.60 (s, 1H), 3.91 (d, *J* = 1 Hz, 2H), 3.51 (t, *J* = 5 Hz, 4H), 3.32 (s, 6H), 2.84 (t, *J* = 5 Hz, 4H), 2.33 (s, 3H), 2.31 (s, 3H) ppm; ^{13}C NMR (125 MHz, $CDCl_3$) δ 161.83, 153.97, 152.07, 141.40, 132.60, 124.58, 117.43, 116.51, 113.18, 71.44, 58.81, 56.15, 54.63, 20.17, 19.43 ppm; IR (CH_2Cl_2): ν 1717, 1623, 1557 cm^{-1}; HRMS: Calcd for $C_{18}H_{26}NO_4$ (M + H) 320.1817 ; found: 320.1857.

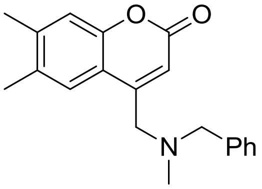

C6A13

4-((Benzyl(methyl)amino)methyl)-6,7-dimethyl-2*H*-chromen-2-one: ^{1}H NMR (500 MHz, $CDCl_3$) δ 7.37–7.28 (m, 6H), 7.07 (s, 1H), 6.51 (s, 1H), 3.64 (s, 2H), 3.58 (s, 2H), 2.31 (s, 3H), 2.28 (s, 3H), 2.26 (s, 3H) ppm; ^{13}C NMR (125 MHz, $CDCl_3$) δ 160.41, 151.63, 150.97, 140.39, 137.22, 131.45, 127.83, 127.26, 126.22, 123.74, 116.22, 115.29, 112.72, 61.65, 56.43, 41.70, 18.97, 18.15 ppm; IR (CH_2Cl_2): ν 1714, 1623, 1557 cm^{-1}; HRMS: Calcd for $C_{20}H_{22}NO_2$ (M + H) 308.1606; found: 308.1641.

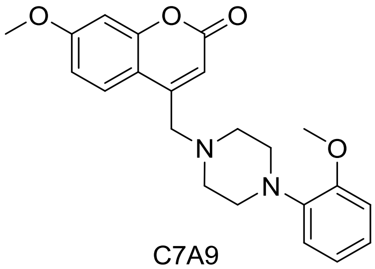

C7A9

7-Methoxy-4-((4-(2-methoxyphenyl)piperazin-1-yl)methyl)-2*H*-chromen-2-one: ^{1}H NMR (500 MHz, $CDCl_3$) δ 7.79 (d, *J* = 10 Hz, 1H), 7.02–6.82 (m, 6H), 6.41 (s, 1H) 3.87 (s, 3H), 3.86 (s, 3H), 3.66 (s, 2H), 3.11 (bs, 4H), 2.74 (bs, 4H) ppm; ^{13}C NMR (125 MHz, $CDCl_3$) δ 162.55, 161.51, 155.65, 152.24, 141.10, 125.97, 123.07, 121.00, 118.24, 112.48, 112.23, 111.82, 111.17, 100.80, 59.22, 55.75, 55.38, 53.74, 50.65 ppm; IR (CH_2Cl_2): ν 1717, 1610, 1557 cm^{-1}; HRMS: Calcd for $C_{22}H_{25}N_2O_4$ (M + H) 381.1770; found: 381.1804 .

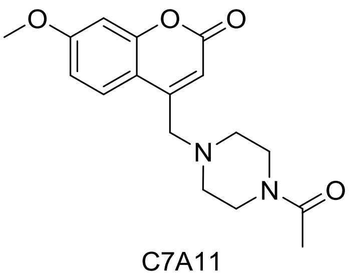

C7A11

4-((4-Acetylpiperazin-1-yl)methyl)-7-methoxy-2*H*-chromen-2-one: ^{1}H NMR (500 MHz, $CDCl_3$) δ 7.73 (d, *J* = 10 Hz, 1H), 6.87–6.82 (m, 2H), 6.37 (s, 1H), 3.88 (s, 3H), 3.67–3.63 (m, 4H), 3.50 (t, *J* = 5 Hz, 2H), 2.55–2.52 (m, 4H), 2.11 (s, 3H) ppm; ^{13}C NMR (125 MHz, $CDCl_3$) δ 168.96, 162.64, 161.29, 155.63, 151.59, 125.75, 112.28, 112.18, 111.77, 100.84, 58.88, 55.76, 53.31, 53.14, 46.19, 41.35, 21.34 ppm; IR (CH_2Cl_2): ν 1713, 1608, 1557 cm^{-1}; HRMS: Calcd for $C_{17}H_{21}N_2O_4$ (M + H) 317.1457; found: 317.1496.

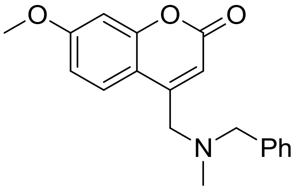

C7A13

4-((Benzyl(methyl)amino)methyl)-7-methoxy-2*H*-chromen-2-one: ^{1}H NMR (500 MHz, $CDCl_3$) δ 7.67 (d, *J* = 10 Hz, 1H), 7.33–7.25 (m, 5H), 6.82–6.79 (m, 2H), 6.42 (t, *J* = 1 Hz, 1H), 3.85 (s, 3H), 3.61 (s, 2H), 3.58 (d, *J* = 1 Hz, 2H), 2.26 (s, 3H) ppm; ^{13}C NMR (125 MHz, $CDCl_3$) δ 162.52, 161.55, 155.61, 153.10, 138.36, 128.94, 128.42, 127.39, 126.05, 112.44, 112.12, 111.86, 100.76, 62.57, 58.15, 55.72, 42.75 ppm; IR (CH_2Cl_2): ν 1716, 1611 cm^{-1}; HRMS: Calcd for $C_{19}H_{20}NO_3$ (M + H) 310.1398; found: 310.1438.

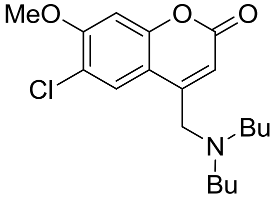

C8A3

6-Chloro-4-((dibutylamino)methyl)-7-methoxy-2*H*-chromen-2-one: ^{1}H NMR (500 MHz, $CDCl_3$) δ 7.90 (s, 1H), 6.85 (s, 1H), 6.47 (s, 1H), 3.96 (s, 3H), 3.62 (d, *J* = 5 Hz, 1H), 2.47 (t, *J* = 5 Hz, 4H), 1.45–1.43 (m, 4H), 1.34–1.28 (m, 4H), .089 (t, *J* = 5 Hz, 6H) ppm; ^{13}C NMR (125 MHz, $CDCl_3$) δ 161.09, 157.34, 153.86, 153.62, 125.77, 118.59, 112.78, 112.40, 101.21, 56.56, 55.98, 54.23, 29.14, 20.56, 14.02 ppm; IR (CH_2Cl_2): ν 1729, 1607, 1557 cm^{-1}; HRMS: Calcd for $C_{19}H_{27}ClNO_3$ (M + H) 352.1635; found: 352.1673.

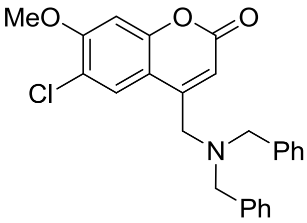

C8A12

6-Chloro-4-((dibenzylamino)methyl)-7-methoxy-2*H*-chromen-2-one: ^{1}H NMR (500 MHz, $CDCl_3$) δ 7.79 (s, 1H), 7.34–7.25 (m, 10H), 6.80 (s, 1H), 6.51 (s, 1H), 3.93 (s, 3H), 3.61–3.60 (m, 6H) ppm; ^{13}C NMR (125 MHz, $CDCl_3$) δ 160.77, 157.50, 153.94, 152.56, 138.12, 129.01, 128.59, 127.52, 126.21, 118.69, 113.13, 112.57, 101.19, 58.94, 56.60, 55.08 ppm; IR (CH_2Cl_2): ν 1728, 1607, 1553 cm^{-1}; HRMS: Calcd for $C_{25}H_{23}ClNO_3$ (M + H); 420.1322 found: 420.1357.

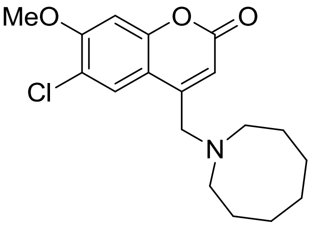

C8A14

4-(Azocan-1-ylmethyl)-6-chloro-7-methoxy-2*H*-chromen-2-one: ^{1}H NMR (500 MHz, $CDCl_3$) δ 8.01(s, 1H), 6.85 (s, 1H), 6.33 (s, 1H), 3.97 (s, 3H), 3.66 (d, *J* = 0.8 Hz, 2H), 2.63 (bs, 4H), 1.61–1.55 (m, 10H) ppm; ^{13}C NMR (125 MHz, $CDCl_3$) δ 161.01, 157.41, 153.97, 153.09, 126.77, 118.61, 112.97, 112.85, 100.15, 60.86, 56.58, 55.01, 27.76, 27.38, 25.75 ppm; IR (CH_2Cl_2): ν 1729, 1607, 1555 cm^{-1}; HRMS: Calcd for $C_{18}H_{23}ClNO_3$ (M + H); 336.1322 found: 336.1359.

C1A5

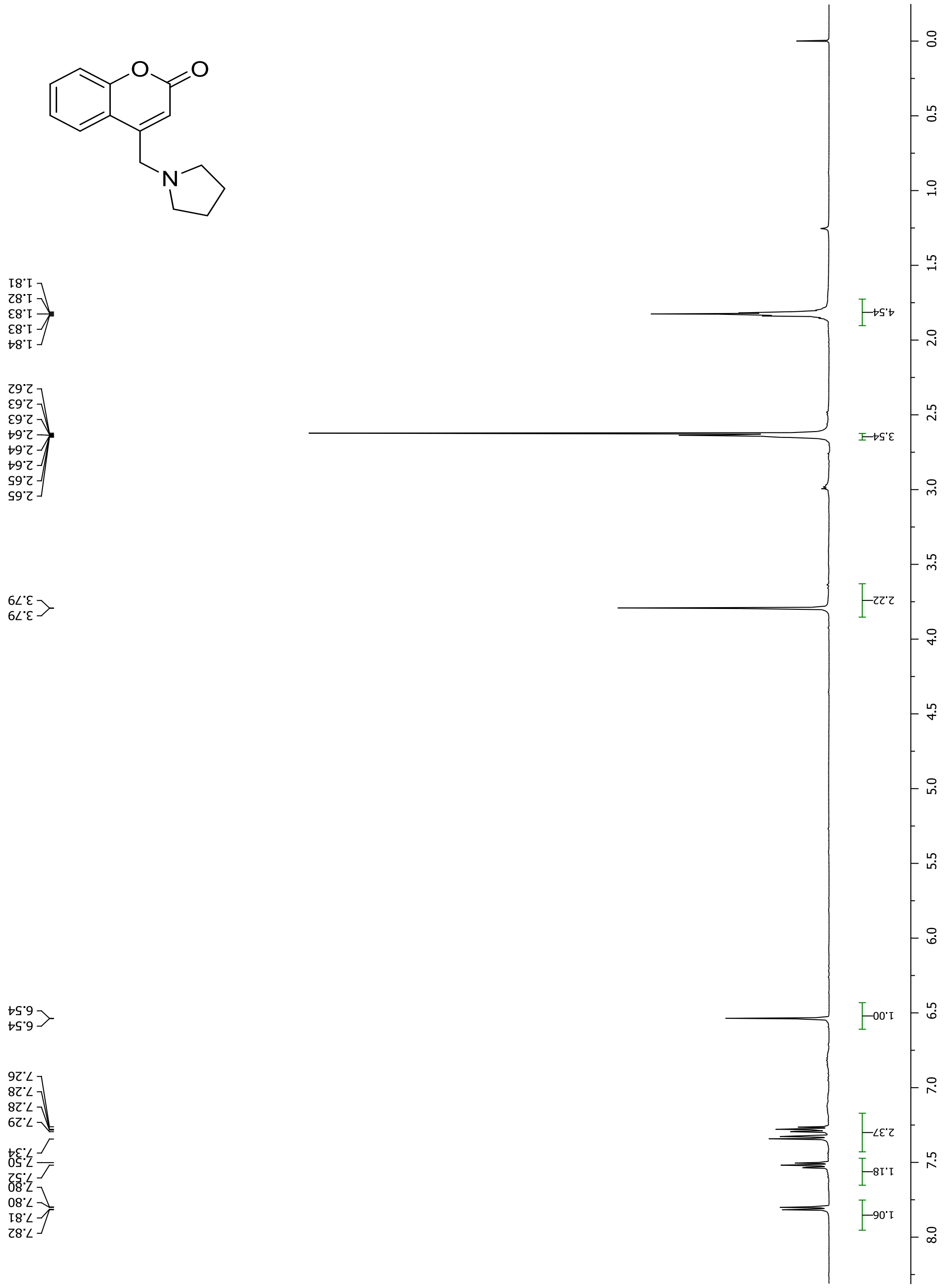

1.81
1.82
1.83
1.83
1.84
2.62
2.63
2.63
2.64
2.64
2.64
2.65
2.65
3.79
3.79
6.54
6.54
7.26
7.28
7.28
7.29
7.34
7.50
7.52
7.80
7.80
7.81
7.82
4.54
3.54
2.22
1.00
2.37
1.18
1.06
0.0
0.5
1.0
1.5
2.0
2.5
3.0
3.5
4.0
4.5
5.0
5.5
6.0
6.5
7.0
7.5
8.0

C1A5

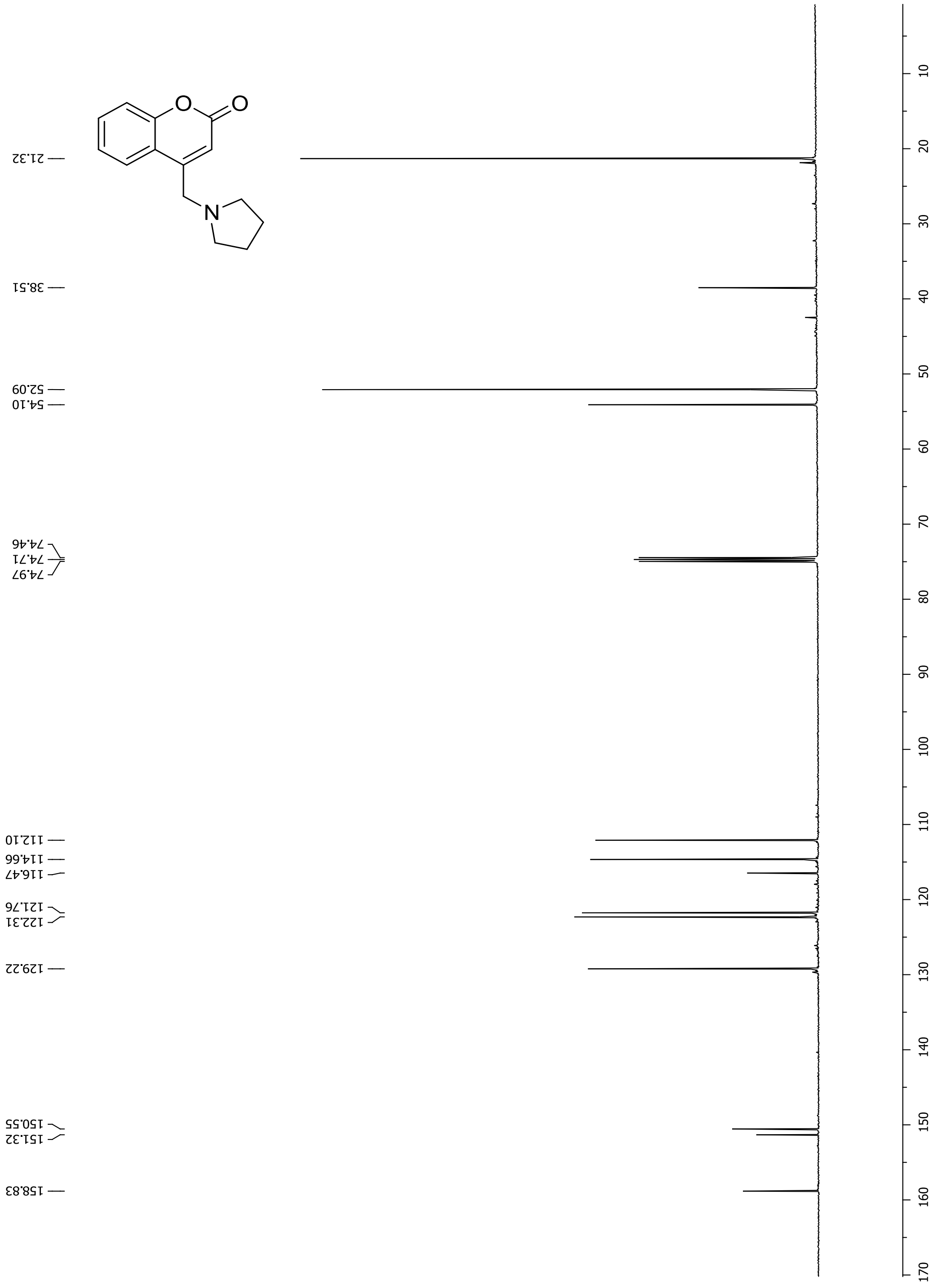
21.32
38.51
52.09
54.10
74.46
74.71
74.97
112.10
114.66
116.47
121.76
122.31
129.22
150.55
151.32
158.83
O
O
N
10
20
30
40
50
60
70
80
90
100
110
120
130
140
150
160
170

C1A7

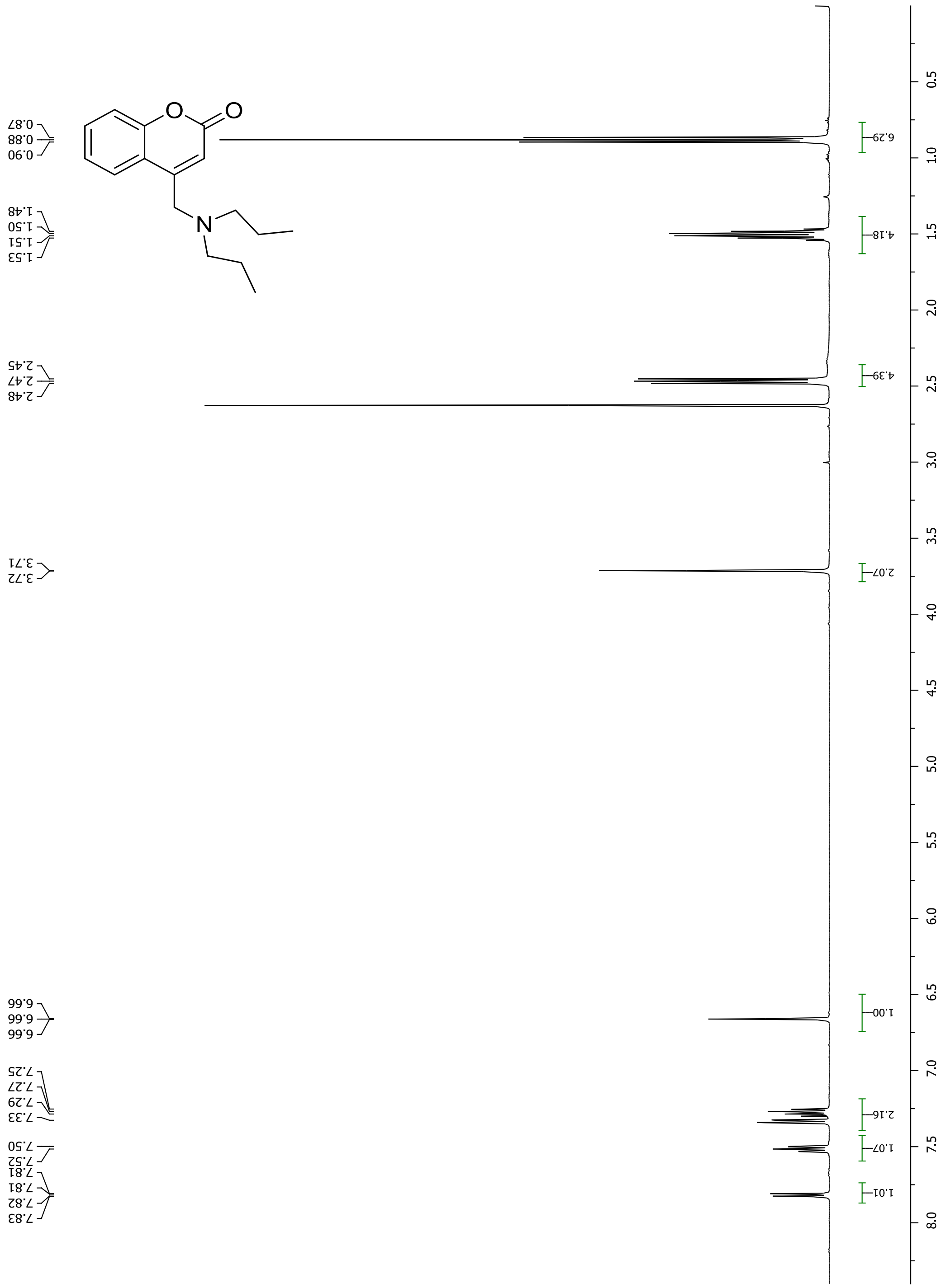

C1A7

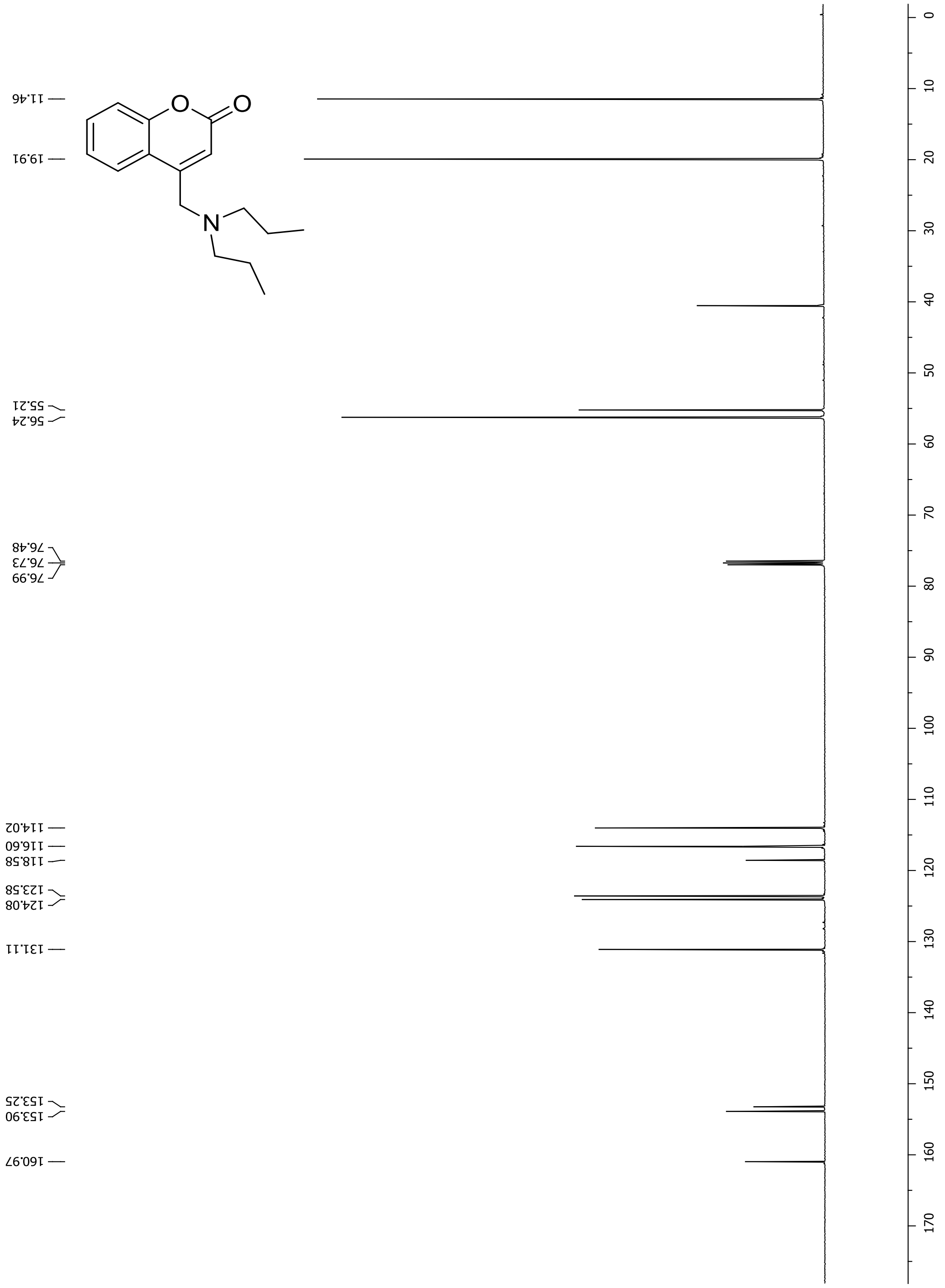
11.46
19.91
55.21
56.24
76.48
76.73
76.99
114.02
116.60
118.58
123.58
124.08
131.11
153.25
153.90
160.97
0
10
20
30
40
50
60
70
80
90
100
110
120
130
140
150
160
170

C1A8

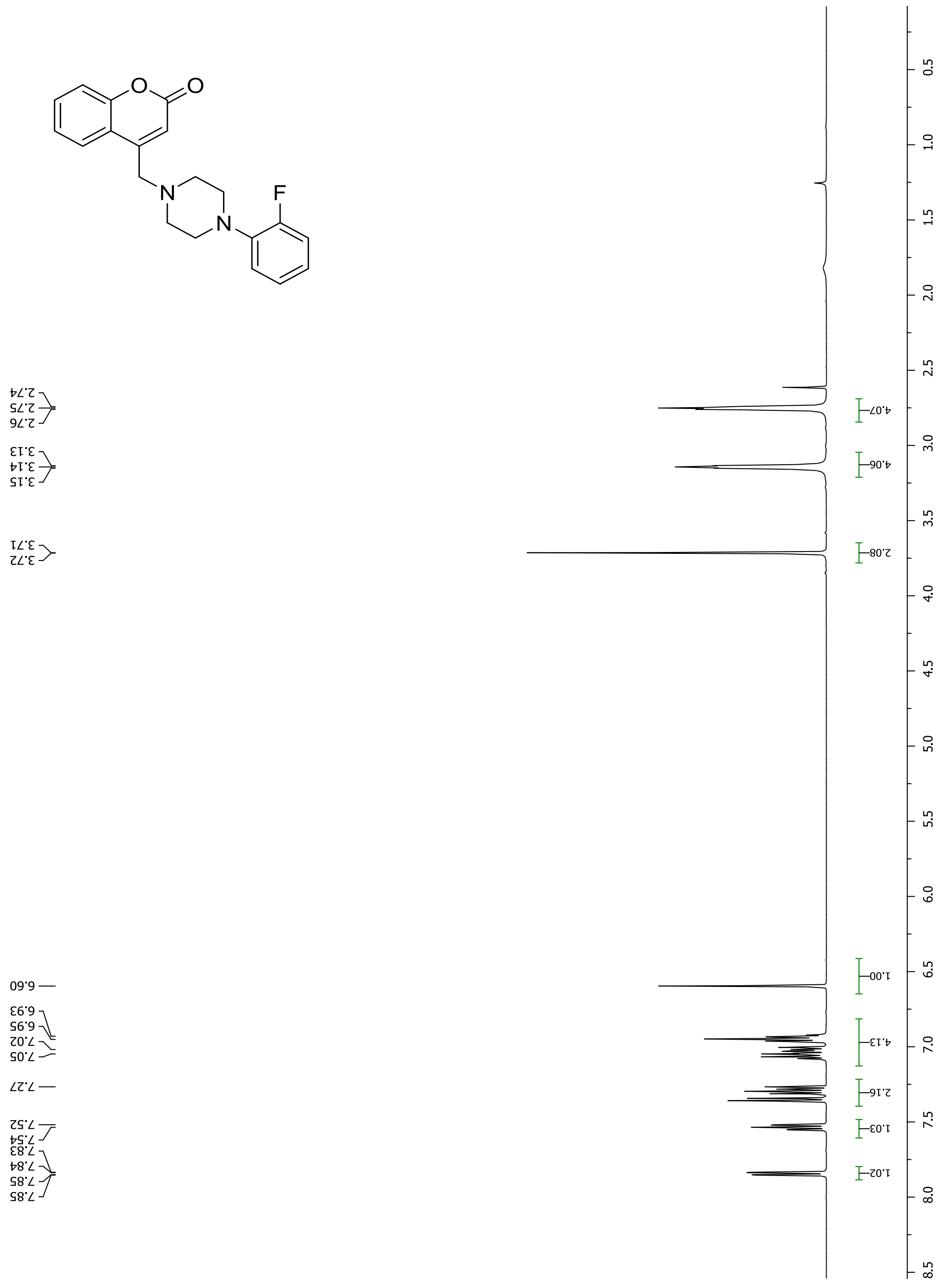
O
O
N
N
F
2.74
2.75
2.76
3.13
3.14
3.15
3.71
3.72
6.60
6.93
6.95
7.02
7.05
7.27
7.52
7.54
7.83
7.84
7.85
7.85
4.07
4.06
2.08
1.00
4.13
2.16
1.03
1.02

C1A8

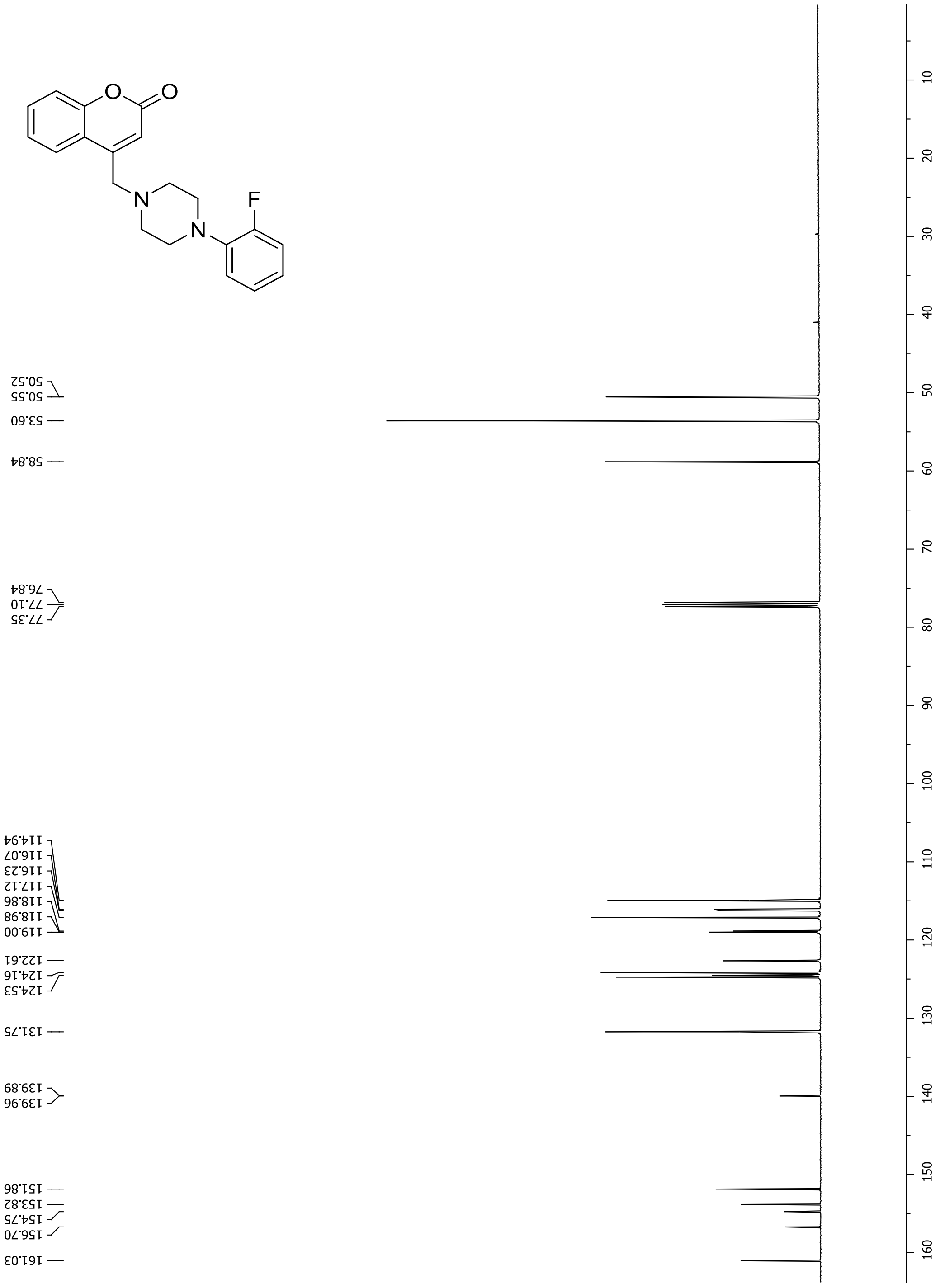
50.52
50.55
53.60
58.84
76.84
77.10
77.35
114.94
116.07
116.23
117.12
118.86
118.98
119.00
122.61
124.16
124.53
131.75
139.89
139.96
151.86
153.82
154.75
156.70
161.03

C1A14

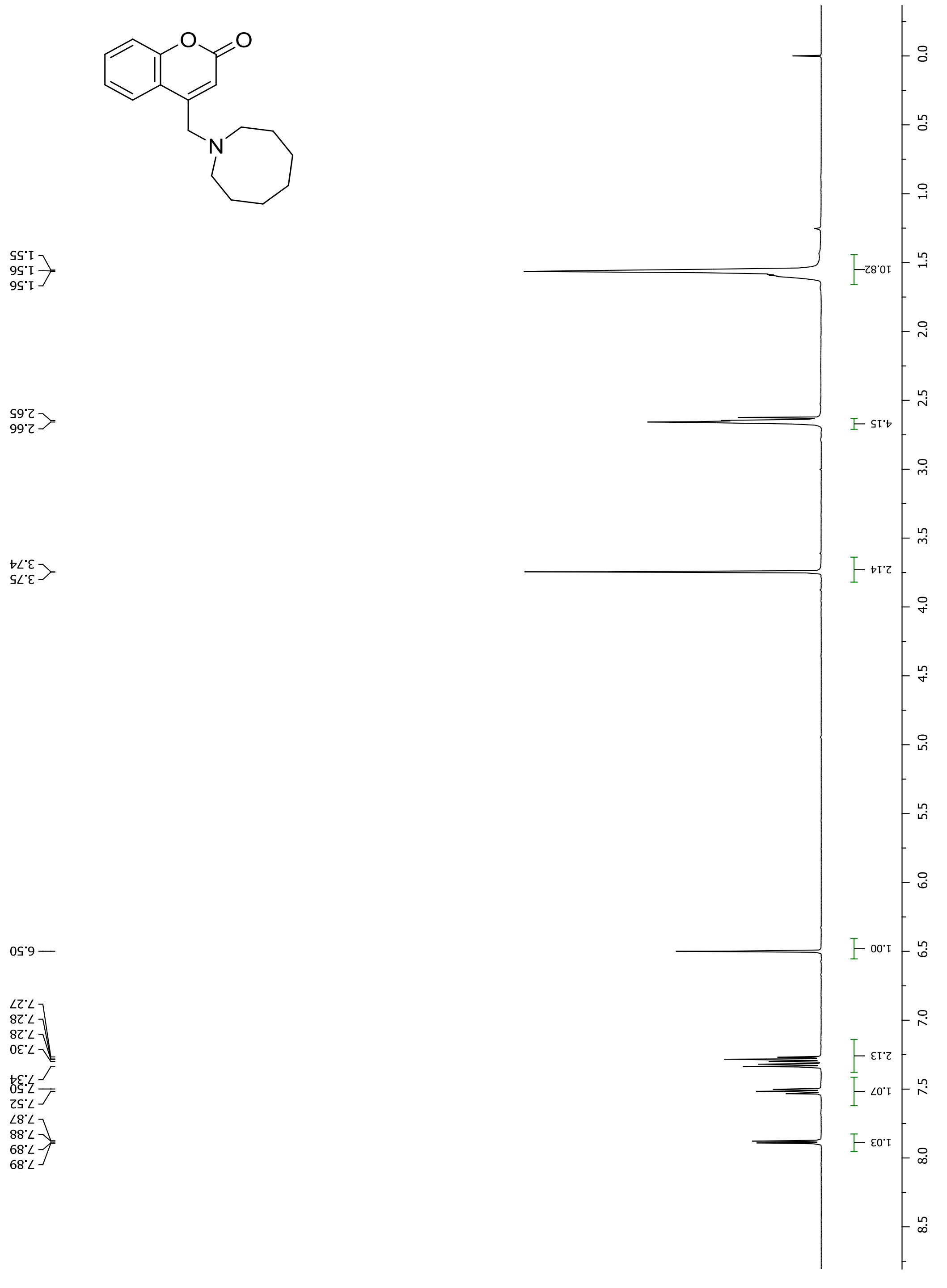
1.55
1.56
1.56
2.65
2.66
3.74
3.75
6.50
7.27
7.28
7.28
7.30
7.34
7.50
7.52
7.87
7.88
7.89
7.89
10.82
4.15
2.14
1.00
2.13
1.07
1.03
0.0
0.5
1.0
1.5
2.0
2.5
3.0
3.5
4.0
4.5
5.0
5.5
6.0
6.5
7.0
7.5
8.0
8.5

C1A14

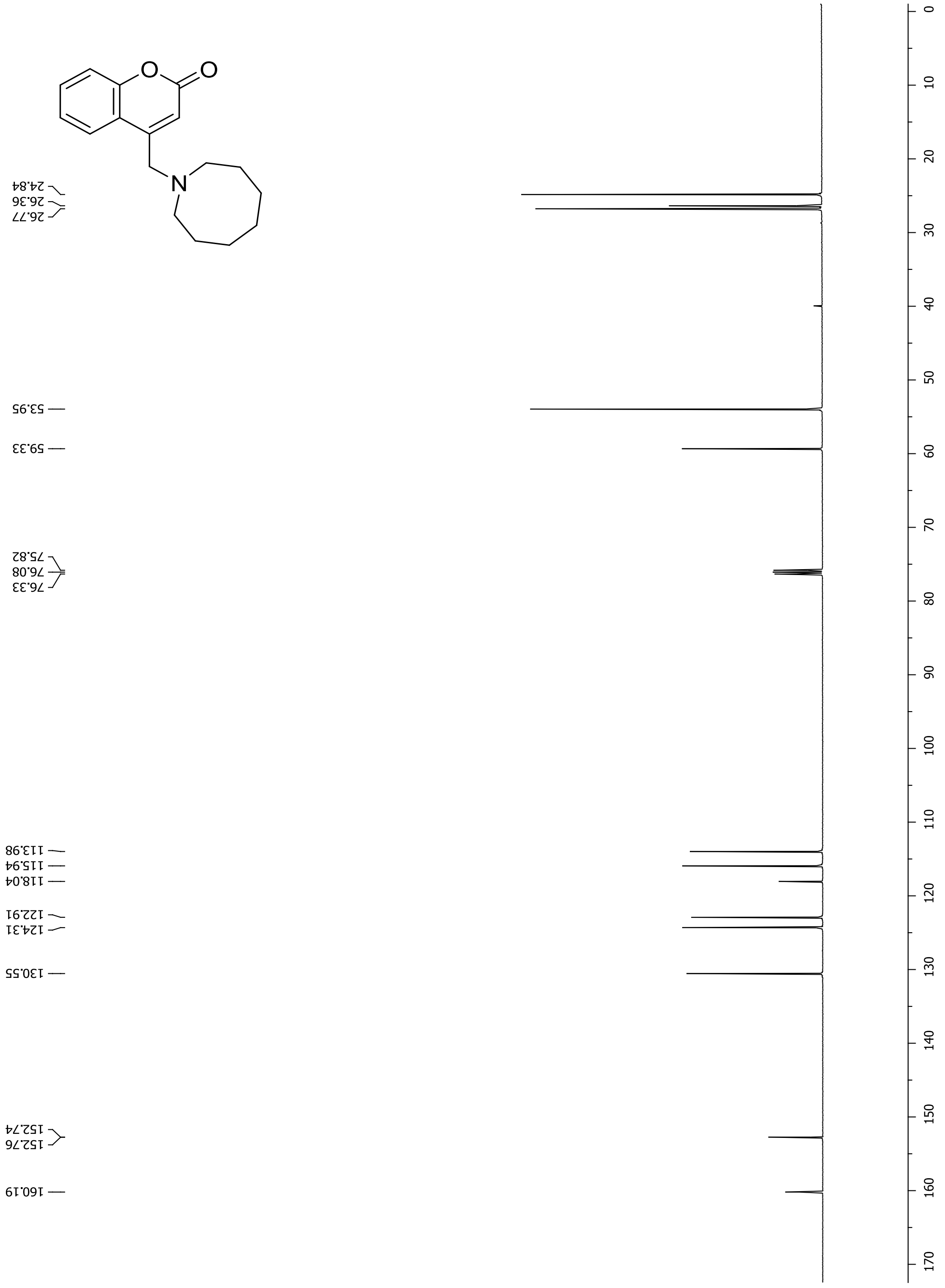

O
O
N
24.84
26.36
26.77
53.95
59.33
75.82
76.08
76.33
113.98
115.94
118.04
122.91
124.31
130.55
152.74
152.76
160.19

C2A6

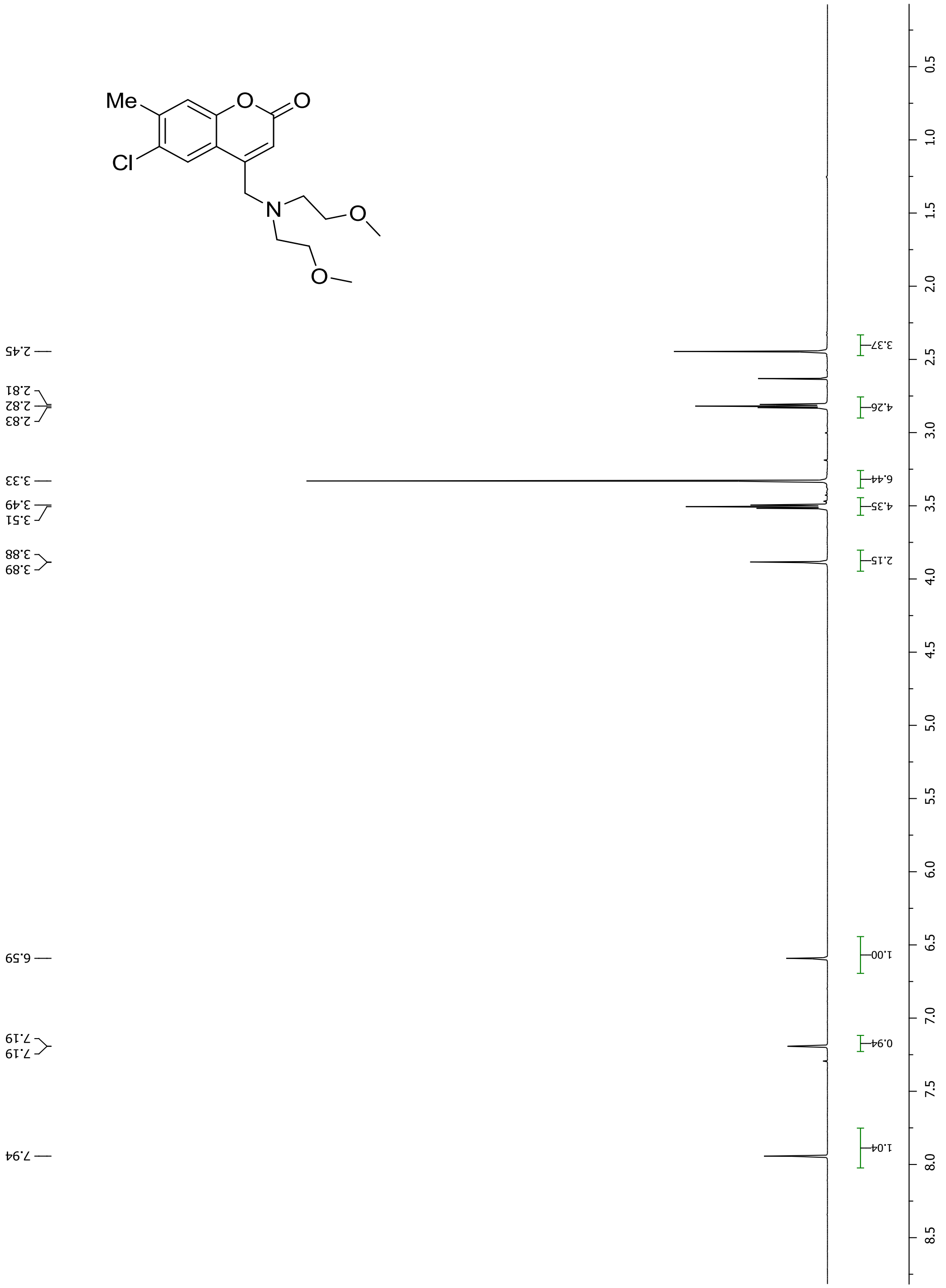

C2A6

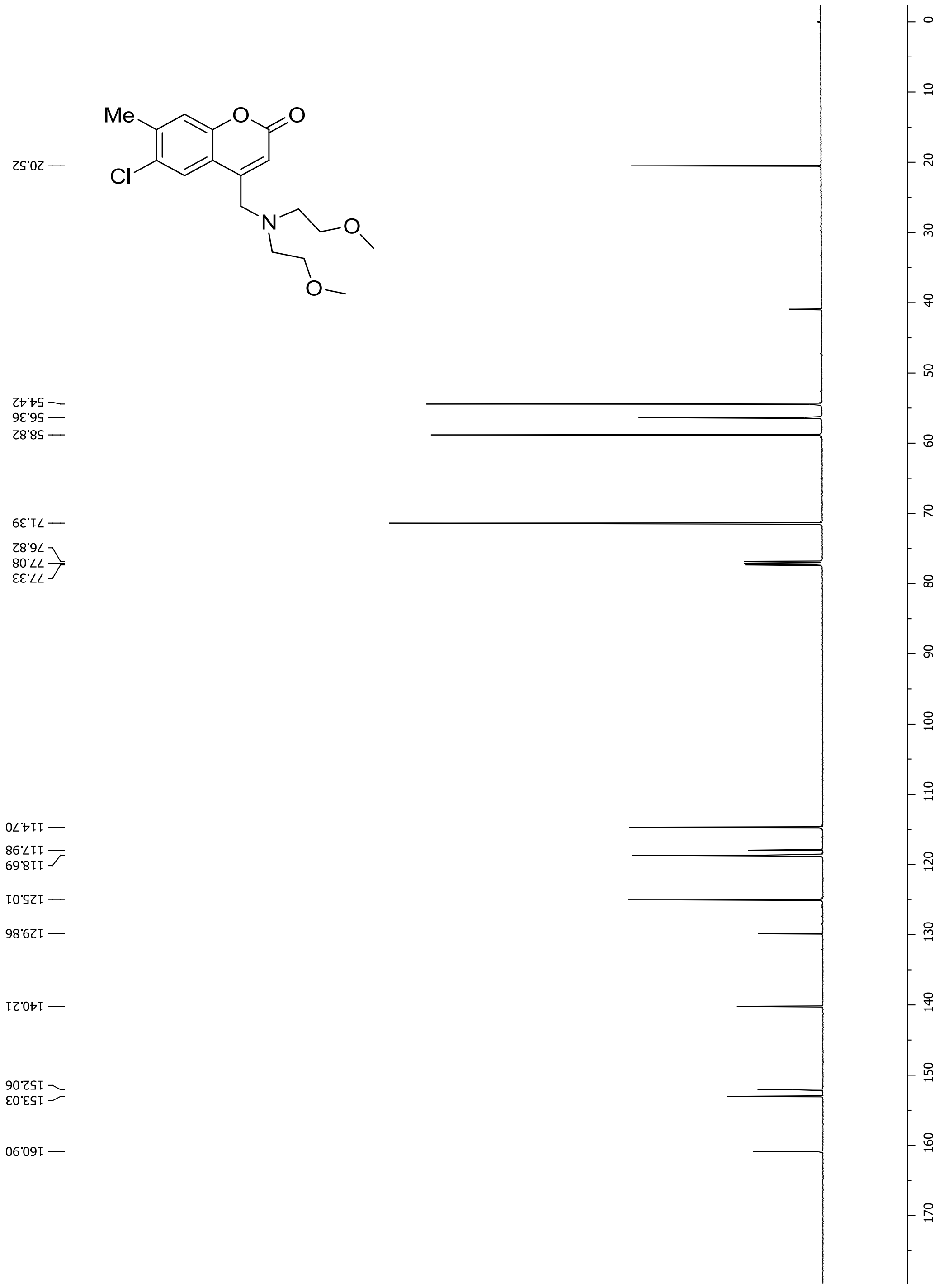
Me
O
O
Cl
N
O
O
20.52
54.42
56.36
58.82
71.39
76.82
77.08
77.33
114.70
117.98
118.69
125.01
129.86
140.21
152.06
153.03
160.90
0 10 20 30 40 50 60 70 80 90 100 110 120 130 140 150 160 170

C2A10

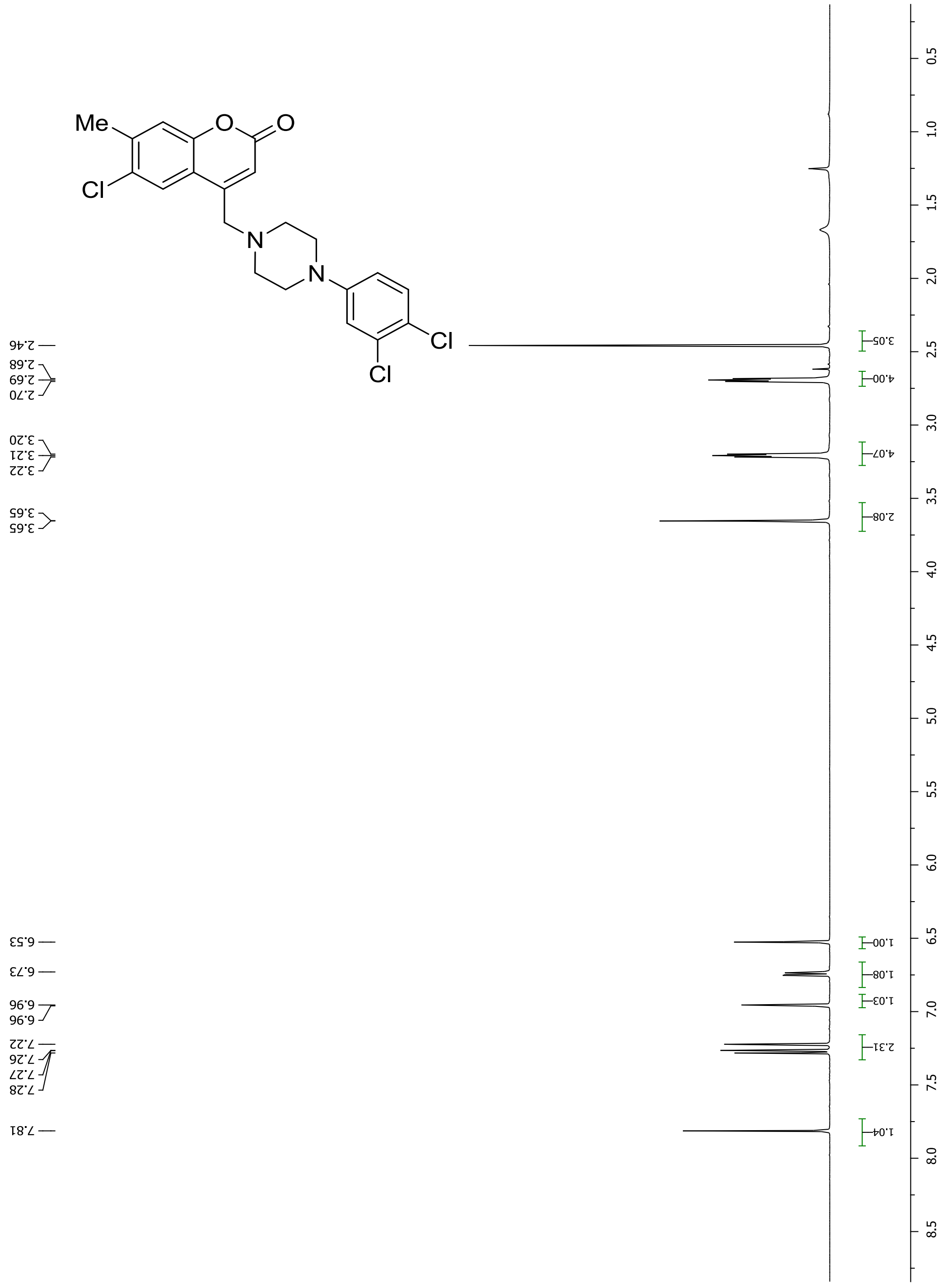
Me
O
O
Cl
N
N
Cl
Cl
2.46
2.68
2.69
2.70
3.20
3.21
3.22
3.65
3.65
6.53
6.73
6.96
6.96
7.22
7.26
7.27
7.28
7.81
3.05
4.00
4.07
2.08
1.00
1.08
1.03
2.31
1.04
0.5
1.0
1.5
2.0
2.5
3.0
3.5
4.0
4.5
5.0
5.5
6.0
6.5
7.0
7.5
8.0
8.5

C2A10

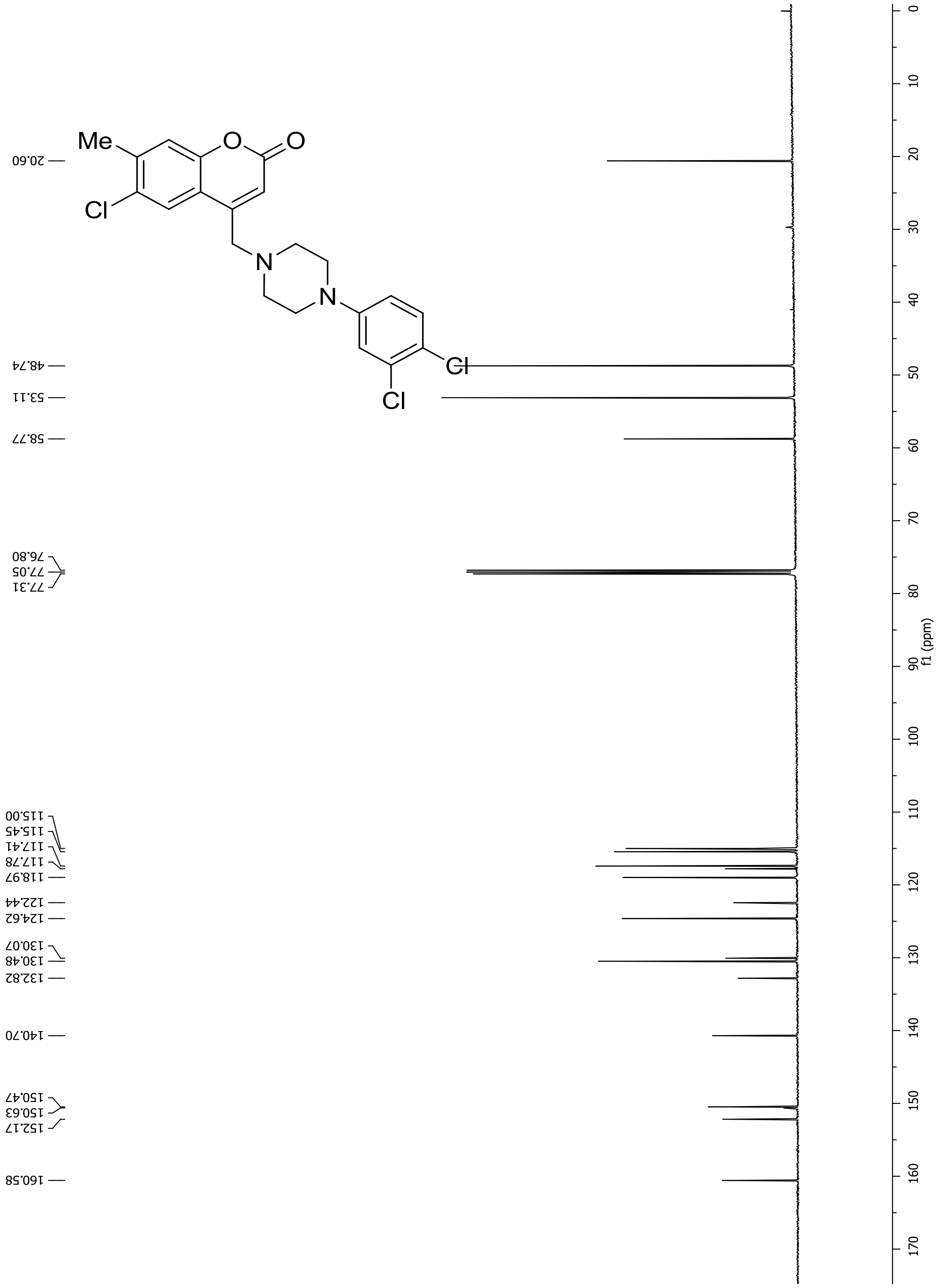
Me
O
O
Cl
N
N
Cl
Cl
20.60
48.74
53.11
58.77
76.80
77.05
77.31
115.00
115.45
117.41
117.78
118.97
122.44
124.62
130.07
130.48
132.82
140.70
150.47
150.63
152.17
160.58
f1 (ppm)

C2A12

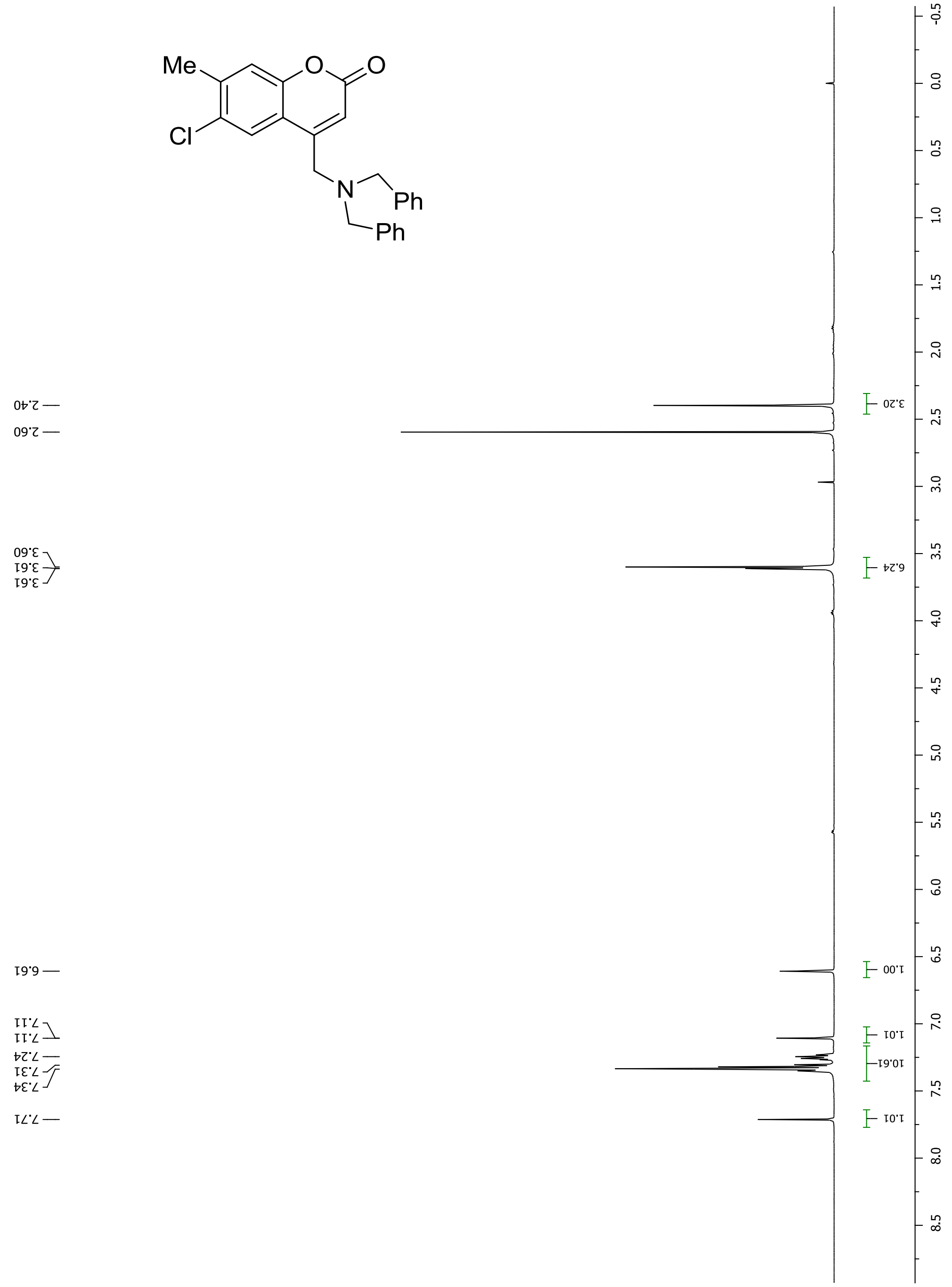
Me
O
O
Cl
N
Ph
Ph
2.40
2.60
3.60
3.61
3.61
6.61
7.11
7.11
7.24
7.31
7.34
7.71
3.20
6.24
1.00
1.01
10.61
1.01

C2A12

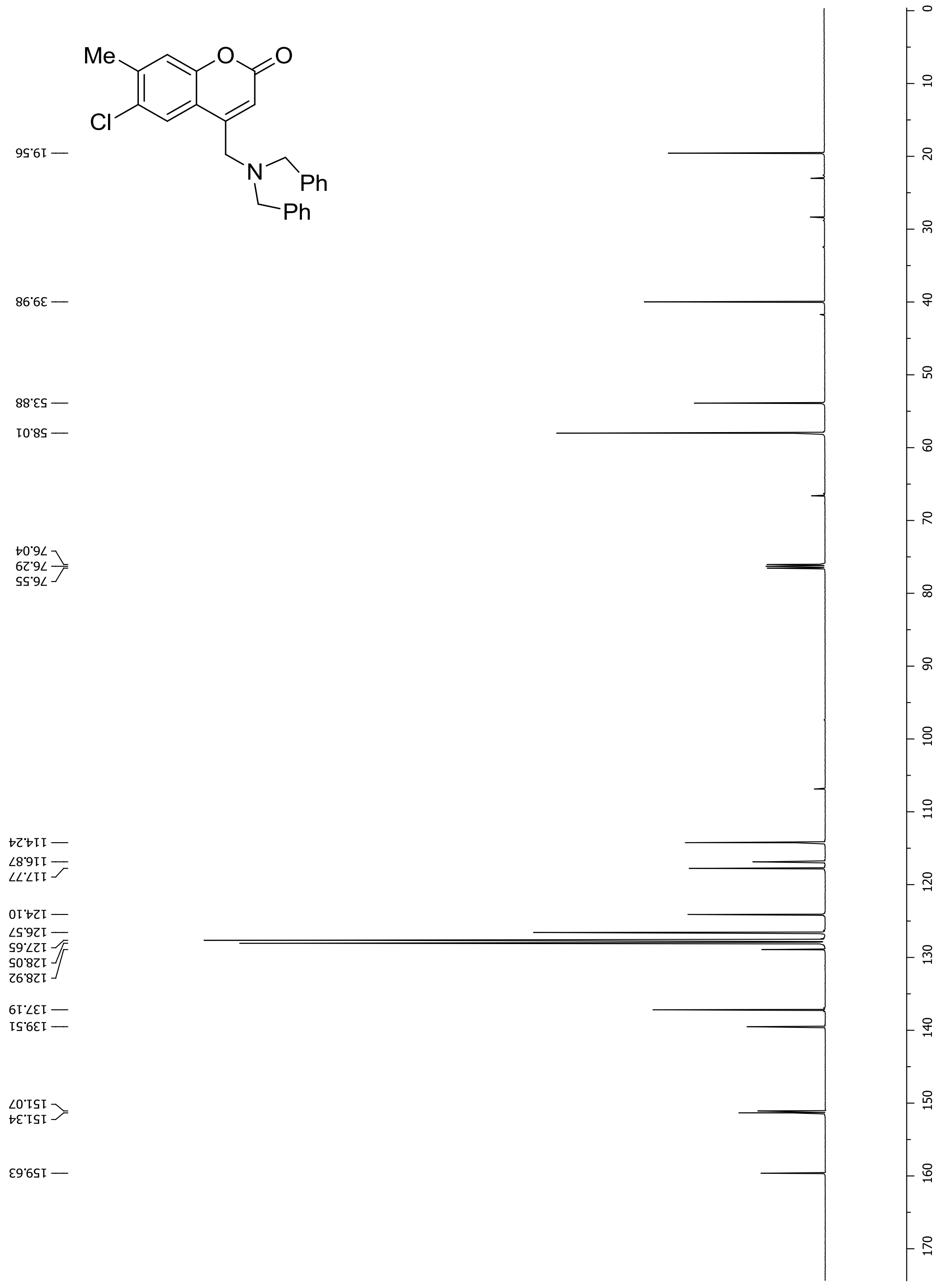

Me
O
O
Cl
N
Ph
Ph
19.56
39.98
53.88
58.01
76.04
76.29
76.55
114.24
116.87
117.77
124.10
126.57
127.65
128.05
128.92
137.19
139.51
151.07
151.34
159.63
0
10
20
30
40
50
60
70
80
90
100
110
120
130
140
150
160
170

C2A13

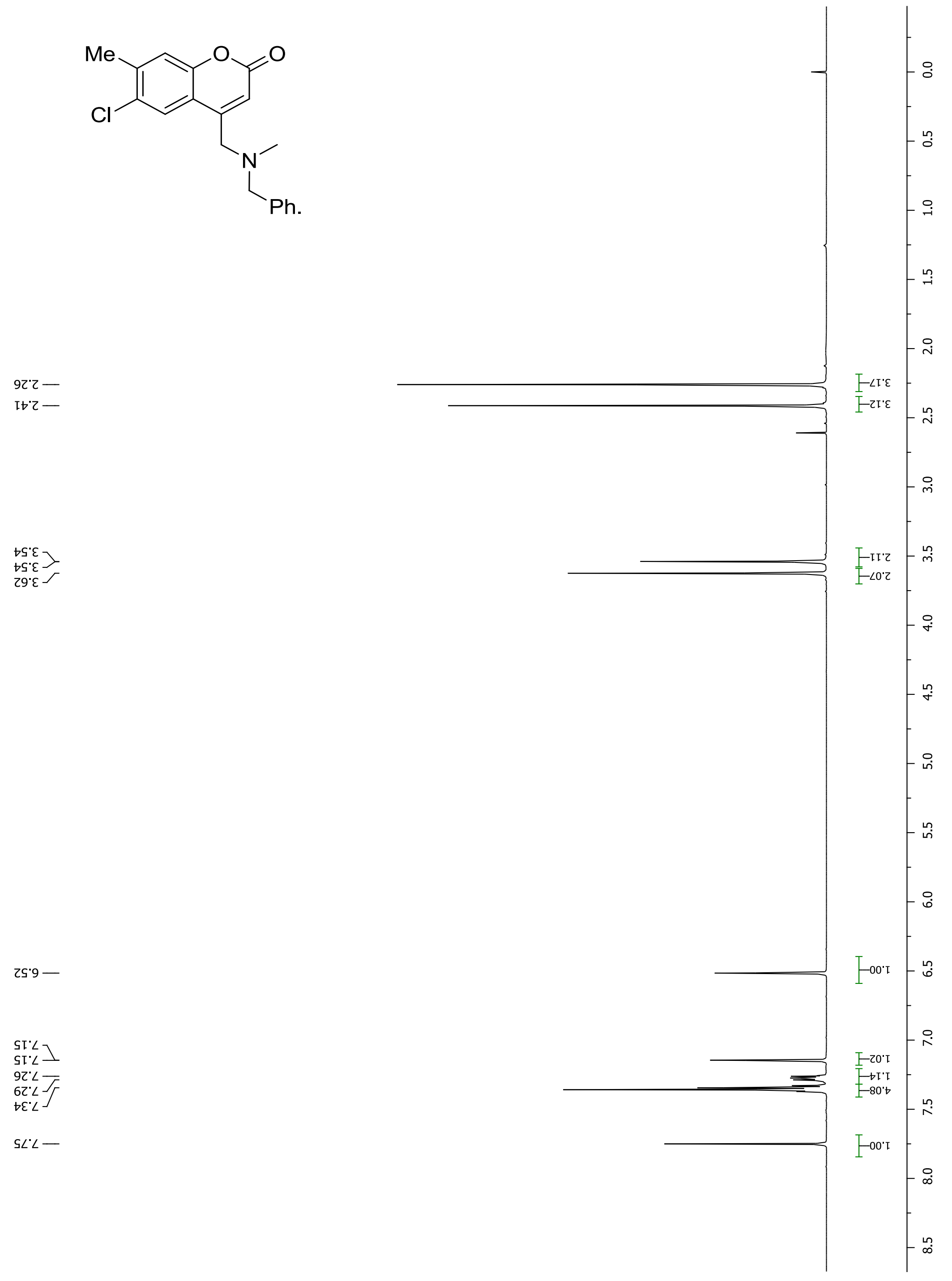

C2A13

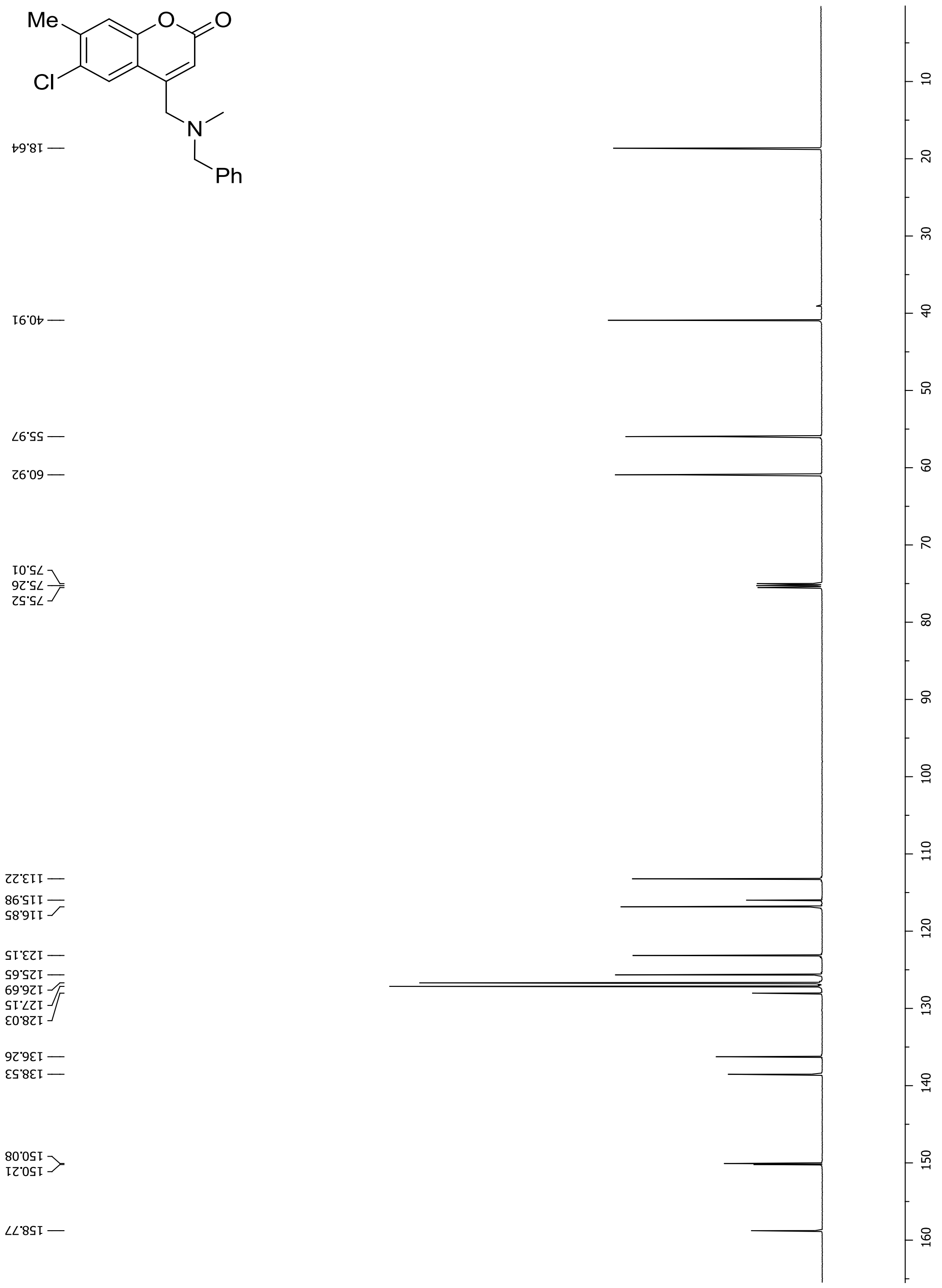
Me
O
O
Cl
N
Ph
18.64
40.91
55.97
60.92
75.01
75.26
75.52
113.22
115.98
116.85
123.15
125.65
126.69
127.15
128.03
136.26
138.53
150.08
150.21
158.77
10
20
30
40
50
60
70
80
90
100
110
120
130
140
150
160

C3A11

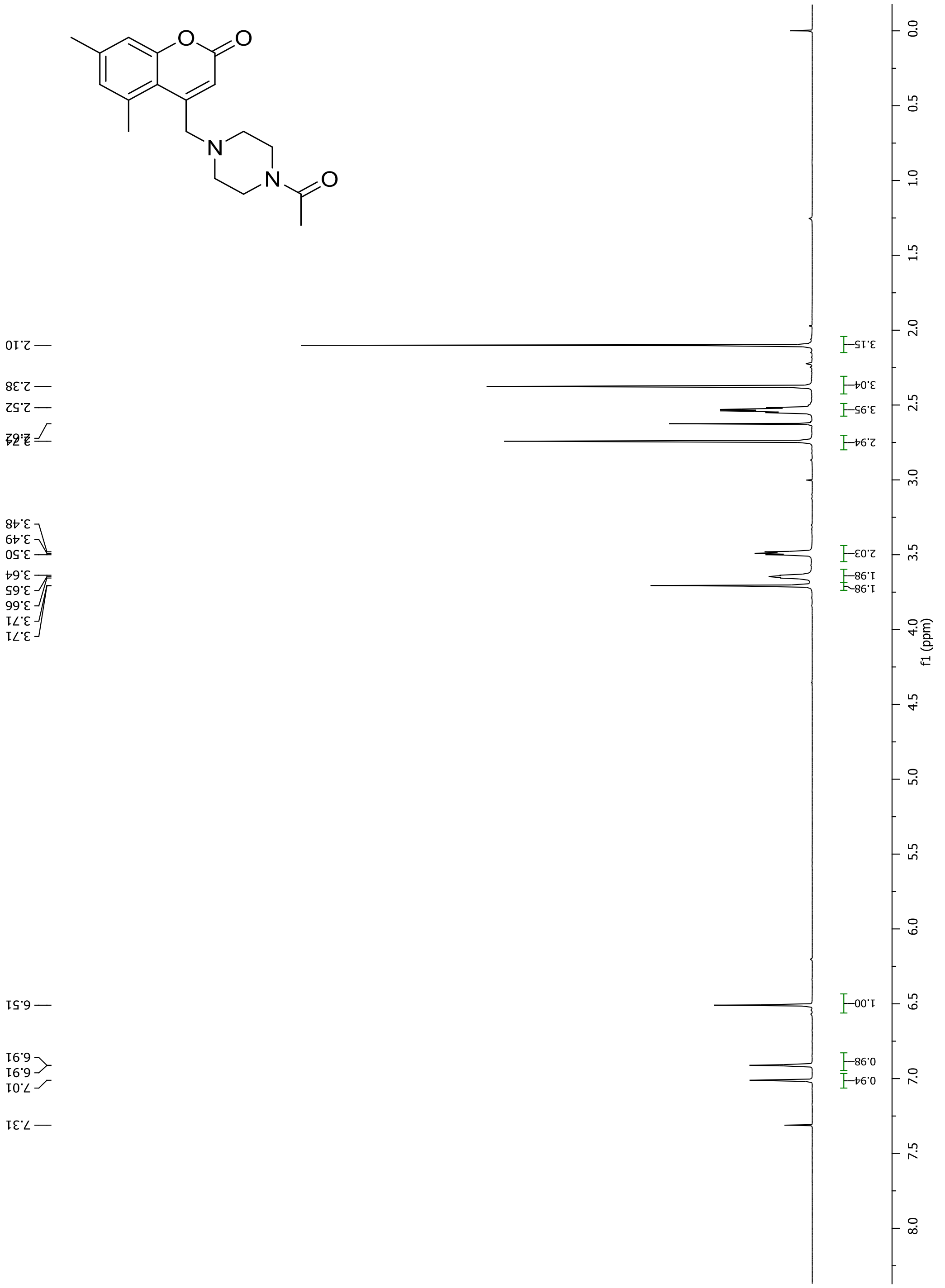

C3A11

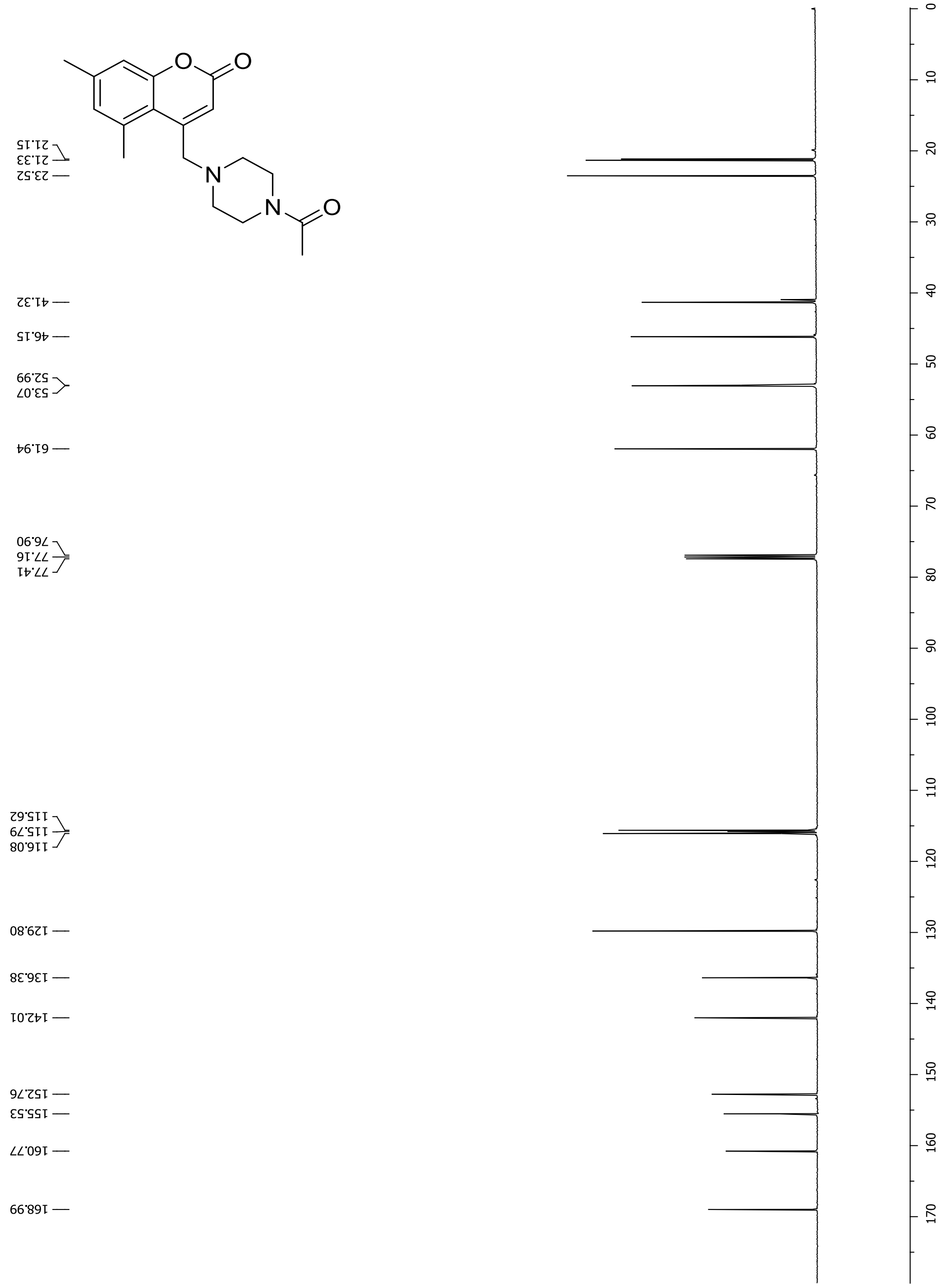
21.15
21.33
23.52
41.32
46.15
52.99
53.07
61.94
76.90
77.16
77.41
115.62
115.79
116.08
129.80
136.38
142.01
152.76
155.53
160.77
168.99
0
10
20
30
40
50
60
70
80
90
100
110
120
130
140
150
160
170

C4A1

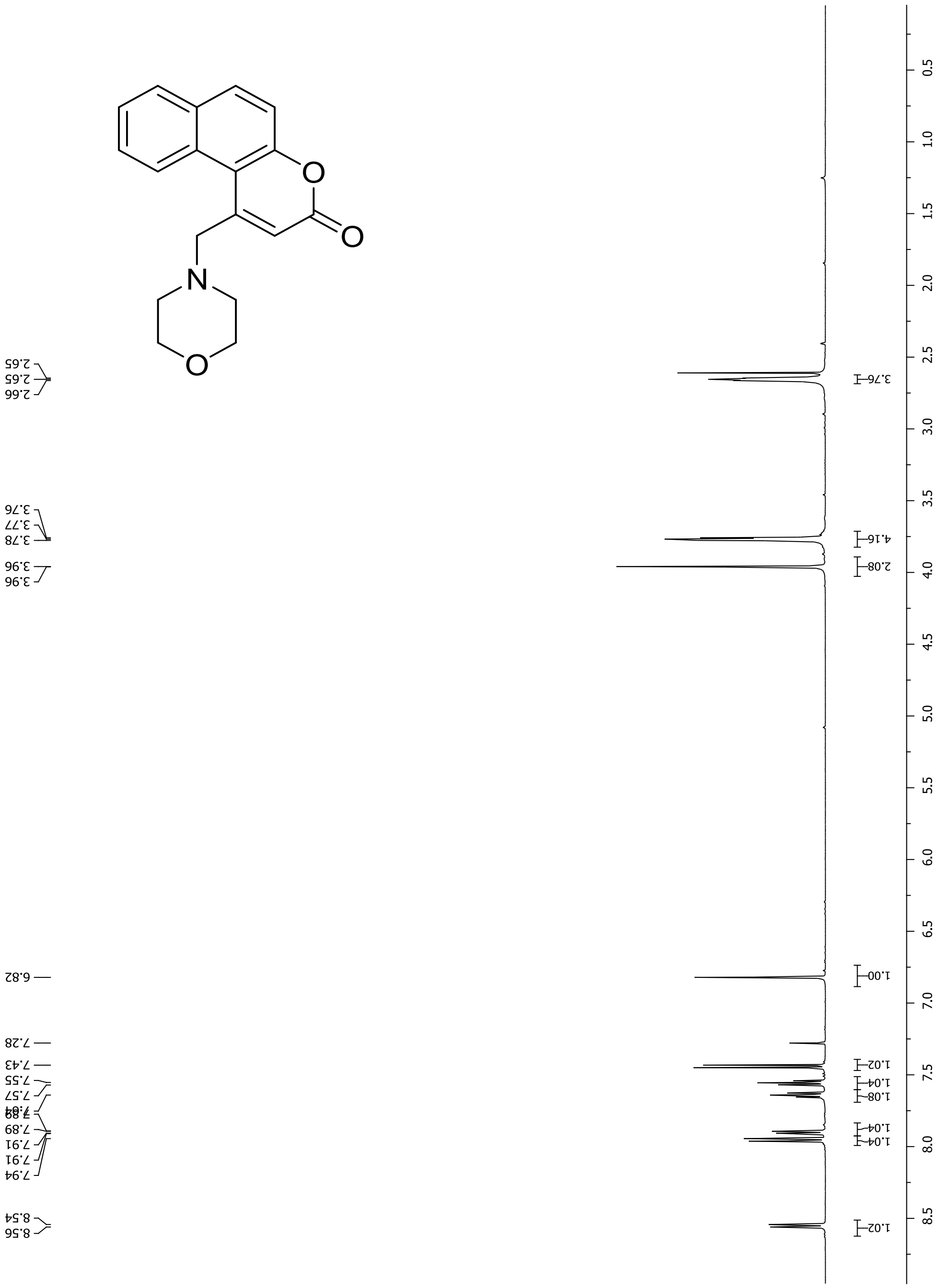

C4A1

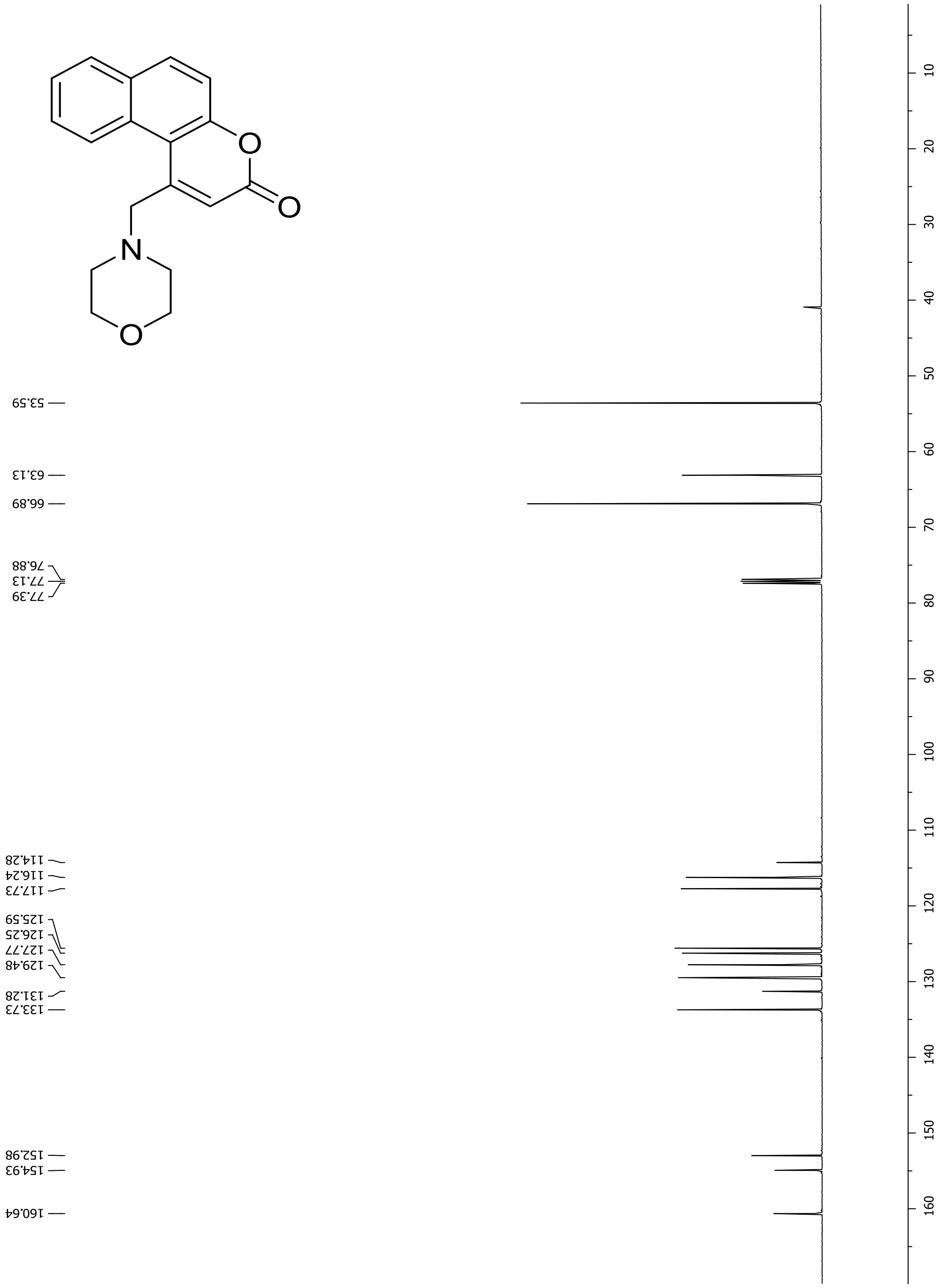
O
O
N
O
53.59
63.13
66.89
76.88
77.13
77.39
114.28
116.24
117.73
125.59
126.25
127.77
129.48
131.28
133.73
152.98
154.93
160.64
10
20
30
40
50
60
70
80
90
100
110
120
130
140
150
160

C4A2

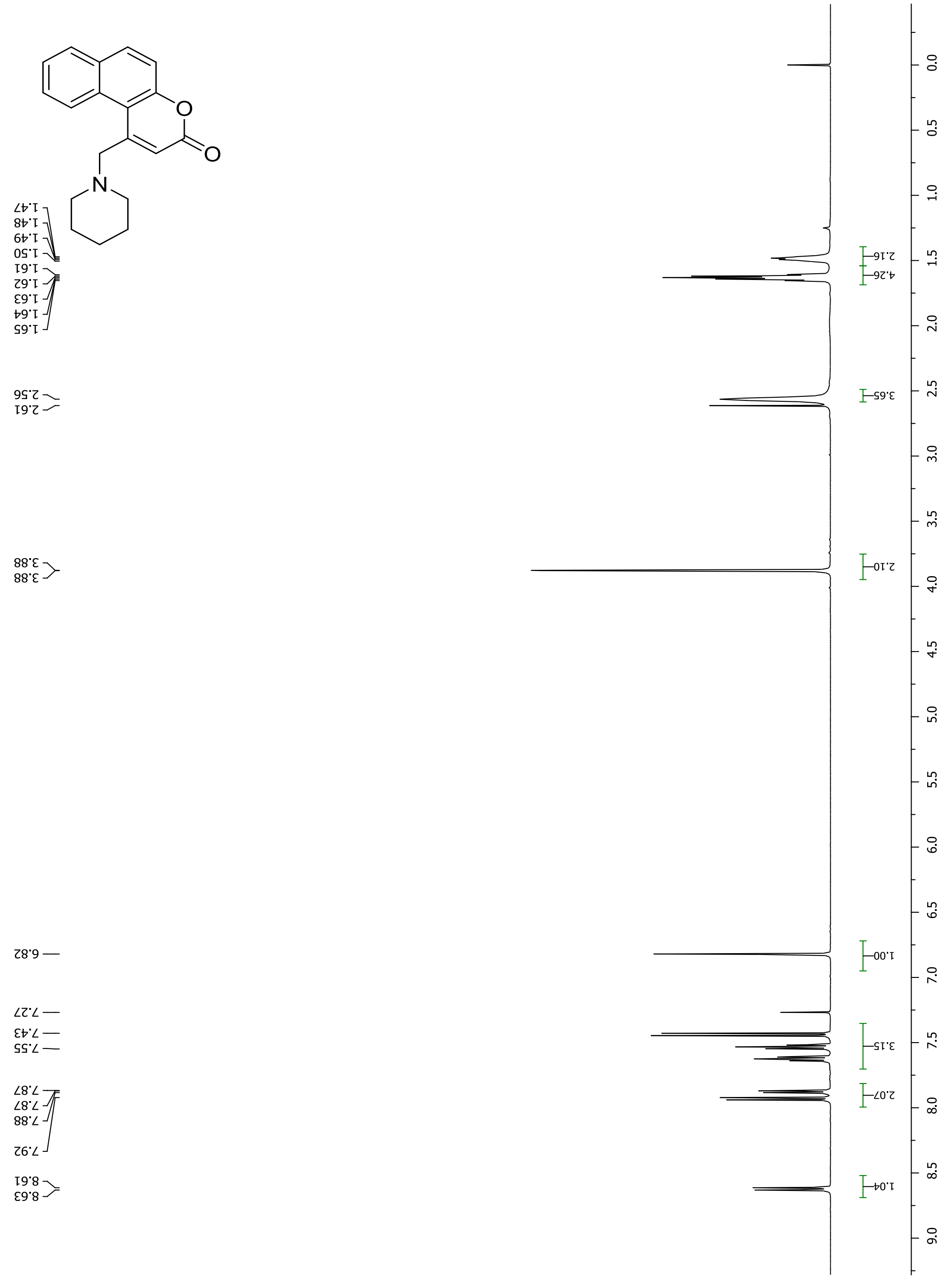
1.47
1.48
1.49
1.50
1.61
1.62
1.63
1.64
1.65
2.56
2.61
3.88
3.88
6.82
7.27
7.43
7.55
7.87
7.87
7.88
7.92
8.61
8.63
2.16
4.26
3.65
2.10
1.00
3.15
2.07
1.04
0.0
0.5
1.0
1.5
2.0
2.5
3.0
3.5
4.0
4.5
5.0
5.5
6.0
6.5
7.0
7.5
8.0
8.5
9.0

C4A2

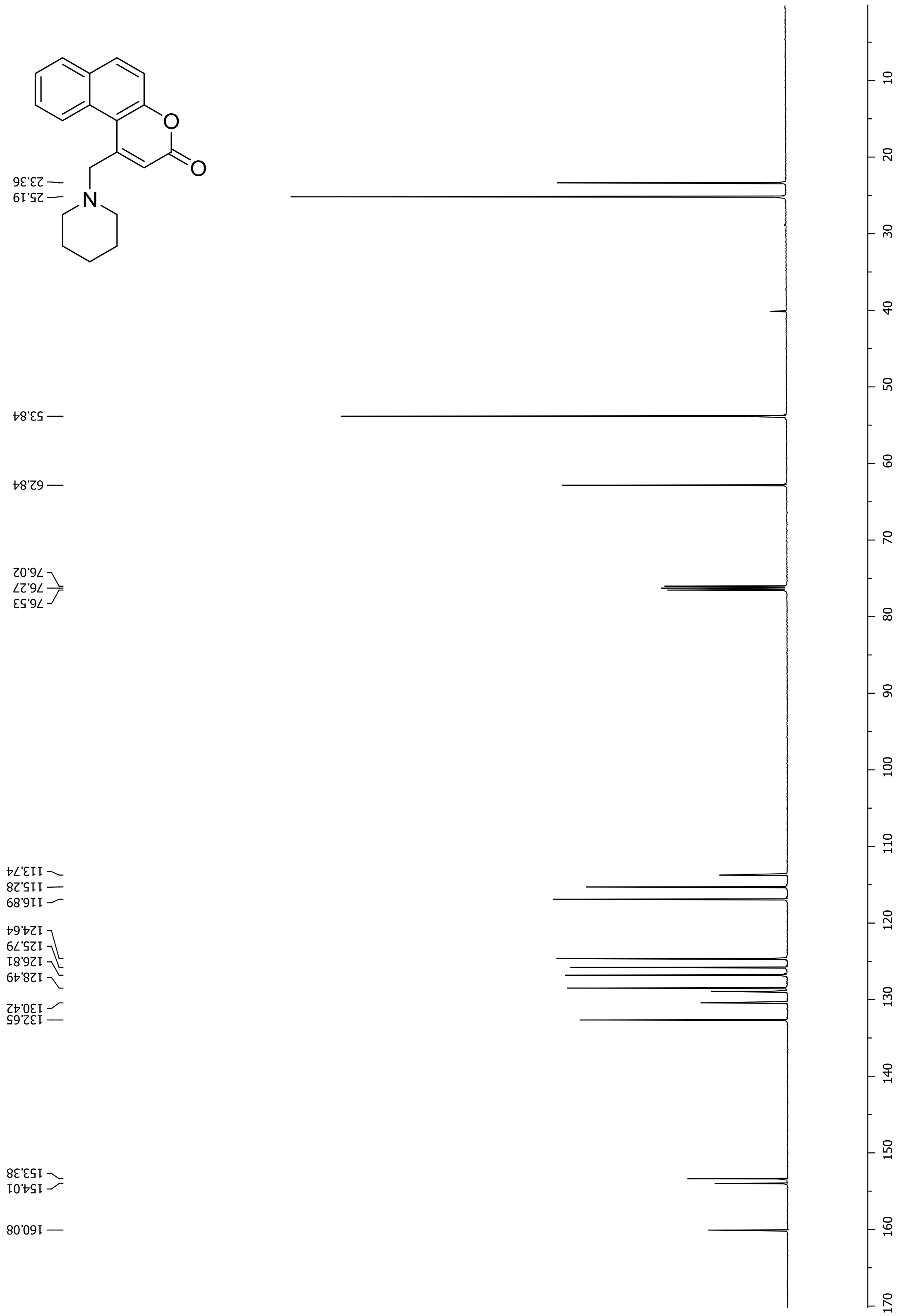
23.36
25.19
53.84
62.84
76.02
76.27
76.53
113.74
115.28
116.89
124.64
125.79
126.81
128.49
130.42
132.65
153.38
154.01
160.08
N
O
O

C4A3

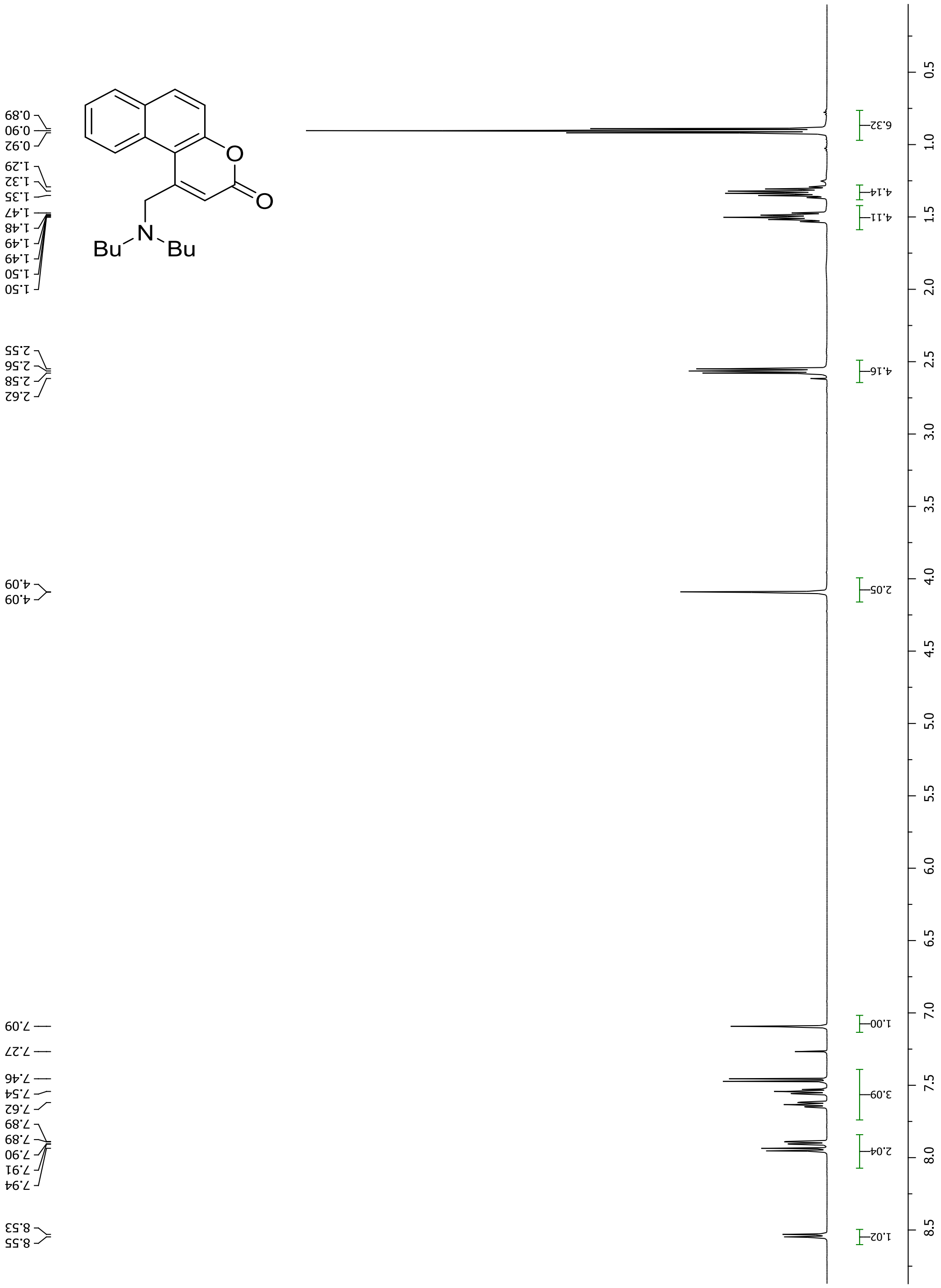

C4A3

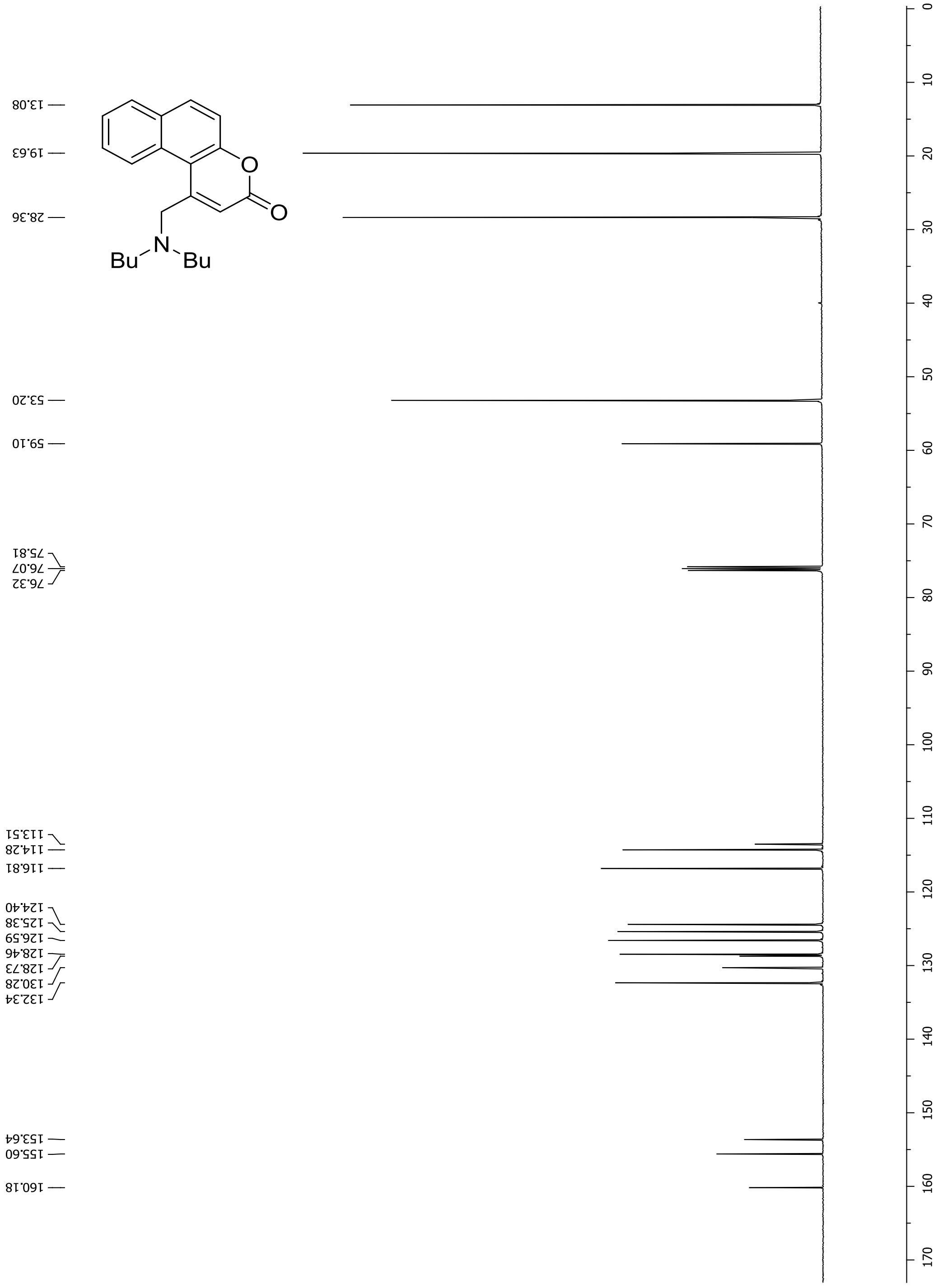
13.08
19.63
28.36
53.20
59.10
75.81
76.07
76.32
113.51
114.28
116.81
124.40
125.38
126.59
128.46
128.73
130.28
132.34
153.64
155.60
160.18
O
O
N
Bu
Bu

C4A4

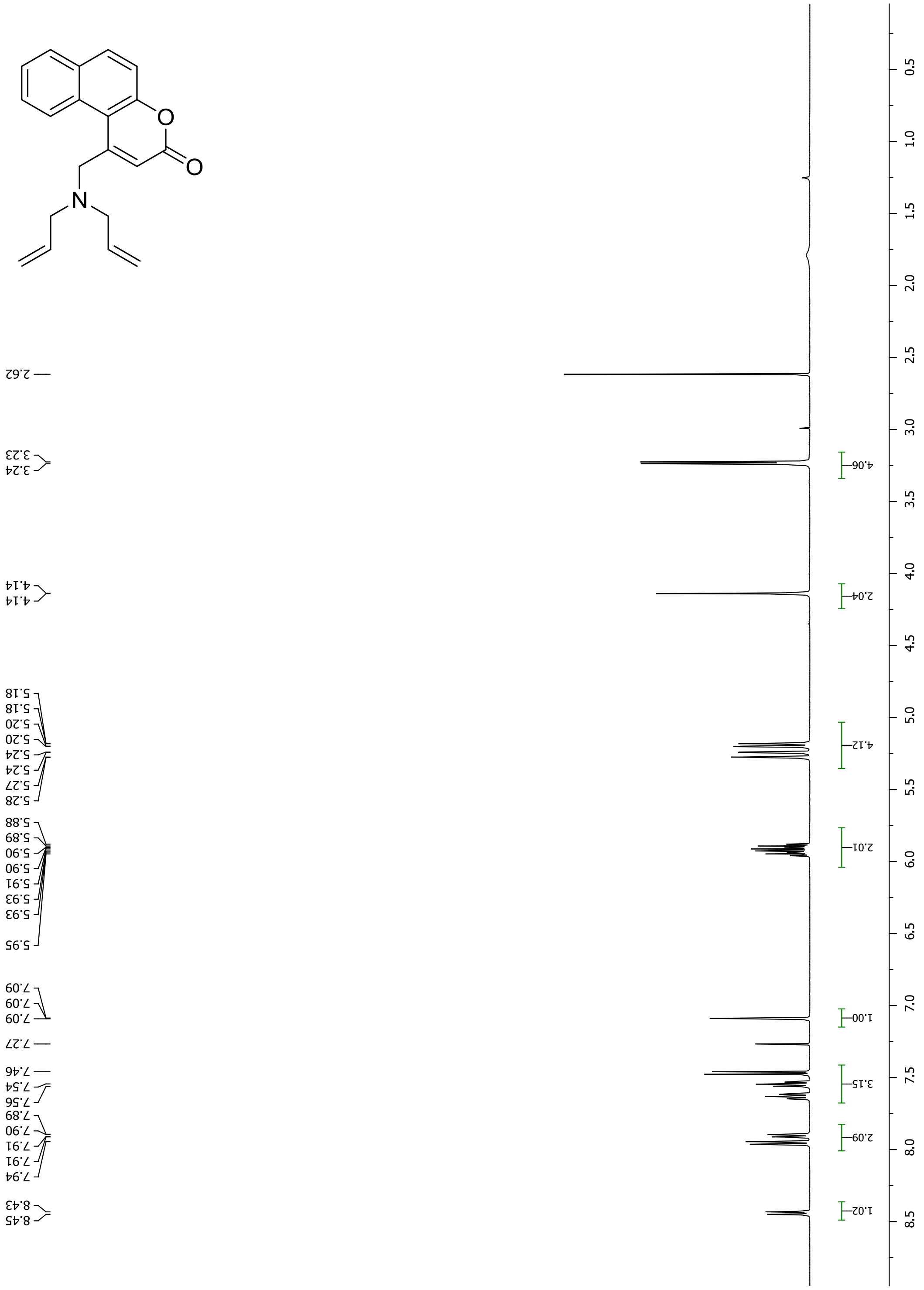

2.62
3.23
3.24
4.14
4.14
5.18
5.18
5.20
5.20
5.24
5.24
5.27
5.28
5.88
5.89
5.90
5.90
5.91
5.93
5.93
5.95
7.09
7.09
7.09
7.27
7.46
7.54
7.56
7.89
7.90
7.91
7.91
7.94
8.43
8.45
4.06
2.04
4.12
2.01
1.00
3.15
2.09
1.02

C4A4

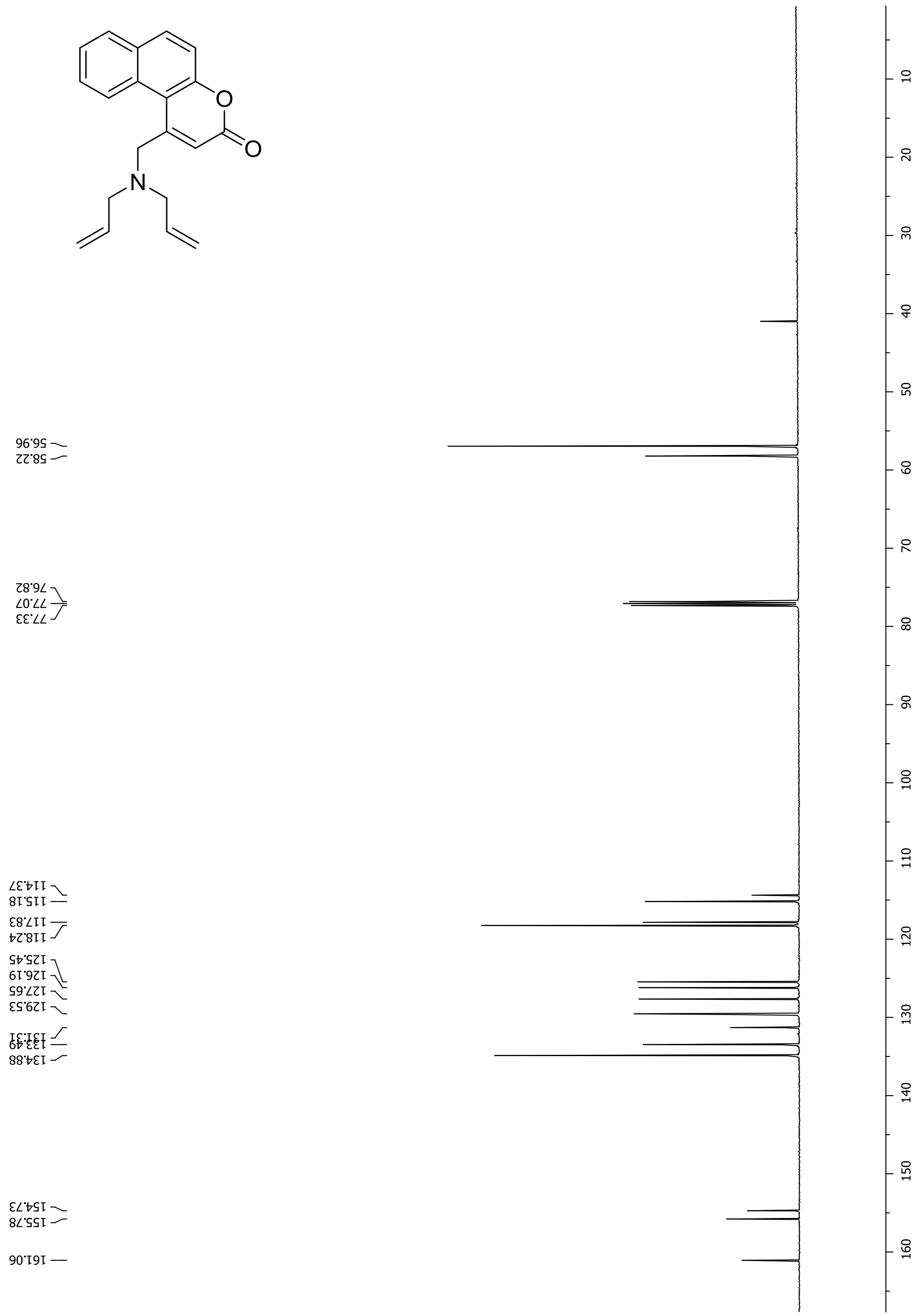
O
O
N
56.96
58.22
76.82
77.07
77.33
114.37
115.18
117.83
118.24
125.45
126.19
127.65
129.53
131.31
133.49
134.88
154.73
155.78
161.06
10
20
30
40
50
60
70
80
90
100
110
120
130
140
150
160

C4A14

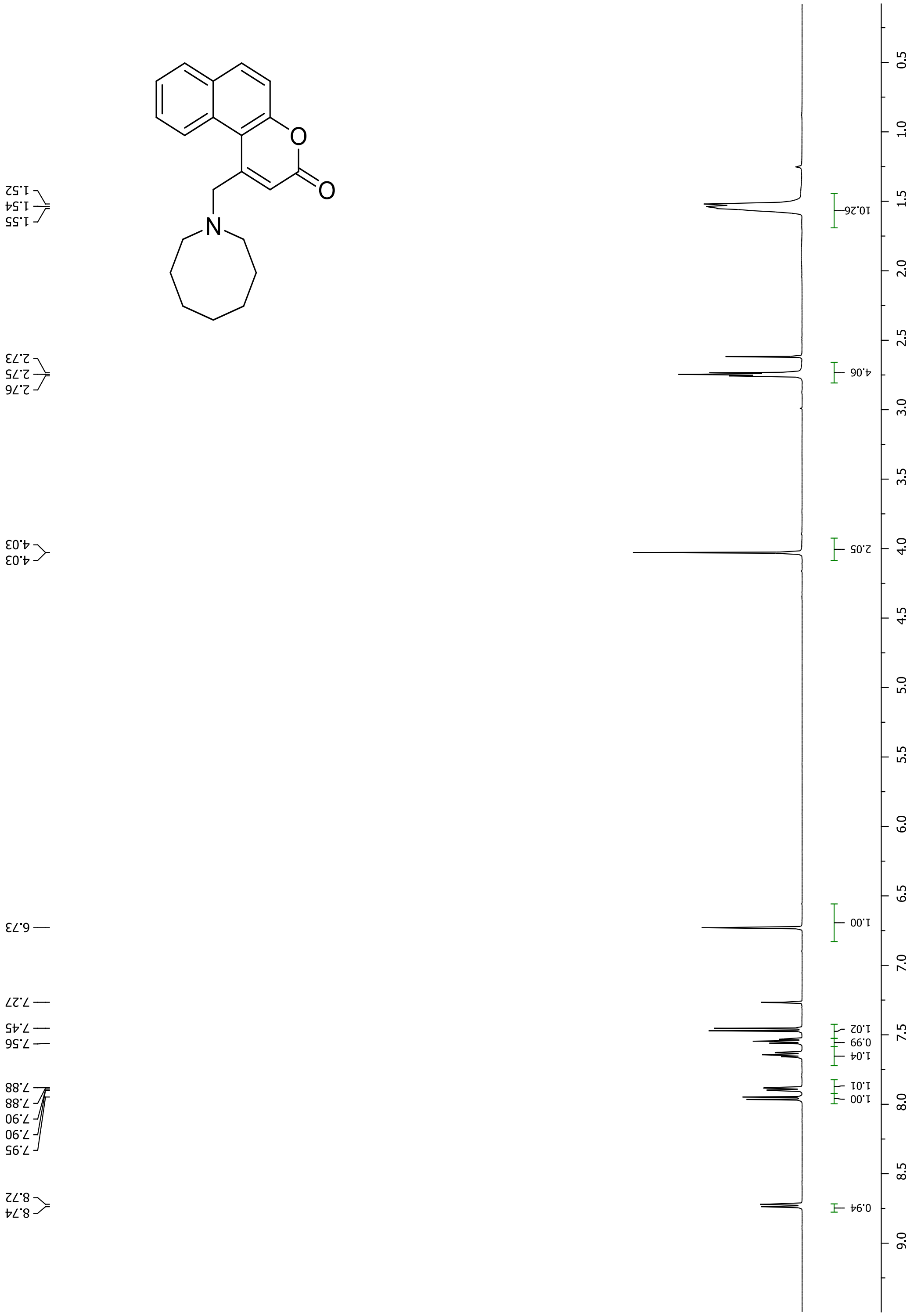
O
O
N
1.52
1.54
1.55
2.73
2.75
2.76
4.03
4.03
6.73
7.27
7.45
7.56
7.88
7.88
7.90
7.90
7.95
8.72
8.74
10.26
4.06
2.05
1.00
1.02
0.99
1.04
1.01
1.00
0.94
0.5
1.0
1.5
2.0
2.5
3.0
3.5
4.0
4.5
5.0
5.5
6.0
6.5
7.0
7.5
8.0
8.5
9.0

C4A14

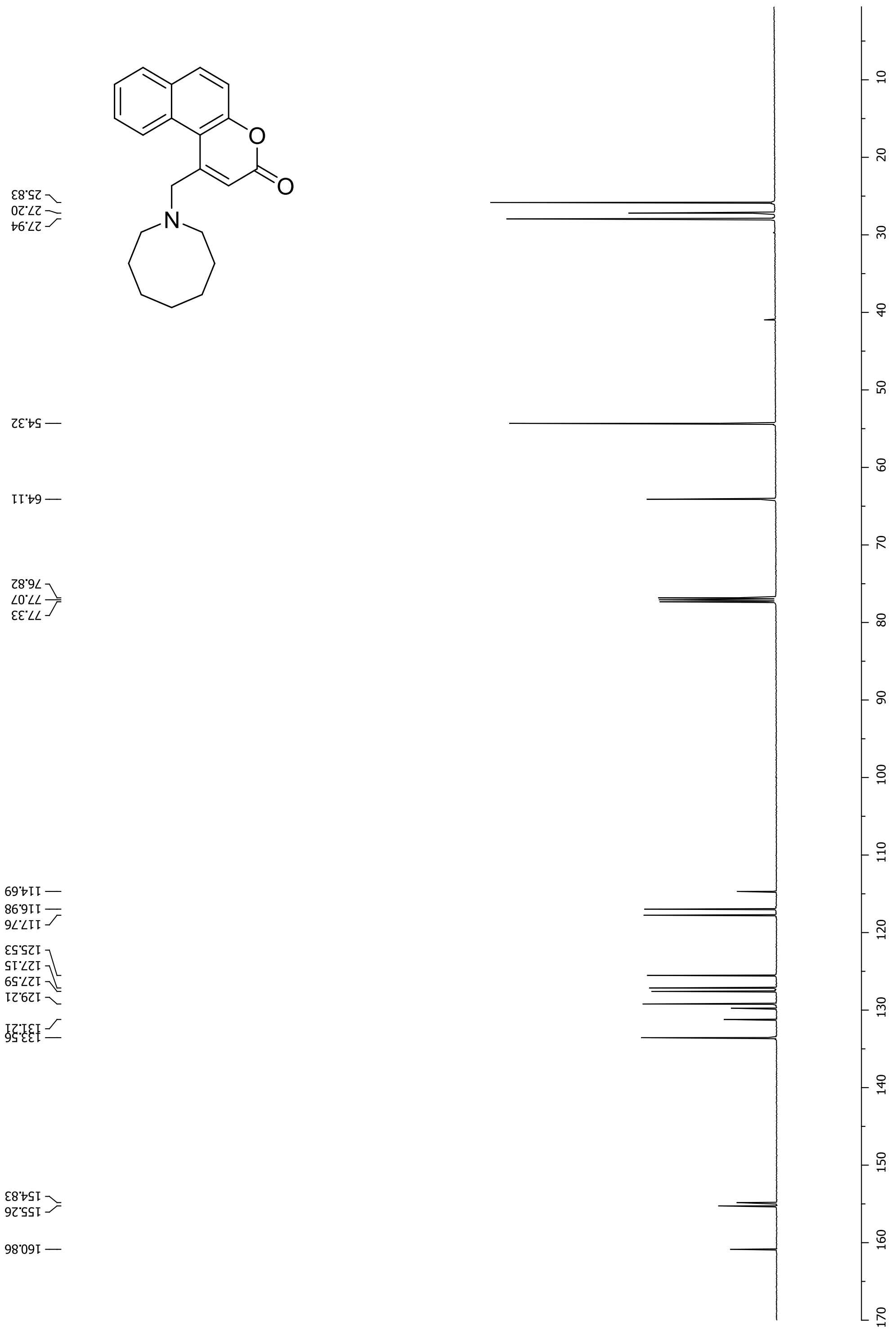

25.83
27.20
27.94
54.32
64.11
76.82
77.07
77.33
114.69
116.98
117.76
125.53
127.15
127.59
129.21
131.21
133.56
154.83
155.26
160.86
N
O
O
10 20 30 40 50 60 70 80 90 100 110 120 130 140 150 160 170

C4A16

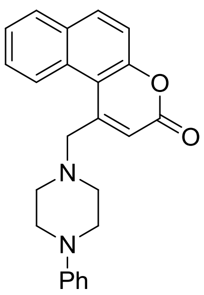
O
O
N
N
Ph

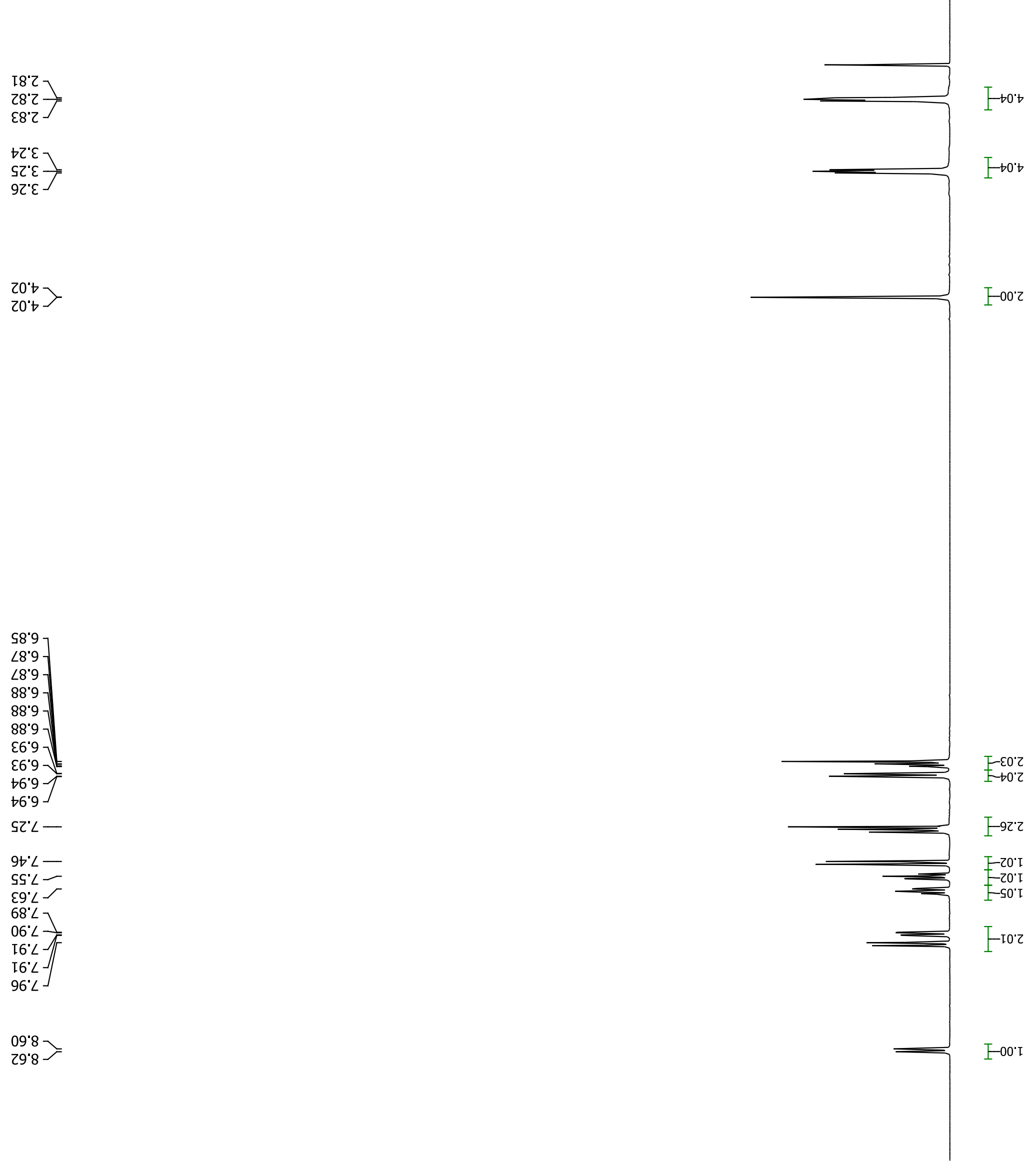
2.81
2.82
2.83
3.24
3.25
3.26
4.02
4.02
6.85
6.87
6.87
6.88
6.88
6.88
6.93
6.93
6.94
6.94
7.25
7.46
7.55
7.63
7.89
7.90
7.91
7.91
7.96
8.60
8.62
4.04
4.04
2.00
2.03
2.04
2.26
1.02
1.02
1.05
2.01
1.00

C4A16

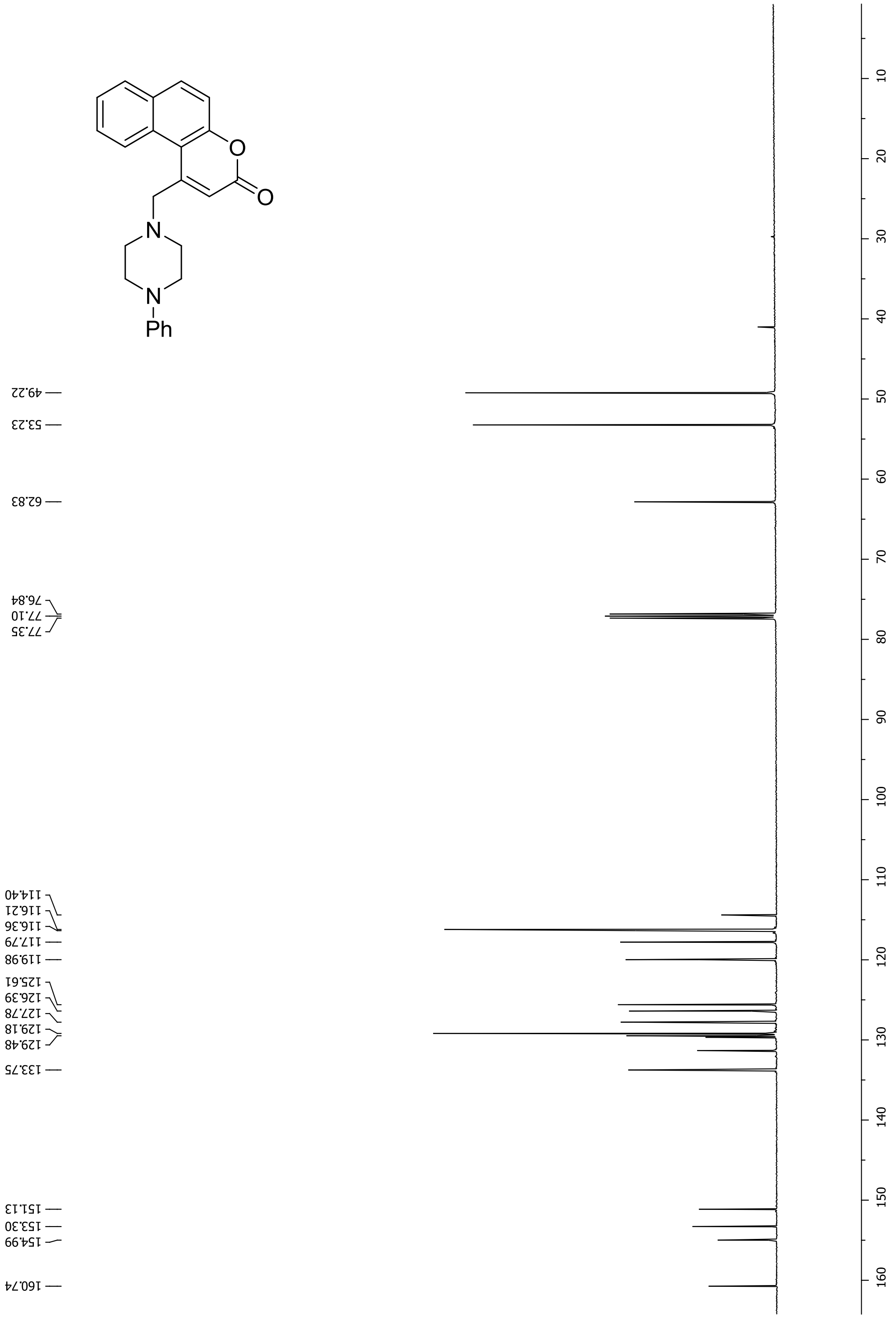

O
O
N
N
Ph
49.22
53.23
62.83
76.84
77.10
77.35
114.40
116.21
116.36
117.79
119.98
125.61
126.39
127.78
129.18
129.48
133.75
151.13
153.30
154.99
160.74
10
20
30
40
50
60
70
80
90
100
110
120
130
140
150
160

C5A11

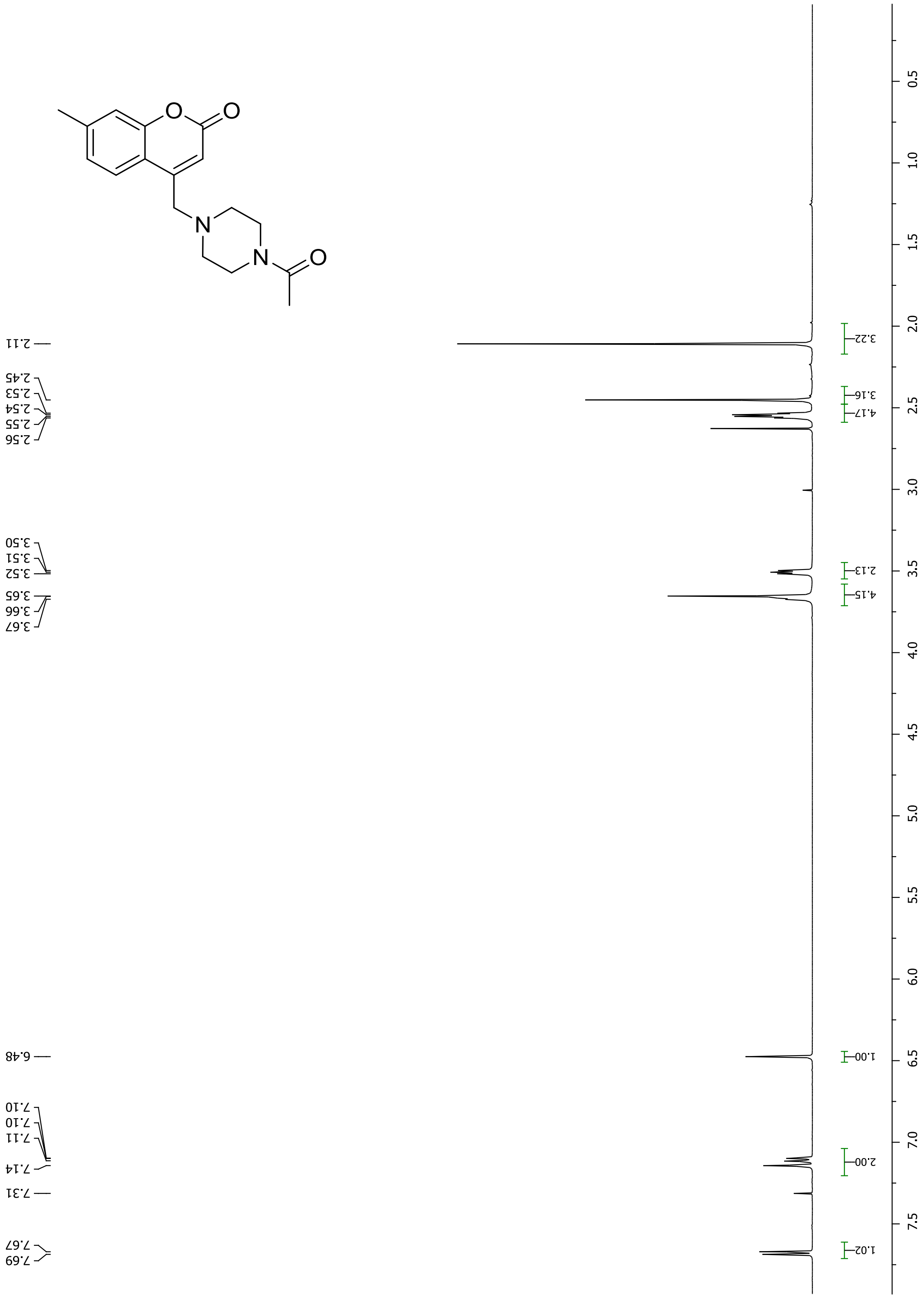
O
O
N
N
O
2.11
2.45
2.53
2.54
2.55
2.56
3.50
3.51
3.52
3.65
3.66
3.67
6.48
7.10
7.10
7.11
7.14
7.31
7.67
7.69
3.22
3.16
4.17
2.13
4.15
1.00
2.00
1.02
0.5
1.0
1.5
2.0
2.5
3.0
3.5
4.0
4.5
5.0
5.5
6.0
6.5
7.0
7.5

C5A11

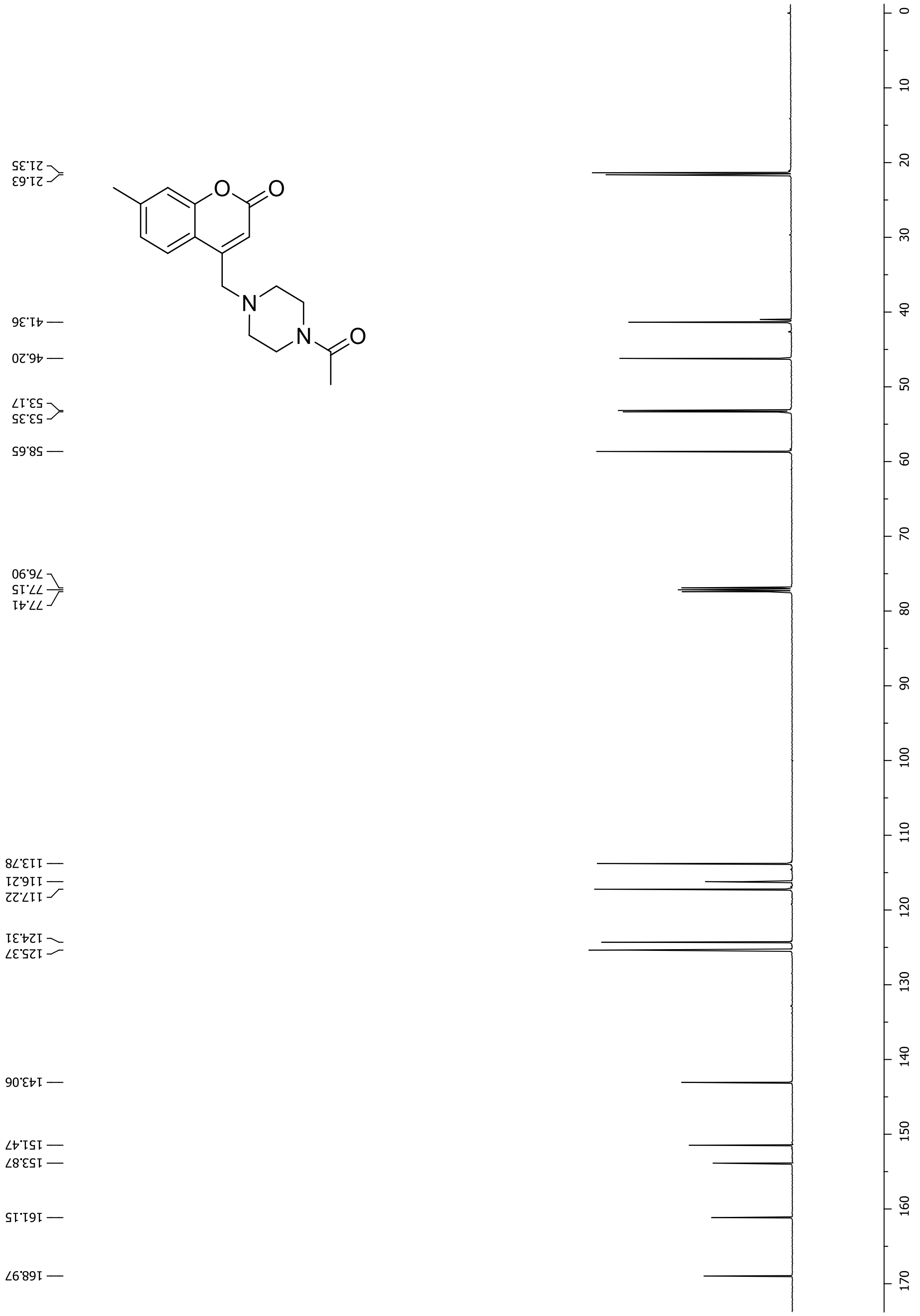

21.35
21.63
41.36
46.20
53.17
53.35
58.65
76.90
77.15
77.41
113.78
116.21
117.22
124.31
125.37
143.06
151.47
153.87
161.15
168.97
O
O
N
N
O
0
10
20
30
40
50
60
70
80
90
100
110
120
130
140
150
160
170

C6A4

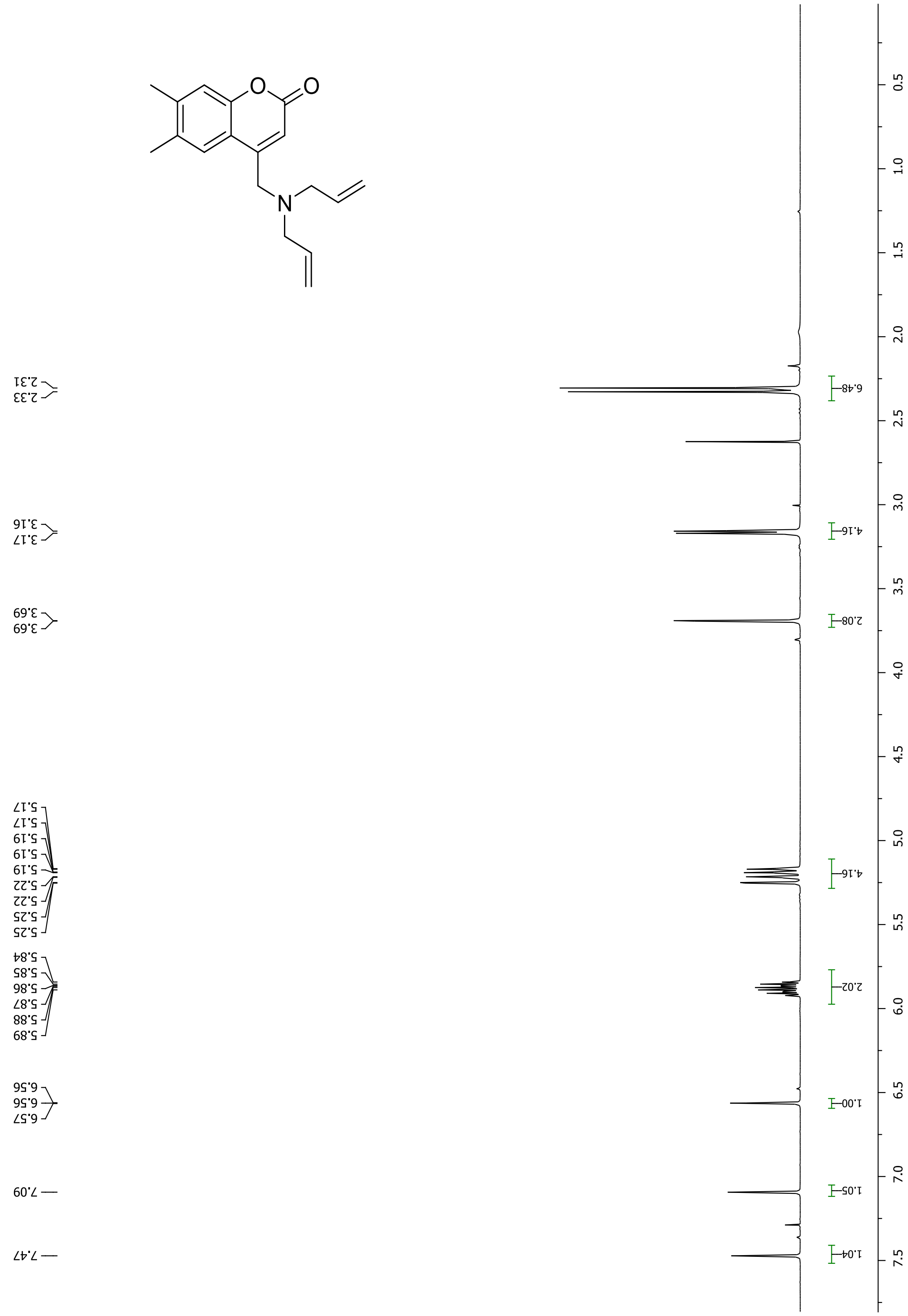
N
O
O
2.31
2.33
3.16
3.17
3.69
3.69
5.17
5.17
5.19
5.19
5.19
5.22
5.22
5.25
5.25
5.84
5.85
5.86
5.87
5.88
5.89
6.56
6.56
6.57
7.09
7.47
6.48
4.16
2.08
4.16
2.02
1.00
1.05
1.04

C6A4

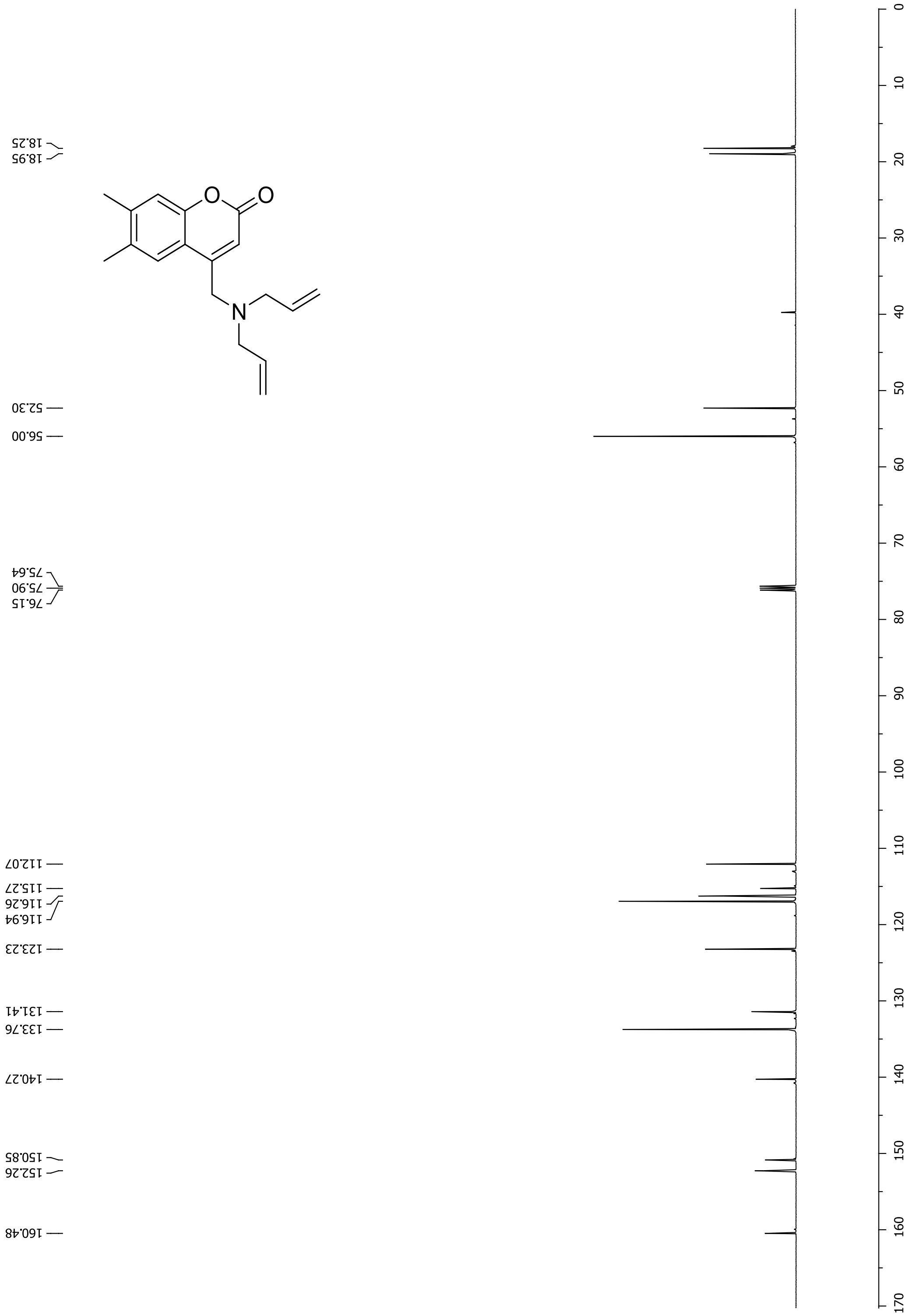

18.25
18.95
52.30
56.00
75.64
75.90
76.15
112.07
115.27
116.26
116.94
123.23
131.41
133.76
140.27
150.85
152.26
160.48
0 10 20 30 40 50 60 70 80 90 100 110 120 130 140 150 160 170

C6A6

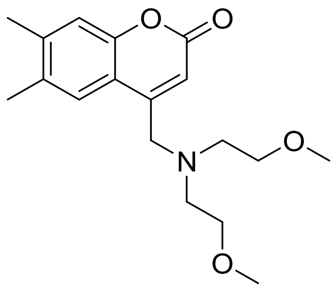

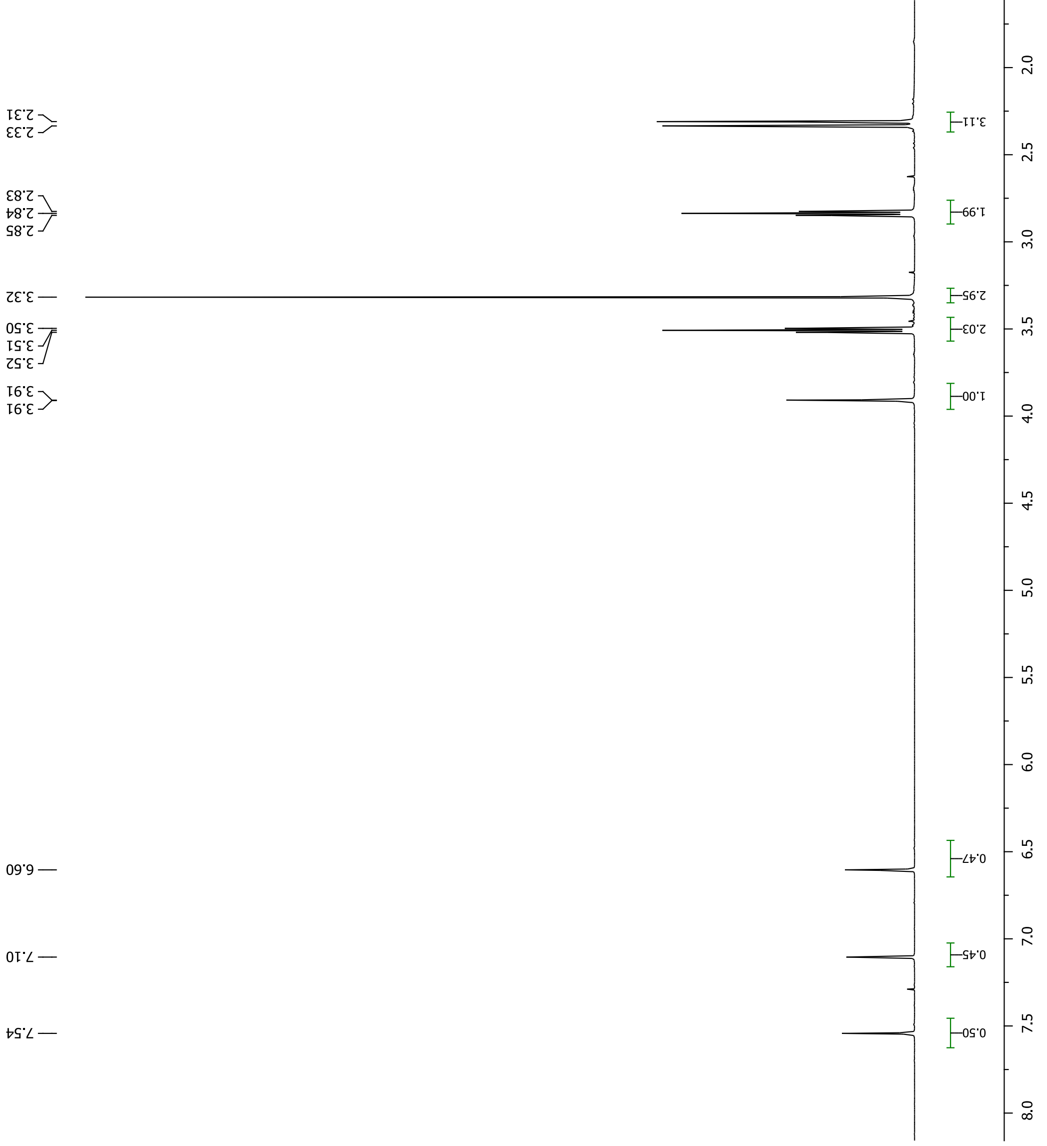
2.31
2.33
2.83
2.84
2.85
3.32
3.50
3.51
3.52
3.91
3.91
6.60
7.10
7.54
3.11
1.99
2.95
2.03
1.00
0.47
0.45
0.50
0.0
0.5
1.0
1.5
2.0
2.5
3.0
3.5
4.0
4.5
5.0
5.5
6.0
6.5
7.0
7.5
8.0

C6A6

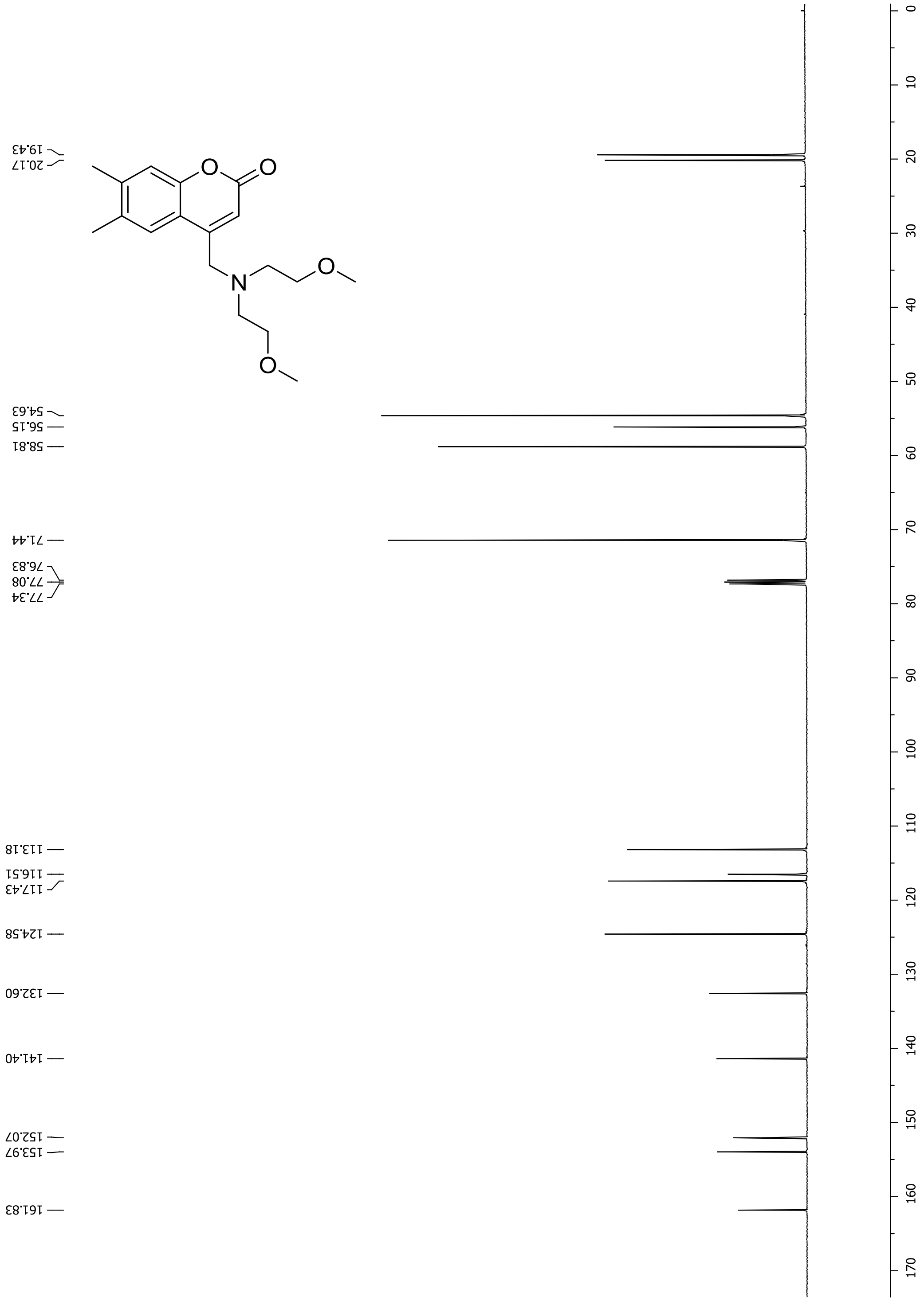

19.43
20.17
54.63
56.15
58.81
71.44
76.83
77.08
77.34
113.18
116.51
117.43
124.58
132.60
141.40
152.07
153.97
161.83
0
10
20
30
40
50
60
70
80
90
100
110
120
130
140
150
160
170

C6A13

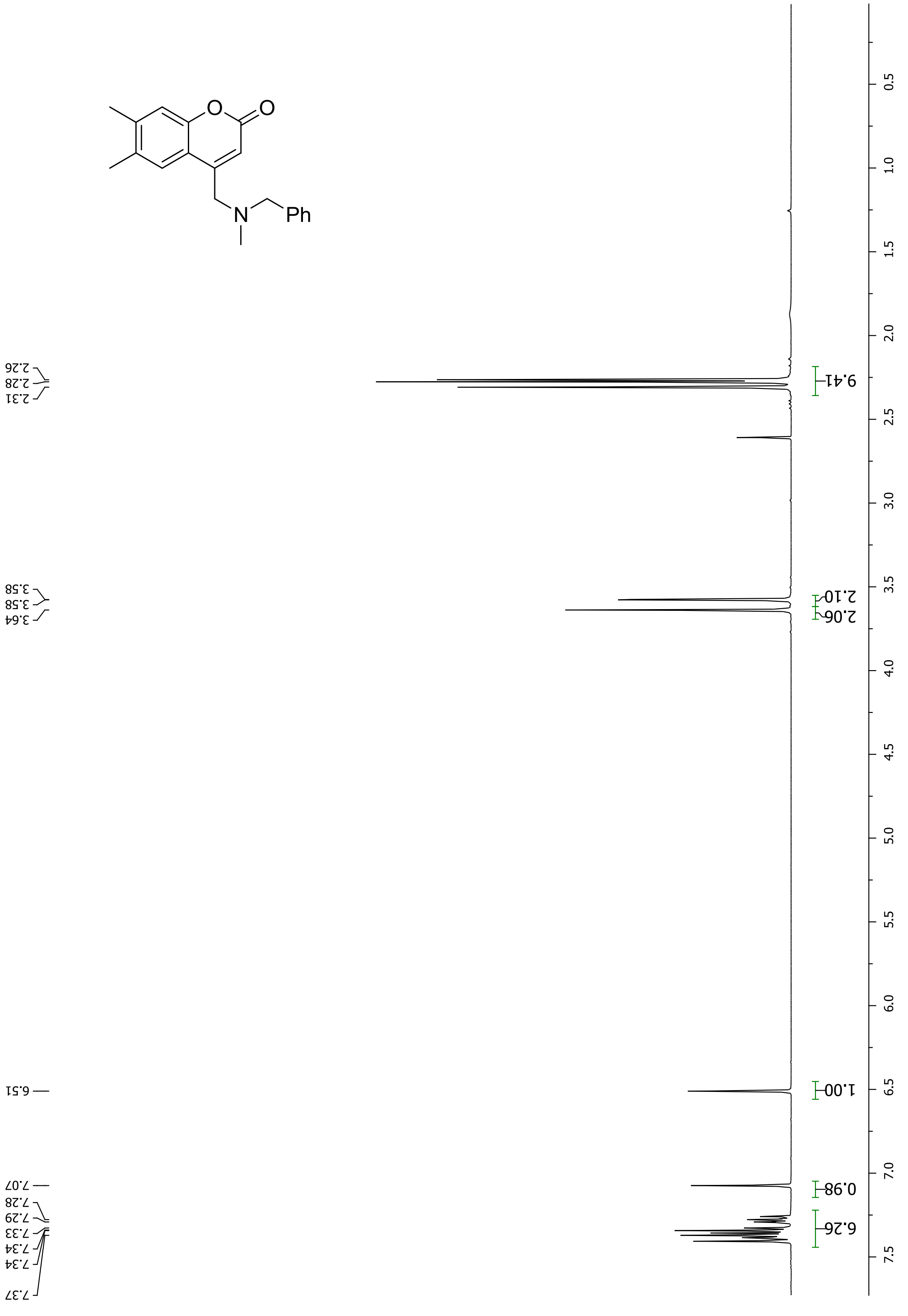
O
O
N
Ph
2.26
2.28
2.31
3.58
3.58
3.64
6.51
7.07
7.28
7.29
7.33
7.34
7.34
7.37
9.41
2.10
2.06
1.00
0.98
6.26

C6A13

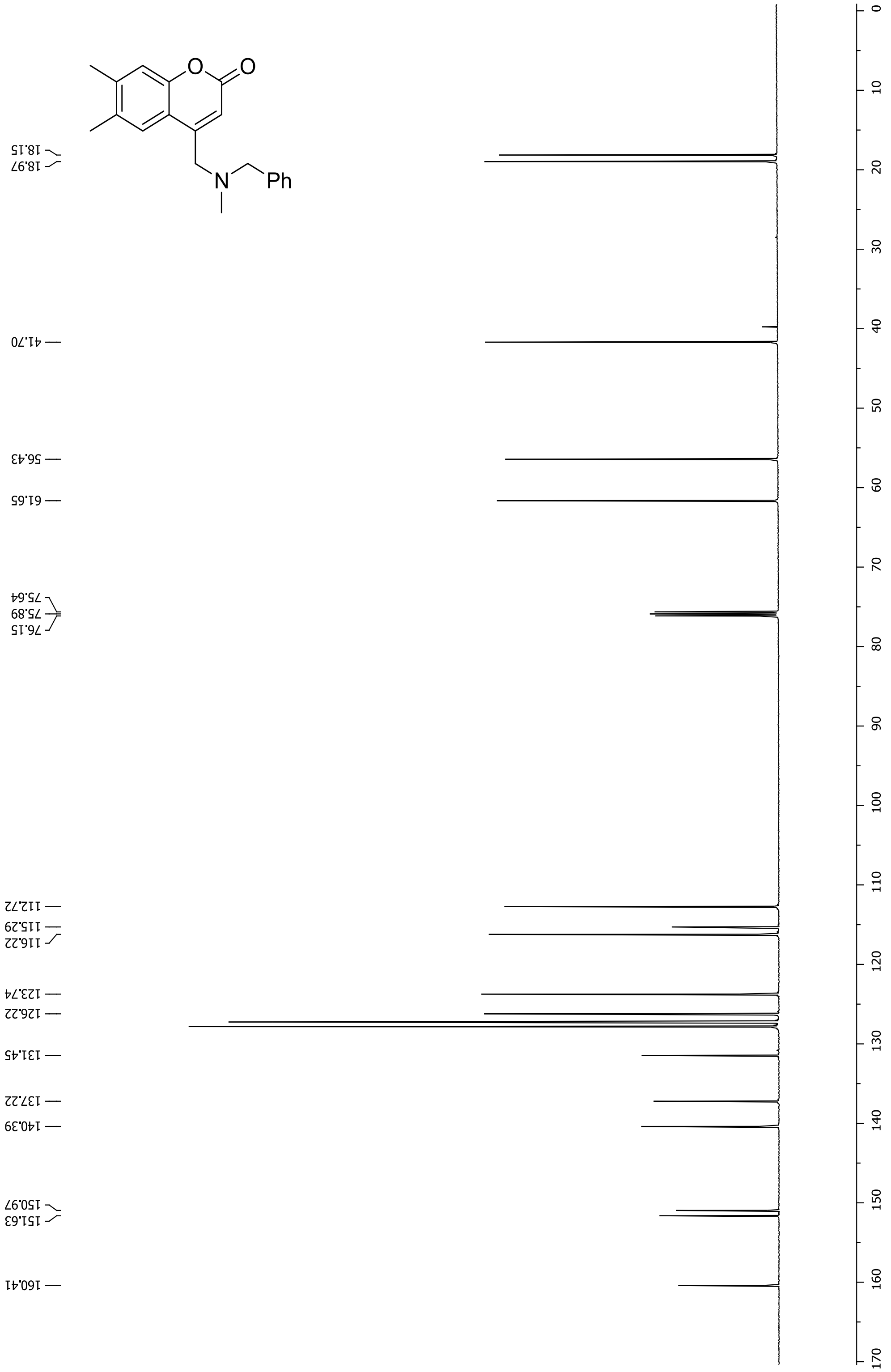
Ph
18.15
18.97
41.70
56.43
61.65
75.64
75.89
76.15
112.72
115.29
116.22
123.74
126.22
131.45
137.22
140.39
150.97
151.63
160.41

C7A9

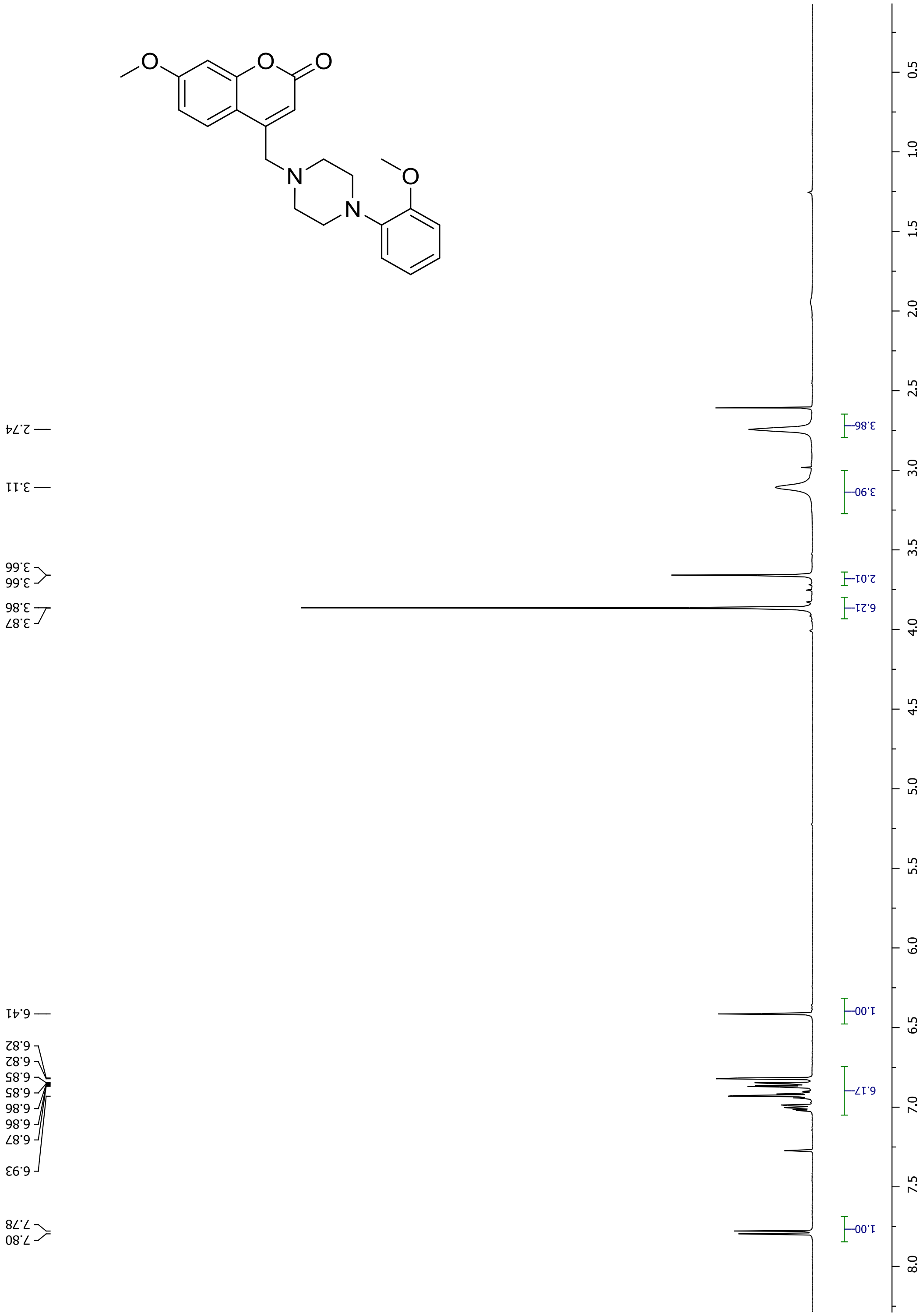
O
O
O
N
N
O
2.74
3.11
3.66
3.66
3.86
3.87
6.41
6.82
6.82
6.85
6.85
6.86
6.86
6.87
6.93
7.78
7.80
3.86
3.90
2.01
6.21
1.00
6.17
1.00
0.5
1.0
1.5
2.0
2.5
3.0
3.5
4.0
4.5
5.0
5.5
6.0
6.5
7.0
7.5
8.0

C7A9

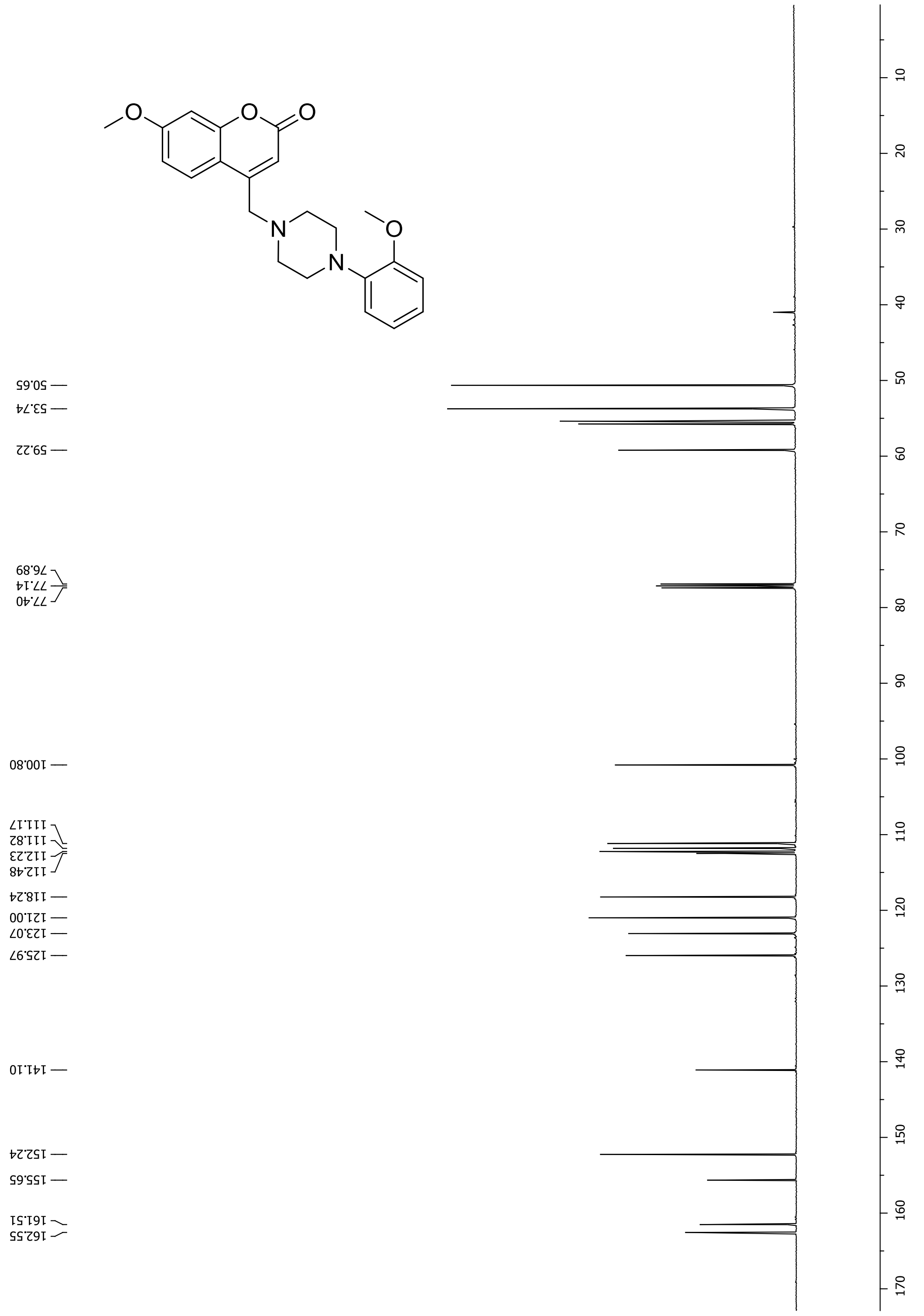

50.65
53.74
59.22
76.89
77.14
77.40
100.80
111.17
111.82
112.23
112.48
118.24
121.00
123.07
125.97
141.10
152.24
155.65
161.51
162.55

C7A11

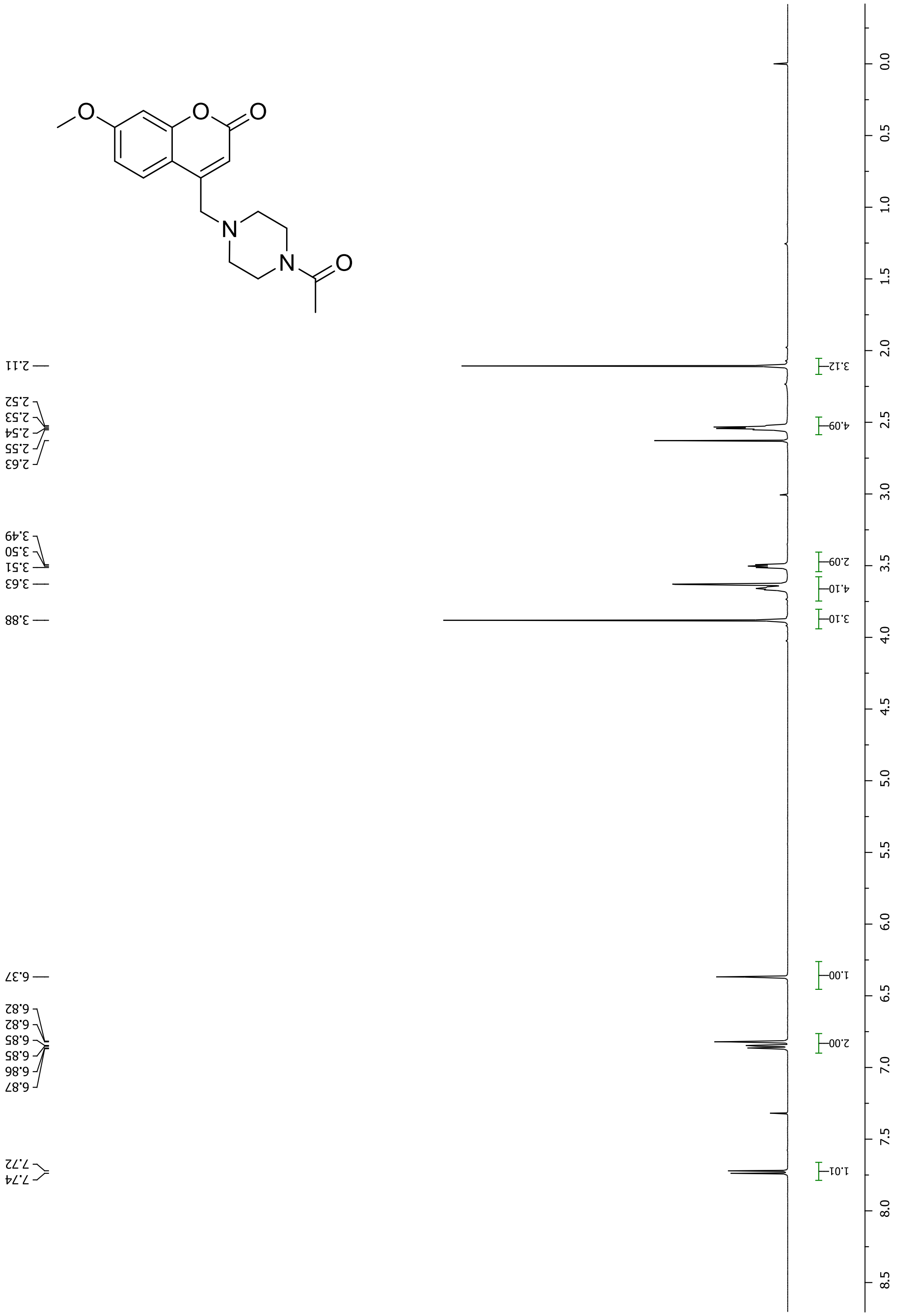

C7A11

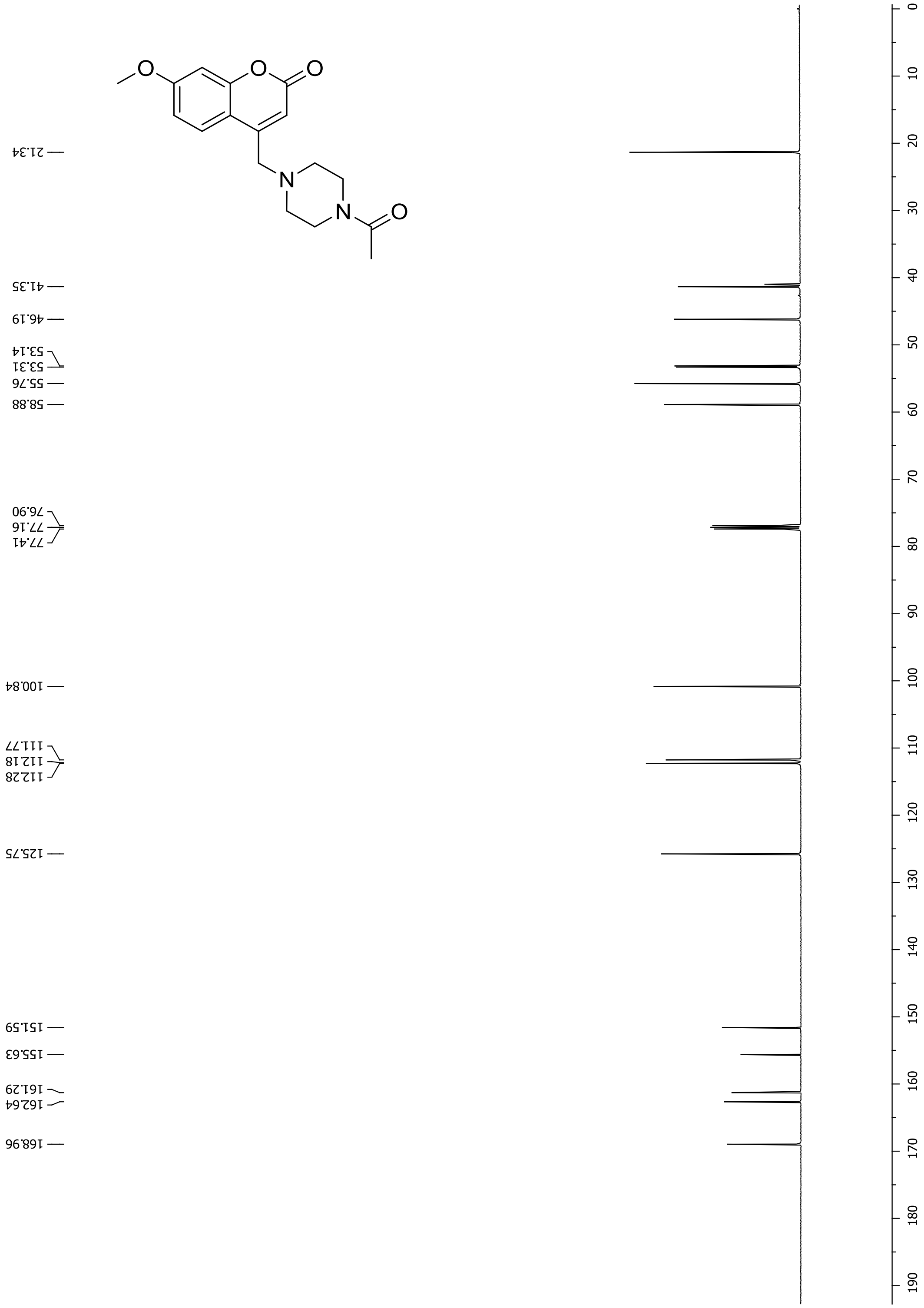
21.34
41.35
46.19
53.14
53.31
55.76
58.88
76.90
77.16
77.41
100.84
111.77
112.18
112.28
125.75
151.59
155.63
161.29
162.64
168.96
0
10
20
30
40
50
60
70
80
90
100
110
120
130
140
150
160
170
180
190

C7A13

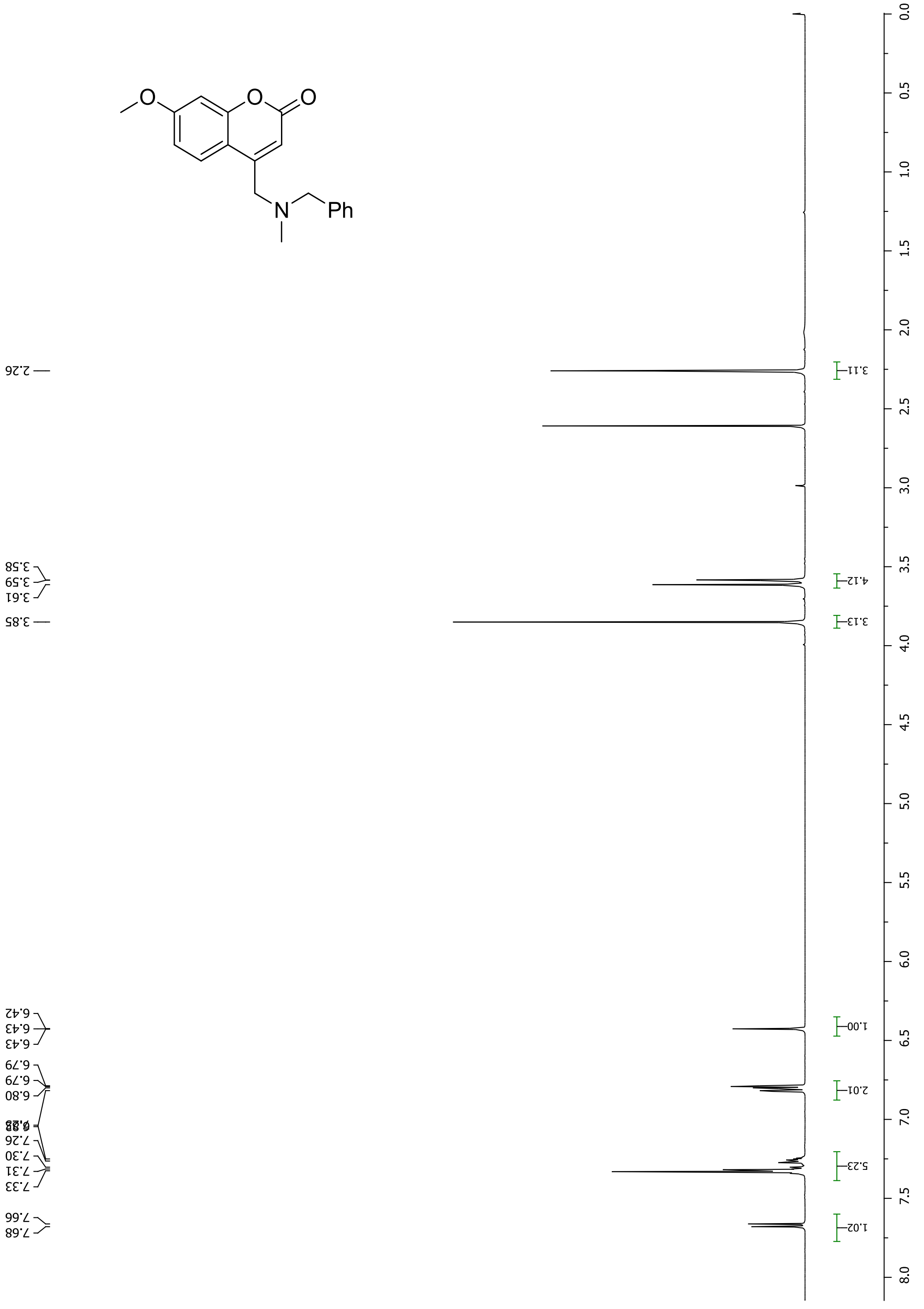

O
O
O
N
Ph
2.26
3.58
3.59
3.61
3.85
6.42
6.43
6.43
6.79
6.79
6.80
7.26
7.30
7.31
7.33
7.66
7.68
3.11
4.12
3.13
1.00
2.01
5.23
1.02
0.0
0.5
1.0
1.5
2.0
2.5
3.0
3.5
4.0
4.5
5.0
5.5
6.0
6.5
7.0
7.5
8.0

C7A13

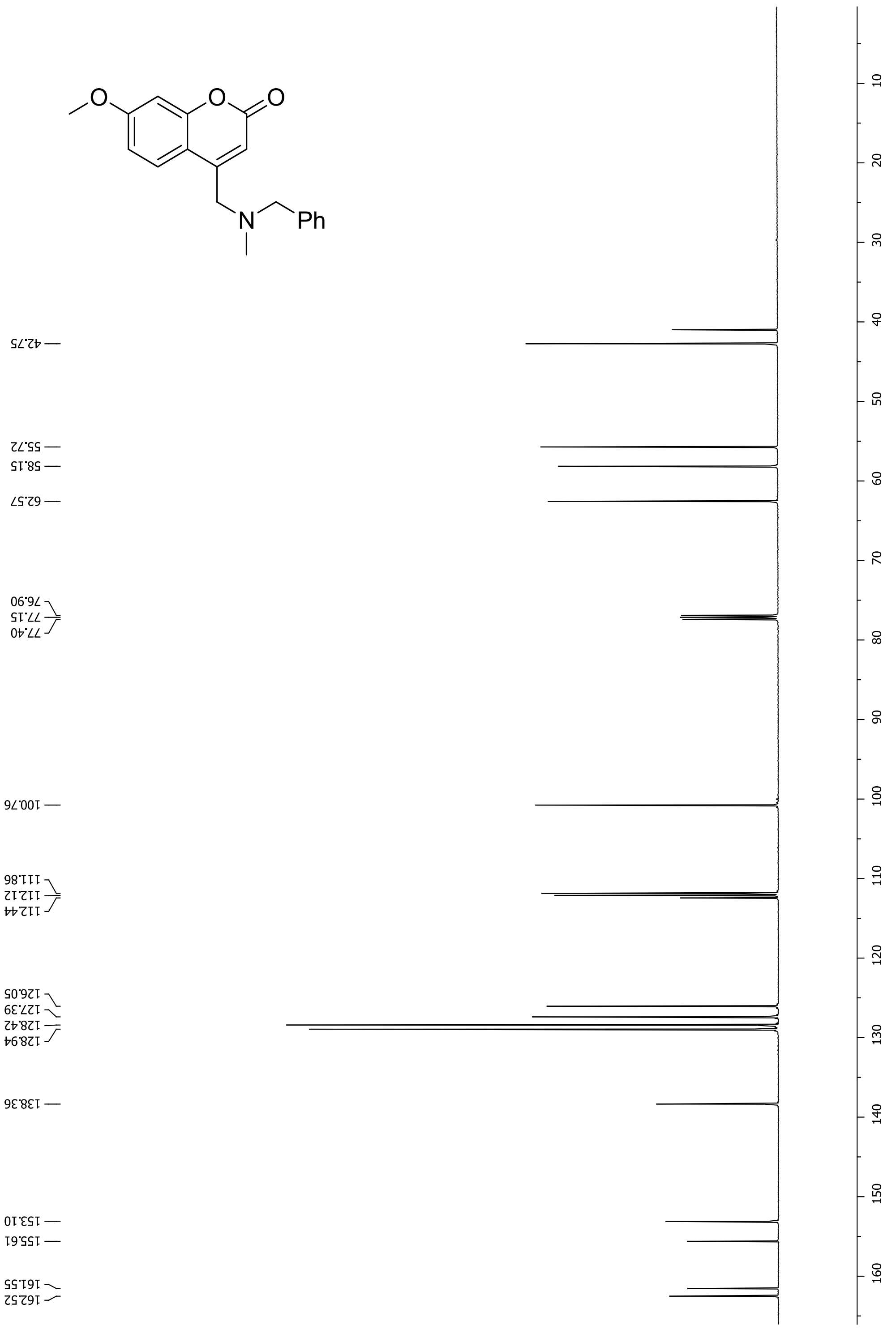

O
O
O
N
Ph
42.75
55.72
58.15
62.57
76.90
77.15
77.40
100.76
111.86
112.12
112.44
126.05
127.39
128.42
128.94
138.36
153.10
155.61
161.55
162.52
10
20
30
40
50
60
70
80
90
100
110
120
130
140
150
160

C8A3

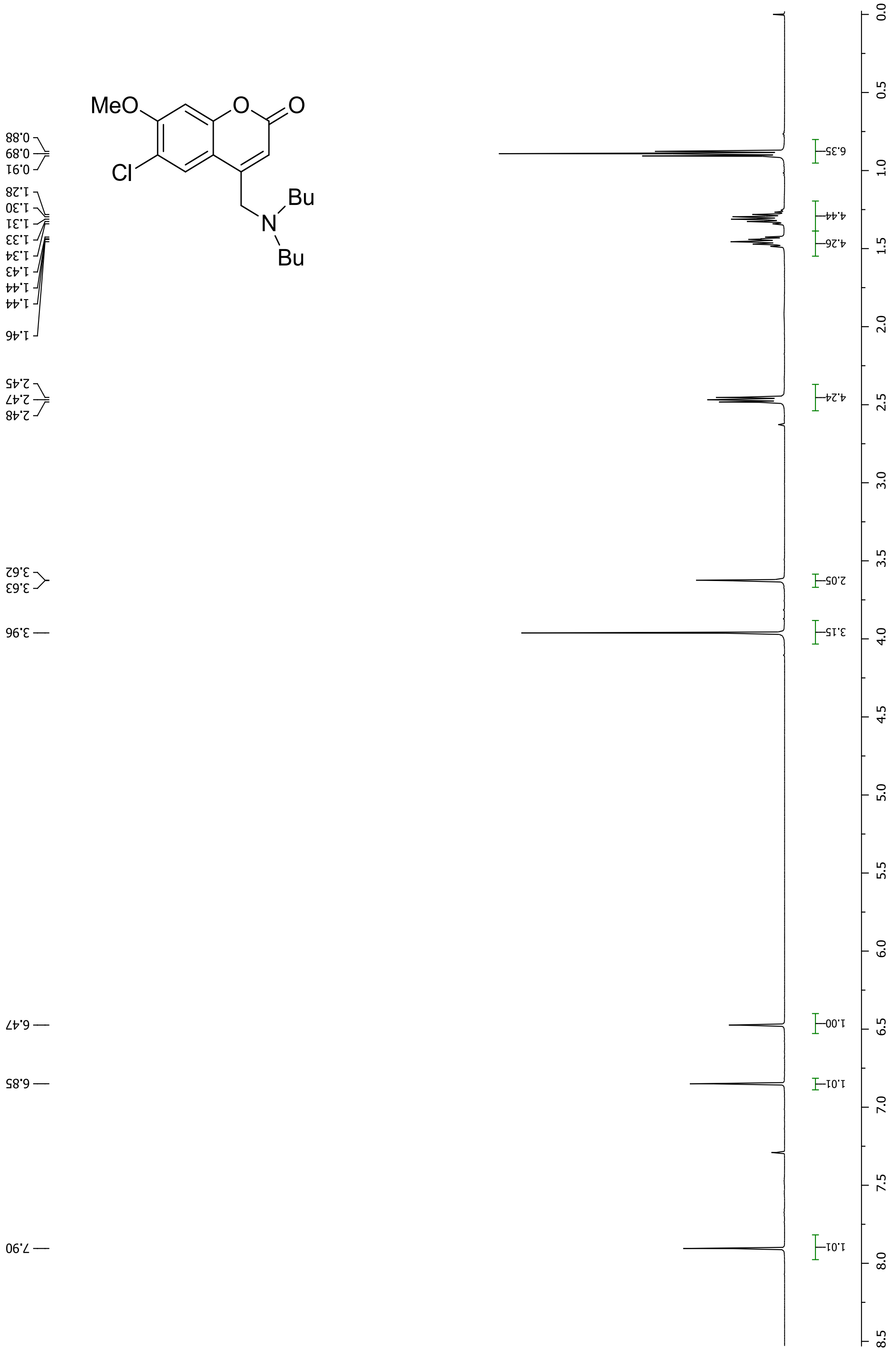
MeO
O
O
Cl
N
Bu
Bu
0.88
0.89
0.91
1.28
1.30
1.31
1.33
1.34
1.43
1.44
1.44
1.46
2.45
2.47
2.48
3.62
3.63
3.96
6.47
6.85
7.90
6.35
4.44
4.26
4.24
2.05
3.15
1.00
1.01
1.01
0.0
0.5
1.0
1.5
2.0
2.5
3.0
3.5
4.0
4.5
5.0
5.5
6.0
6.5
7.0
7.5
8.0
8.5

C8A3

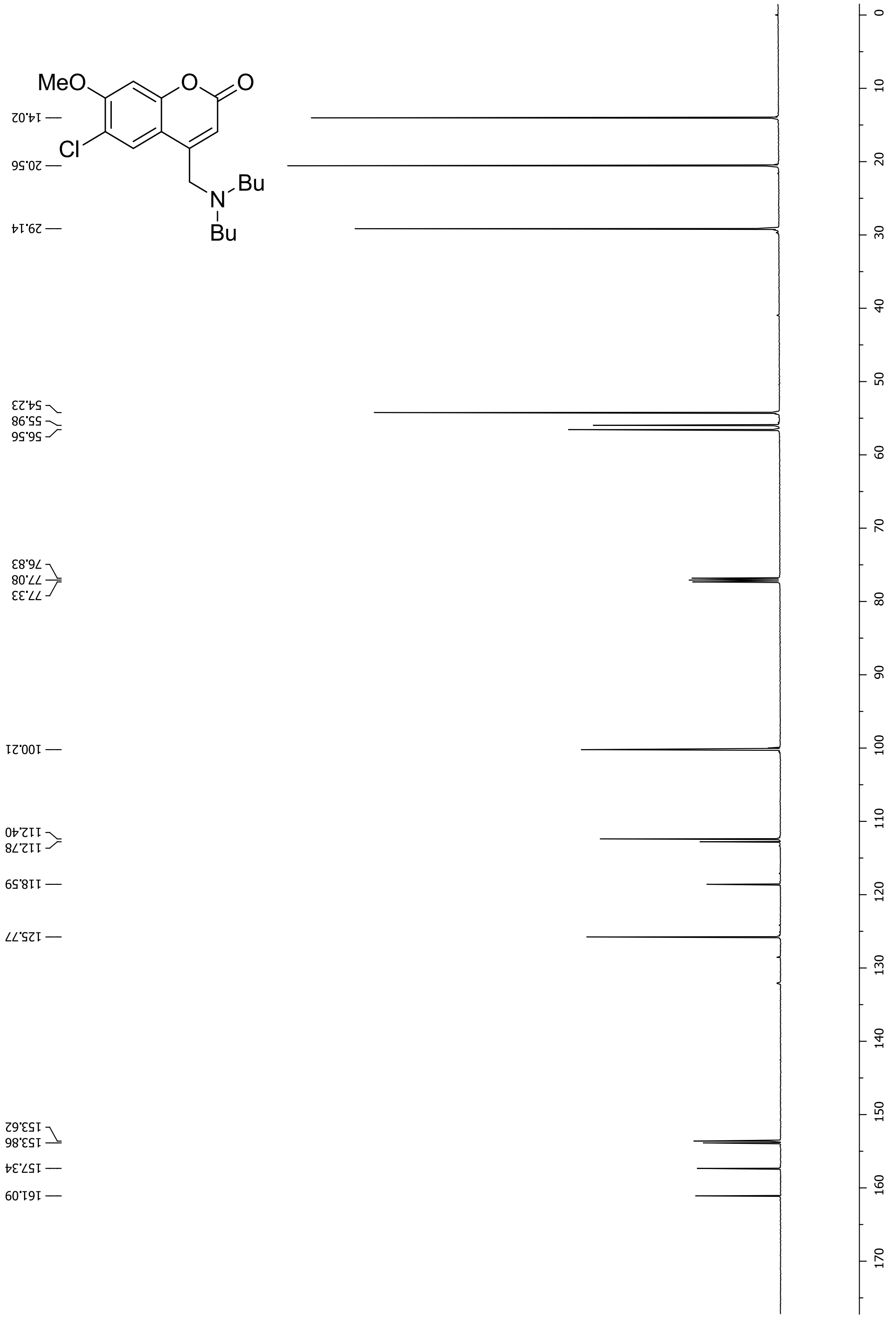

MeO
O
O
Cl
N
Bu
Bu
14.02
20.56
29.14
54.23
55.98
56.56
76.83
77.08
77.33
100.21
112.40
112.78
118.59
125.77
153.62
153.86
157.34
161.09
0
10
20
30
40
50
60
70
80
90
100
110
120
130
140
150
160
170

C8A12

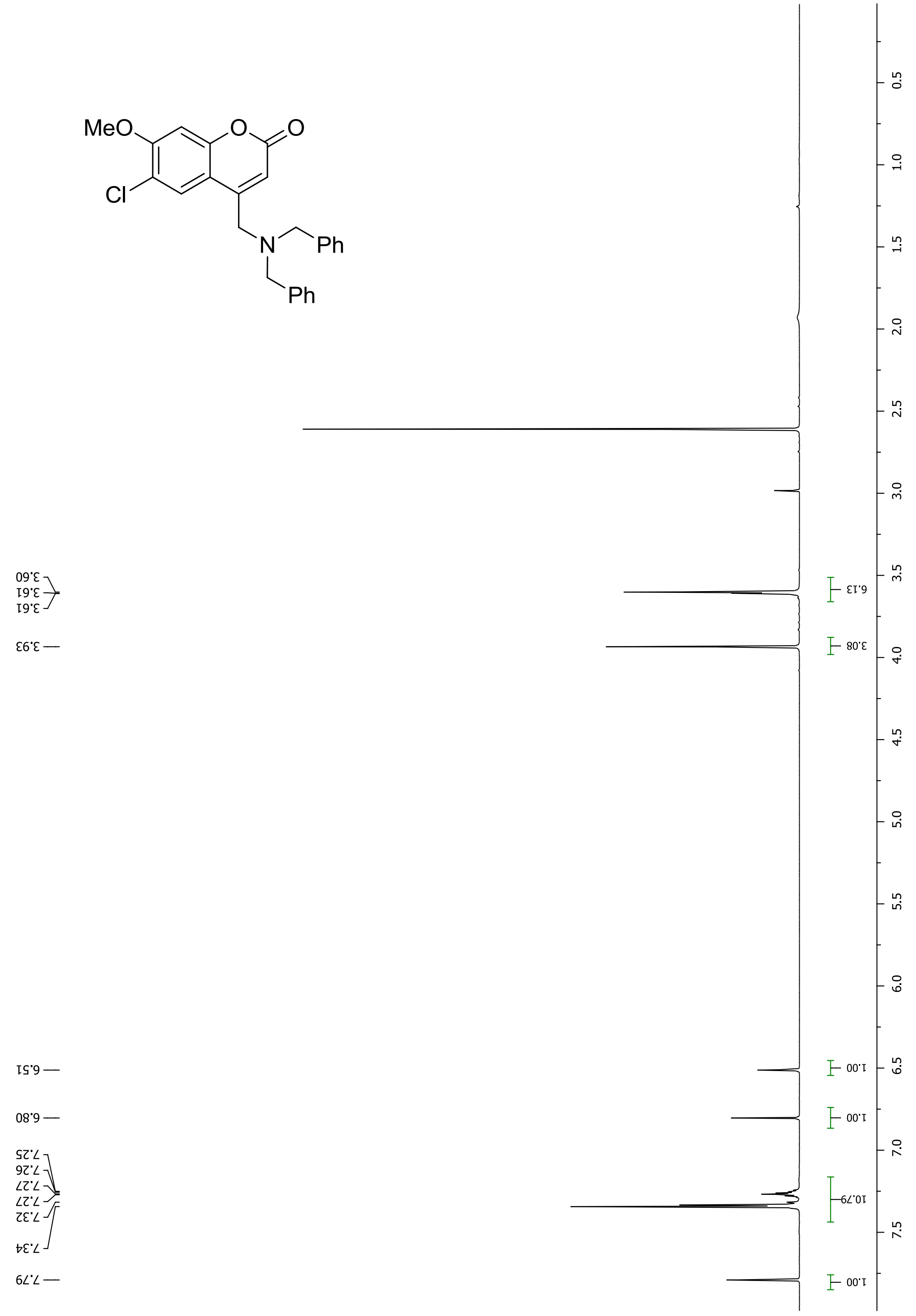
MeO
O
O
Cl
N
Ph
Ph
3.60
3.61
3.61
3.93
6.51
6.80
7.25
7.26
7.27
7.27
7.32
7.34
7.79
6.13
3.08
1.00
1.00
10.79
1.00
0.5
1.0
1.5
2.0
2.5
3.0
3.5
4.0
4.5
5.0
5.5
6.0
6.5
7.0
7.5

C8A12

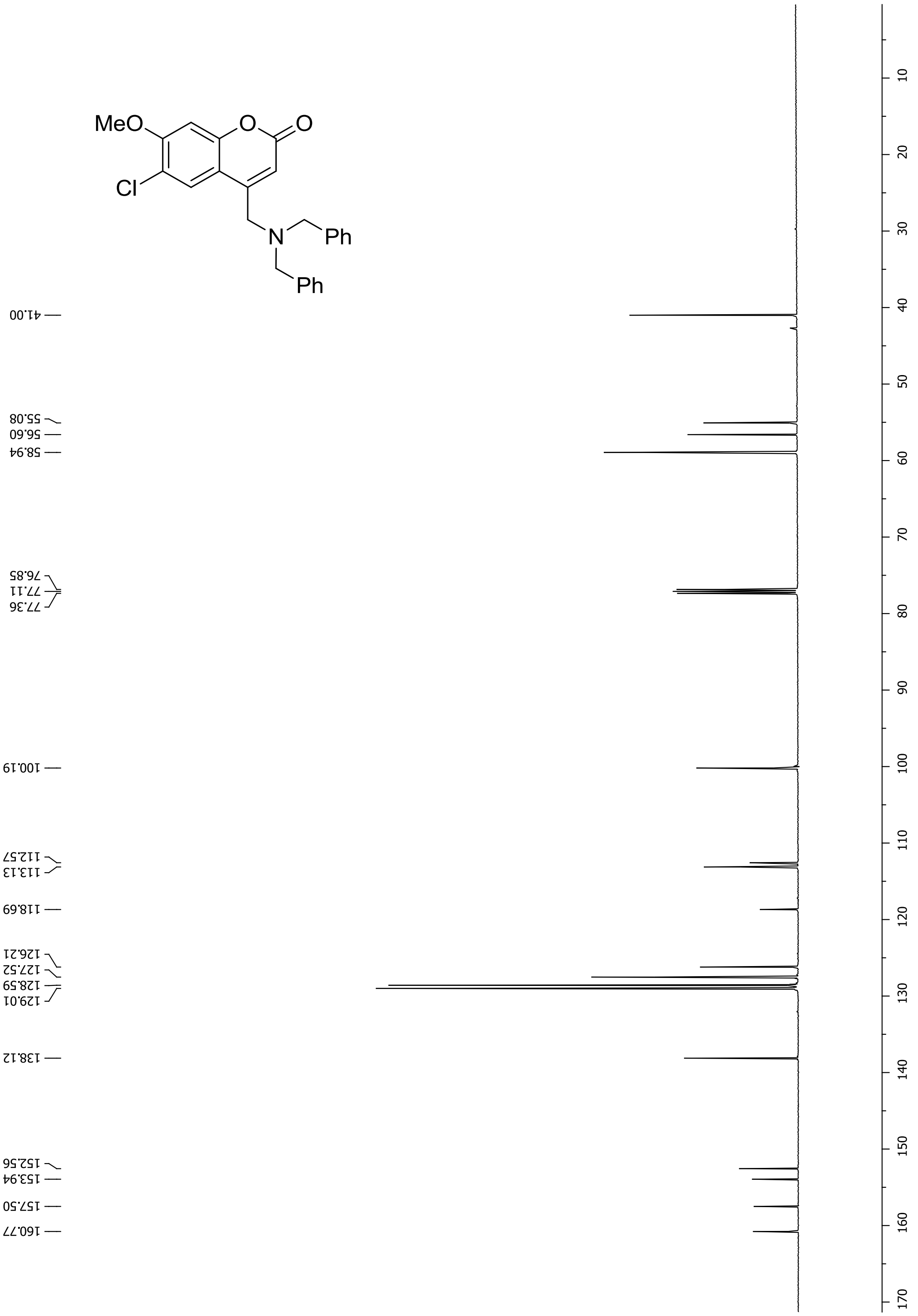
MeO
O
O
Cl
N
Ph
Ph
41.00
55.08
56.60
58.94
76.85
77.11
77.36
100.19
112.57
113.13
118.69
126.21
127.52
128.59
129.01
138.12
152.56
153.94
157.50
160.77
10
20
30
40
50
60
70
80
90
100
110
120
130
140
150
160
170

C8A14

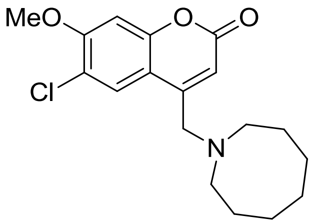
MeO
O
O
Cl
N

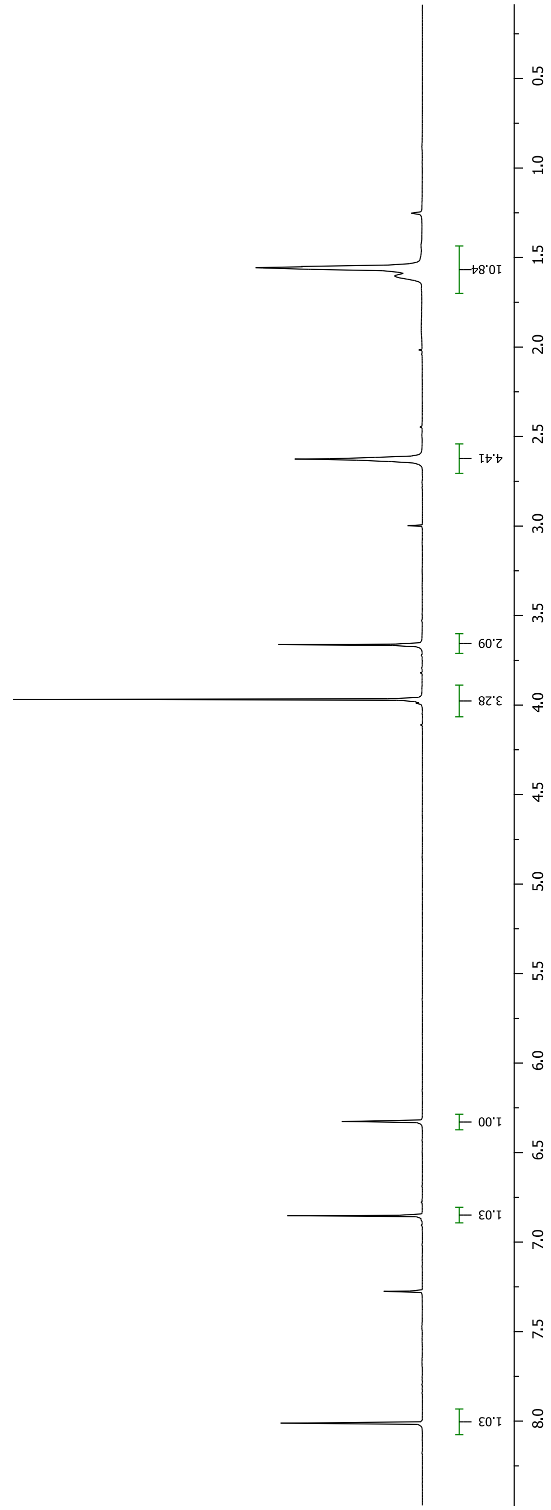
1.55
1.56
1.56
1.60
1.61
2.63
3.66
3.66
3.97
6.33
6.85
7.27
8.01
10.84
4.41
2.09
3.28
1.00
1.03
1.03
0.5
1.0
1.5
2.0
2.5
3.0
3.5
4.0
4.5
5.0
5.5
6.0
6.5
7.0
7.5
8.0

C8A14

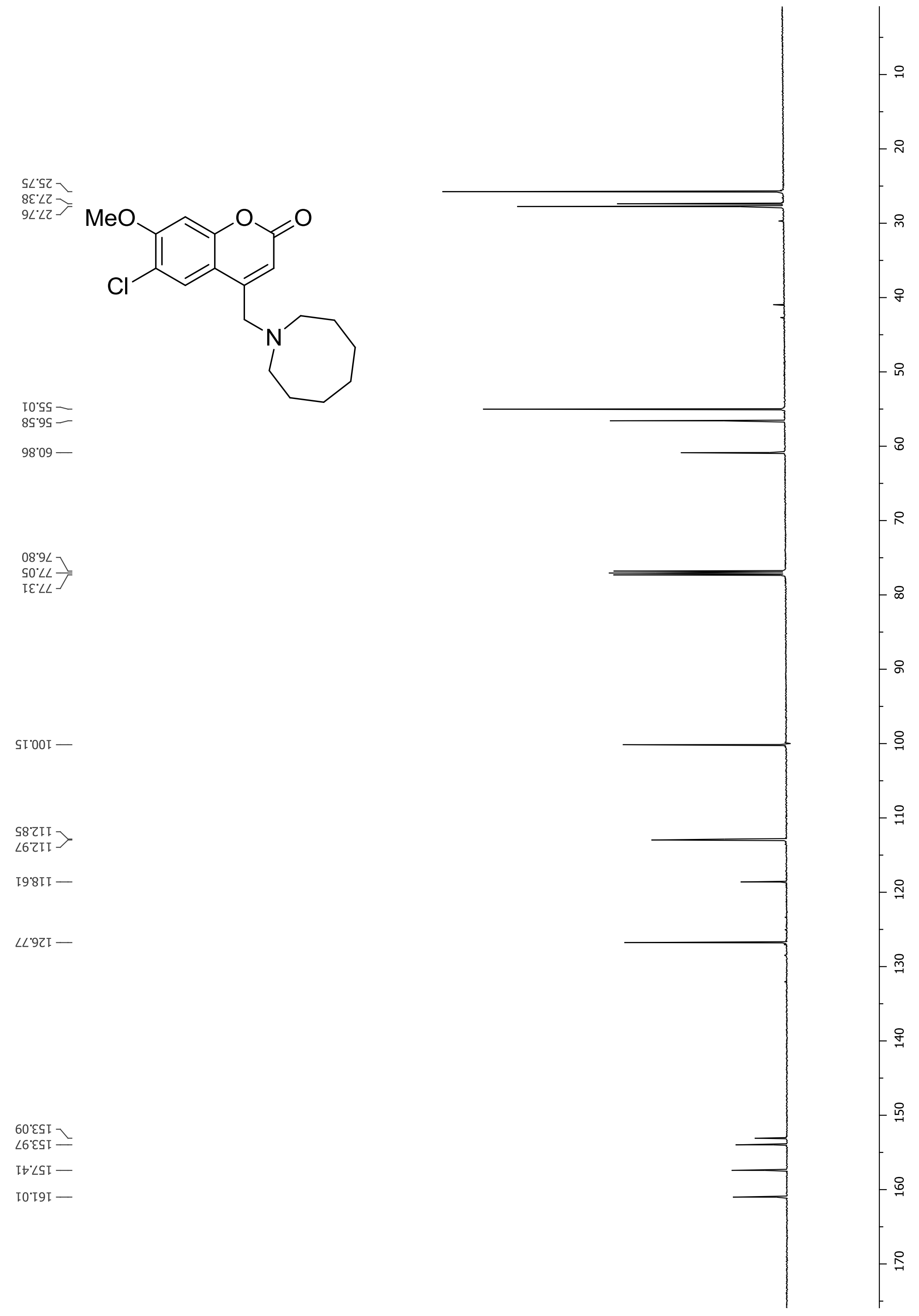

25.75
27.38
27.76
55.01
56.58
60.86
76.80
77.05
77.31
100.15
112.85
112.97
118.61
126.77
153.09
153.97
157.41
161.01
MeO
O
O
Cl
N
10
20
30
40
50
60
70
80
90
100
110
120
130
140
150
160
170
