# Supplementary material for: Palladium-catalyzed substitution of (coumarinyl)methyl acetates with C-, N-, and S-nucleophiles
Source: Beilstein J Org Chem. 2012 Jul 27;8:1200–7. doi: 10.3762/bjoc.8.133 (PMC3458738; doi:10.3762/bjoc.8.133)
Supplement: File 2 — Detailed results of the library analysis are likewise included. [file Beilstein_J_Org_Chem-08-1200-s002.pdf]

Supporting Information

for

Palladium-catalyzed substitution of (coumarinyl)methyl acetates with C-, N-, and S-nucleophiles

Kalicharan Chattopadhyay[1,2], Erik Fenster[2], Alexander J. Grenning[1] and Jon A. Tunge[1,2*]

[1]Department of Chemistry, University of Kansas, 1251 Wescoe Hall Drive, Lawrence, KS 66045-7528, USA and [2]KU Chemical Methodologies and Library Development Center of Excellence, University of Kansas, 1501 Wakarusa Drive, Lawrence, Kansas 66047, USA.

Email: Jon A. Tunge - tunge@ku.edu

**Corresponding author*

Detailed results of the library analysis are likewise included.

Compound Lot Number (Notebook Number)	CCT Tube Barcode Number	STRUCTURE	Molecular Weight	Theoretical yield (mg)	Actual yield (mg)	Actual yield (%)	Purity (%)
CMC-50-181-C1A1	CCT117707	$C_{14}H_{15}NO_3$	245,2738	85,8	50,7	59	100,0
CMC-50-181-C1A10	CCT117804	$C_{20}H_{18}Cl_2N_2O_2$	389,27512	136,2	67,9	50	98,5
CMC-50-181-C1A11	CCT117790	$C_{16}H_{18}N_2O_3$	286,32572	100,2	12,8	13	100,0
CMC-50-181-C1A12	CCT117708	$C_{24}H_{21}NO_2$	355,42904	124,4	57,0	46	99,4
CMC-50-181-C1A13	CCT117711	$C_{18}H_{17}NO_2$	279,33308	97,8	55,0	56	100,0
CMC-50-181-C1A14	CCT117721	$C_{17}H_{21}NO_2$	271,35414	95,0	48,8	51	100,0
CMC-50-181-C1A15	CCT117799	$C_{14}H_{13}NO_2$	227,25852	79,5	12,2	15	0,0
CMC-50-181-C1A16	CCT117794	$C_{20}H_{20}N_2O_2$	320,385	112,1	37,9	34	100,0
CMC-50-181-C1A2	CCT117763	$C_{15}H_{17}NO_2$	243,30098	85,2	45,5	53	99,4
CMC-50-181-C1A3	CCT117795	$C_{18}H_{25}NO_2$	287,3966	100,6	36,6	36	100,0
CMC-50-181-C1A4	CCT117801	$C_{16}H_{17}NO_2$	255,31168	89,4	39,3	44	100,0
CMC-50-181-C1A5	CCT117802	$C_{14}H_{15}NO_2$	229,2744	80,2	32,4	40	97,9
CMC-50-181-C1A6	CCT117731	$C_{16}H_{21}NO_4$	291,34224	102,0	66,8	66	98,9
CMC-50-181-C1A7	CCT117768	$C_{16}H_{21}NO_2$	259,34344	90,8	47,6	52	100,0
CMC-50-181-C1A8	CCT117709	$C_{20}H_{19}FN_2O_2$	338,3754632	118,4	45,8	39	98,4
CMC-50-181-C1A9	CCT117766	$C_{21}H_{22}N_2O_3$	350,41098	122,6	61,1	50	100,0
CMC-50-181-C2A1	CCT117786	$C_{15}H_{16}ClNO_3$	293,74544	102,8	20,9	20	100,0
CMC-50-181-C2A10	CCT117780	$C_{21}H_{19}Cl_3N_2O_2$	437,74676	153,2	25,1	16	100,0
CMC-50-181-C2A11	CCT117770	$C_{17}H_{19}ClN_2O_3$	334,79736	117,2		0	
CMC-50-181-C2A12	CCT117767	$C_{25}H_{22}ClNO_2$	403,90068	141,4	72,8	51	100,0
CMC-50-181-C2A13	CCT117765	$C_{19}H_{18}ClNO_2$	327,80472	114,7	80,9	71	100,0
CMC-50-181-C2A14	CCT117642	$C_{18}H_{22}ClNO_2$	319,82578	111,9	8,6	8	100,0
CMC-50-181-C2A15	CCT117682	$C_{15}H_{14}ClNO_2$	275,73016	96,5	23,4	24	0,0
CMC-50-181-C2A16	CCT117774	$C_{21}H_{21}ClN_2O_2$	368,85664	129,1	16,7	13	100,0
CMC-50-181-C2A2	CCT117749	$C_{16}H_{18}ClNO_2$	291,77262	102,1	41,1	40	100,0
CMC-50-181-C2A3	CCT117713	$C_{19}H_{26}ClNO_2$	335,86824	117,6	47,5	40	100,0
CMC-50-181-C2A4	CCT117715	$C_{17}H_{18}ClNO_2$	303,78332	106,3	57,1	54	100,0
CMC-50-181-C2A5	CCT117791	$C_{15}H_{16}ClNO_2$	277,74604	97,2	27,7	28	99,5
CMC-50-181-C2A6	CCT117798	$C_{17}H_{22}ClNO_4$	339,81388	118,9	76,3	64	99,6

CMC-50-181-C2A7	CCT117727	$C_{17}H_{22}ClNO_2$	307,81508	107,7	64,0	59	99,2
CMC-50-181-C2A8	CCT117800	$C_{21}H_{20}ClFN_2O_2$	386,8471032	135,4	12,4	9	100,0
CMC-50-181-C2A9	CCT117729	$C_{22}H_{23}ClN_2O_3$	398,88262	139,6	10,0	7	94,7
CMC-50-181-C3A1	CCT117698	$C_{16}H_{19}NO_3$	273,32696	95,7	69,1	72	97,0
CMC-50-181-C3A10	CCT117756	$C_{22}H_{22}Cl_2N_2O_2$	417,32828	146,1	51,6	35	100,0
CMC-50-181-C3A11	CCT117710	$C_{18}H_{22}N_2O_3$	314,37888	110,0	55,6	51	99,4
CMC-50-181-C3A12	CCT117764	$C_{26}H_{25}NO_2$	383,4822	134,2	70,5	53	99,7
CMC-50-181-C3A13	CCT117796	$C_{20}H_{21}NO_2$	307,38624	107,6	65,6	61	100,0
CMC-50-181-C3A14	CCT117778	$C_{19}H_{25}NO_2$	299,4073	104,8	18,9	18	100,0
CMC-50-181-C3A15	CCT117606	$C_{16}H_{17}NO_2$	255,31168	89,4	20,3	23	0,0
CMC-50-181-C3A16	CCT117788	$C_{22}H_{24}N_2O_2$	348,43816	122,0	41,0	34	100,0
CMC-50-181-C3A2	CCT117762	$C_{17}H_{21}NO_2$	271,35414	95,0	32,1	34	99,6
CMC-50-181-C3A3	CCT117784	$C_{20}H_{29}NO_2$	315,44976	110,4	61,0	55	99,7
CMC-50-181-C3A4	CCT117639	$C_{18}H_{21}NO_2$	283,36484	99,2	47,0	47	99,5
CMC-50-181-C3A5	CCT117678	$C_{16}H_{19}NO_2$	257,32756	90,1	43,1	48	99,6
CMC-50-181-C3A6	CCT117776	$C_{18}H_{25}NO_4$	319,3954	111,8	63,7	57	99,2
CMC-50-181-C3A7	CCT117792	$C_{18}H_{25}NO_2$	287,3966	100,6	59,1	59	100,0
CMC-50-181-C3A8	CCT117772	$C_{22}H_{23}FN_2O_2$	366,4286232	128,3	17,0	13	100,0
CMC-50-181-C3A9	CCT117782	$C_{23}H_{26}N_2O_3$	378,46414	132,5	25,2	19	96,5
CMC-50-181-C4A1	CCT117805	$C_{18}H_{17}NO_3$	295,33248	103,4	40,5	39	97,9
CMC-50-181-C4A10	CCT117381	$C_{24}H_{20}Cl_2N_2O_2$	439,3338	153,8	40,9	27	99,8
CMC-50-181-C4A11	CCT117806	$C_{20}H_{20}N_2O_3$	336,3844	117,7	31,7	27	100,0
CMC-50-181-C4A12	CCT117797	$C_{28}H_{23}NO_2$	405,48772	141,9	27,5	19	98,8
CMC-50-181-C4A13	CCT117803	$C_{22}H_{19}NO_2$	329,39176	115,3	46,7	41	99,1
CMC-50-181-C4A14	CCT117693	$C_{21}H_{23}NO_2$	321,41282	112,5	39,3	35	100,0
CMC-50-181-C4A15	CCT117668	$C_{18}H_{15}NO_2$	277,3172	97,1		0	
CMC-50-181-C4A16	CCT117573	$C_{24}H_{22}N_2O_2$	370,44368	129,7	39,1	30	99,7
CMC-50-181-C4A2	CCT117716	$C_{19}H_{19}NO_2$	293,35966	102,7	40,8	40	100,0
CMC-50-181-C4A3	CCT117701	$C_{22}H_{27}NO_2$	337,45528	118,1	49,3	42	100,0
CMC-50-181-C4A4	CCT117396	$C_{20}H_{19}NO_2$	305,37036	106,9	28,7	27	100,0
CMC-50-181-C4A5	CCT117697	$C_{18}H_{17}NO_2$	279,33308	97,8	26,7	27	100,0

CMC-50-181-C4A6	CCT117347	$C_{20}H_{23}NO_4$	341,40092	119,5	39,9	33	99,7
CMC-50-181-C4A7	CCT117793	$C_{20}H_{23}NO_2$	309,40212	108,3	43,3	40	100,0
CMC-50-181-C4A8	CCT117789	$C_{24}H_{21}FN_2O_2$	388,4341432	136,0	43,6	32	99,3
CMC-50-181-C4A9	CCT117714	$C_{25}H_{24}N_2O_3$	400,46966	140,2	66,6	48	75,8
CMC-50-189-C5A1	CCT117310	$C_{15}H_{17}NO_3$	259,30038	90,8		0	
CMC-50-189-C5A10	CCT117357	$C_{21}H_{20}Cl_2N_2O_2$	403,3017	141,2	47,1	33	99,0
CMC-50-189-C5A11	CCT117330	$C_{17}H_{20}N_2O_3$	300,3523	105,1	47,9	46	99,5
CMC-50-189-C5A12	CCT117316	$C_{25}H_{23}NO_2$	369,45562	129,3	55,7	43	98,1
CMC-50-189-C5A13	CCT117359	$C_{19}H_{19}NO_2$	293,35966	102,7	55,2	54	99,5
CMC-50-189-C5A14	CCT117383	$C_{18}H_{23}NO_2$	285,38072	99,9		0	
CMC-50-189-C5A15	CCT117371	$C_{15}H_{15}NO_2$	241,2851	84,4		0	
CMC-50-189-C5A16	CCT117344	$C_{21}H_{22}N_2O_2$	334,41158	117,0	15,7	13	96,2
CMC-50-189-C5A2	CCT117309	$C_{16}H_{19}NO_2$	257,32756	90,1		0	
CMC-50-189-C5A3	CCT117370	$C_{19}H_{27}NO_2$	301,42318	105,5		0	
CMC-50-189-C5A4	CCT117377	$C_{17}H_{19}NO_2$	269,33826	94,3	36,7	39	96,4
CMC-50-189-C5A5	CCT117362	$C_{15}H_{17}NO_2$	243,30098	85,2	25,6	30	85,4
CMC-50-189-C5A6	CCT117326	$C_{17}H_{23}NO_4$	305,36882	106,9	38,9	36	97,2
CMC-50-189-C5A7	CCT117335	$C_{17}H_{23}NO_2$	273,37002	95,7	50,8	53	98,6
CMC-50-189-C5A8	CCT117375	$C_{21}H_{21}FN_2O_2$	352,4020432	123,3	17,5	14	97,4
CMC-50-189-C5A9	CCT117345	$C_{22}H_{24}N_2O_3$	364,43756	127,6	48,3	38	99,8
CMC-50-189-C6A1	CCT117358	$C_{16}H_{19}NO_3$	273,32696	95,7	10,8	11	97,6
CMC-50-189-C6A10	CCT116835	$C_{22}H_{22}Cl_2N_2O_2$	417,32828	146,1		0	
CMC-50-189-C6A11	CCT117394	$C_{18}H_{22}N_2O_3$	314,37888	110,0	13,2	12	97,5
CMC-50-189-C6A12	CCT117349	$C_{26}H_{25}NO_2$	383,4822	134,2		0	
CMC-50-189-C6A13	CCT117092	$C_{20}H_{21}NO_2$	307,38624	107,6	52,2	49	99,8
CMC-50-189-C6A14	CCT117386	$C_{19}H_{25}NO_2$	299,4073	104,8		0	
CMC-50-189-C6A15	CCT117392	$C_{16}H_{17}NO_2$	255,31168	89,4	2,1	2	56,3
CMC-50-189-C6A16	CCT117368	$C_{22}H_{24}N_2O_2$	348,43816	122,0	1,5	1	92,6
CMC-50-189-C6A2	CCT117314	$C_{17}H_{21}NO_2$	271,35414	95,0	8,4	9	94,2
CMC-50-189-C6A3	CCT117382	$C_{20}H_{29}NO_2$	315,44976	110,4		0	
CMC-50-189-C6A4	CCT117364	$C_{18}H_{21}NO_2$	283,36484	99,2	53,3	54	96,3

CMC-50-189-C6A5	CCT117380	$C_{16}H_{19}NO_2$	257,32756	90,1	21,6	24	94,0
CMC-50-189-C6A6	CCT117366	$C_{18}H_{25}NO_4$	319,3954	111,8	50,8	45	98,5
CMC-50-189-C6A7	CCT117322	$C_{18}H_{25}NO_2$	287,3966	100,6	28,5	28	96,4
CMC-50-189-C6A8	CCT117308	$C_{22}H_{23}FN_2O_2$	366,4286232	128,3	4,1	3	81,4
CMC-50-189-C6A9	CCT117353	$C_{23}H_{26}N_2O_3$	378,46414	132,5	3,2	2	70,6
CMC-50-189-C7A1	CCT117346	$C_{15}H_{17}NO_4$	275,29978	96,4	61,8	64	99,8
CMC-50-189-C7A10	CCT117343	$C_{21}H_{20}Cl_2N_2O_3$	419,3011	146,8	31,6	22	96,1
CMC-50-189-C7A11	CCT117328	$C_{17}H_{20}N_2O_4$	316,3517	110,7	55,3	50	99,2
CMC-50-189-C7A12	CCT117342	$C_{25}H_{23}NO_3$	385,45502	134,9	66,4	49	99,3
CMC-50-189-C7A13	CCT117378	$C_{19}H_{19}NO_3$	309,35906	108,3	62,2	57	99,8
CMC-50-189-C7A14	CCT117332	$C_{18}H_{23}NO_3$	301,38012	105,5	22,6	21	98,4
CMC-50-189-C7A15	CCT117400	$C_{15}H_{15}NO_3$	257,2845	90,0	5,6	6	17,2
CMC-50-189-C7A16	CCT117379	$C_{21}H_{22}N_2O_3$	350,41098	122,6	1,4	1	82,4
CMC-50-189-C7A2	CCT117320	$C_{16}H_{19}NO_3$	273,32696	95,7	59,5	62	98,2
CMC-50-189-C7A3	CCT117323	$C_{19}H_{27}NO_3$	317,42258	111,1	62,9	57	99,5
CMC-50-189-C7A4	CCT117398	$C_{17}H_{19}NO_3$	285,33766	99,9	48,3	48	99,5
CMC-50-189-C7A5	CCT117334	$C_{15}H_{17}NO_3$	259,30038	90,8	8,2	9	90,4
CMC-50-189-C7A6	CCT117324	$C_{17}H_{23}NO_5$	321,36822	112,5	76,1	68	93,4
CMC-50-189-C7A7	CCT117311	$C_{17}H_{23}NO_3$	289,36942	101,3	58,8	58	99,8
CMC-50-189-C7A8	CCT117390	$C_{21}H_{21}FN_2O_3$	368,4014432	128,9	5,3	4	96,7
CMC-50-189-C7A9	CCT117331	$C_{22}H_{24}N_2O_4$	380,43696	133,2	48,3	36	97,9
CMC-50-189-C8A1	CCT117338	$C_{15}H_{16}ClNO_4$	309,74484	108,4	25,5	24	96,0
CMC-50-189-C8A10	CCT117365	$C_{21}H_{19}Cl_3N_2O_3$	453,74616	158,8	16,5	10	92,6
CMC-50-189-C8A11	CCT117102	$C_{17}H_{19}ClN_2O_4$	350,79676	122,8	19,1	16	92,8
CMC-50-189-C8A12	CCT117361	$C_{25}H_{22}ClNO_3$	419,90008	147,0	35,6	24	93,7
CMC-50-189-C8A13	CCT117307	$C_{19}H_{18}ClNO_3$	343,80412	120,3		0	
CMC-50-189-C8A14	CCT117376	$C_{18}H_{22}ClNO_3$	335,82518	117,5	22,1	19	100,0
CMC-50-189-C8A15	CCT117374	$C_{15}H_{14}ClNO_3$	291,72956	102,1	2,9	3	29,1
CMC-50-189-C8A16	CCT117329	$C_{21}H_{21}ClN_2O_3$	384,85604	134,7	2,0	1	82,5
CMC-50-189-C8A2	CCT117333	$C_{16}H_{18}ClNO_3$	307,77202	107,7	27,6	26	97,0
CMC-50-189-C8A3	CCT117355	$C_{19}H_{26}ClNO_3$	351,86764	123,2	48,1	39	96,1

CMC-50-189-C8A4	CCT117337	$C_{17}H_{18}ClNO_3$	319,78272	111,9	39,3	35	100,0
CMC-50-189-C8A5	CCT117317	$C_{15}H_{16}ClNO_3$	293,74544	102,8	20,5	20	95,9
CMC-50-189-C8A6	CCT117341	$C_{17}H_{22}ClNO_5$	355,81328	124,5	56,5	45	100,0
CMC-50-189-C8A7	CCT117336	$C_{17}H_{22}ClNO_3$	323,81448	113,3	50,7	45	100,0
CMC-50-189-C8A8	CCT117372	$C_{21}H_{20}ClFN_2O_3$	402,8465032	141,0	13,0	9	98,3
CMC-50-189-C8A9	CCT117339	$C_{22}H_{23}ClN_2O_4$	414,88202	145,2	47,0	32	92,9
